# Ocular changes as potential biomarkers for early diagnosis of Alzheimer's disease

**DOI:** 10.1002/alz.70476

**Published:** 2025-08-24

**Authors:** Purna Chandra Poudel, Shaun M. Frost, Shaun Eslick, Hamid R. Sohrabi, Kevin Taddei, Eugene Hone, Ralph N Martins

**Affiliations:** ^1^ Alzheimer's Research Australia, Ralph and Patricia Sarich Neuroscience Research Institute QEII Medical Centre Nedlands Western Australia Australia; ^2^ Centre of Excellence for Alzheimer's Disease Research and Care, School of Medical and Health Sciences Edith Cowan University Joondalup Western Australia Australia; ^3^ Health and Biosecurity Commonwealth Scientific and Industrial Research Organisation (CSIRO) Kensington Western Australia Australia; ^4^ Digital Therapeutics and Care Australian e‐Health Research Centre Kensington Western Australia Australia; ^5^ Macquarie Medical School Macquarie University Macquarie Park New South Wales Australia; ^6^ Centre for Healthy Ageing Health Futures Institute Murdoch University Murdoch Western Australia Australia; ^7^ Lions Alzheimer's Foundation Nedlands Western Australia Australia

**Keywords:** Alzheimer's disease, amyloid beta, biomarkers, brain, early detection, ocular, retina

## Abstract

Alzheimer's disease (AD) is a progressive neurodegenerative disorder and the most common cause of dementia. AD diagnosis often involves a thorough assessment, including clinical evaluation, cognitive testing, medical history examination, genetic testing, and biomarker analysis. Currently, the invasive nature and high costs of biomarker testing, such as cerebrospinal fluid analysis and neuroimaging, have limited the early detection and intervention of the disease. Therefore, researchers have explored cheaper and less invasive options, such as ocular imaging. This review discusses the close relationship between the eye and the central nervous system and ocular changes as a potential non‐invasive AD biomarker. Ocular changes in AD have frequently been reported in the literature, particularly in the later stages of the disease. However, identifying biomarkers specifically attributable to AD remains a challenge. Additionally, this review provides a comprehensive overview of existing studies and highlights potential pathways for enhancing AD detection through ocular structural and functional evaluation.

**Highlights:**

- Widespread screening for early detection of Alzheimer's disease (AD) is limited by cost and time constraints with current methods.
- Non‐invasive and inexpensive methods are needed to overcome these difficulties.
- The eye is readily accessible and has shared developmental origins, neurobiology, and neurochemistry with the brain.
- The expanding field of ocular biomarker studies needs larger, well‐characterized cohorts and longitudinal studies to understand the ocular changes for preclinical AD screening.
- Standards should be established for more methodical investigative approaches to identify ocular biomarkers that are specifically attributable to AD.

## 1 INTRODUCTION

In 2023, it was estimated that > 55 million people worldwide had dementia (according to the World Health Organization),^1^ making it the seventh leading cause of death and a crucial factor in global aging‐related disability and dependency. Alzheimer's disease (AD) dominates in prevalence and is irreversible and progressive, contributing to ≈ 60% to 80% of all dementia cases. Age is the biggest risk factor,^2^ with multiple others, including genetic, environmental, and lifestyle factors, known to have a significant impact on disease development.^3^

Although AD is diagnosed *post mortem* by the presence of extracellular amyloid beta (Aβ) plaques and tau intracellular tau tangles in neurons,^4^ the disease is difficult to diagnose *ante mortem* due to the brain being progressively damaged.^5^ Although several disease‐modifying medications are being tested,^6^, ^7^ monitoring the response is costly with current approaches. It is widely acknowledged that early detection is valuable to maximize the effects of interventions before irreversible degeneration occurs.^8^ However, the current de facto gold standard diagnostic tools, namely positron emission tomography (PET) brain Aβ imaging^9^, ^10^ and cerebrospinal fluid (CSF) biomarkers,^11^ are still very costly and are being further developed. Non‐invasive AD detection has since been a major goal of the field. Although approaches using blood‐based biomarkers promise to be inexpensive, widely available, and minimally invasive, they still currently require significant development. A more recent approach that has gained attention is retinal imaging due to its largely non‐invasive and inexpensive nature. It has the potential to be used solely or in combination with blood‐based biomarkers to identify pre‐clinical AD accurately.

The eye is a complex structure with direct neural connections to the brain, as illustrated by dense innervations of sensory and motor neurons. Out of the 12 cranial nerves (CNs), four are associated with the eye (CN II, III, IV, and VI). The optic nerve (CN II) transmits sensory information from the retina to the brain.^12^, ^13^ The CN III and related fibers innervate the eye muscles, pupil, and eyelid that control eye movements (EMs) and support sensory functioning. The CN IV, a motor neuron, innervates the superior oblique muscles and controls downward, outward, and inward eye rotation. Additionally, the ophthalmic branch of CN V innervates the ocular surface, eyebrows, eyelids, iris, and lacrimal gland.^14^ Furthermore, the retina and optic nerve are formed as extensions of the growing embryonic diencephalon. It is, therefore, derived from the same embryonic cell group that eventually forms the brain. Due to these shared similarities with the brain, the eye is often considered a part of the central nervous system (CNS).^15^

**FIGURE 1:**
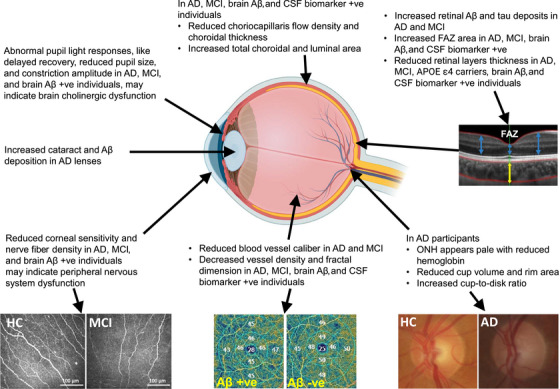
AD biomarkers of the eye. Various ocular biomarkers have demonstrated changes in many cohorts. Ocular imaging has shown changes in pupillary response, corneal nerve morphometry,^16^ retinal structure, blood vessels, and ONH parameters.^17^ These findings support the potential of using the eye as a non‐invasive biomarker site for AD screening or diagnosis. +ve, positive; ˗ve, negative; AD, Alzheimer's disease; *APOE*, apolipoprotein E; Aβ, amyloid beta; CSF, cerebrospinal fluid; FAZ, foveal avascular zone; HC, healthy control; MCI, mild cognitive impairment; ONH, optic nerve head.

Indeed, the eye contains an abundant supply of neurons and reflects the manifestation of many neuronal changes in the CNS. From a biomarker and diagnostic perspective, it is significantly more accessible than the brain, making it a good candidate for non‐invasive imaging techniques. Several ocular changes, such as retinal abnormalities, optic nerve alterations, and changes to EM, have been observed in people with AD (Figure 1). These ocular biomarkers are being investigated as potential inexpensive and non‐invasive methods for early detection and AD monitoring.

This review will provide an overview of the most significant studies on ex vivo examinations, eye tracking, and in vivo imaging of several eye structures, including the pupil, lens, retina, choroid, cornea, and optic nerve head (ONH). It is based on a comprehensive literature search conducted between March 2023 and November 2024 using the Google Scholar, Web of Science, and PubMed databases. The search strategy used a combination of keywords to identify pertinent research articles, including “Alzheimer's disease,” “eye,” “retina,” “lens,” “pupil,” “cornea,” “choroid,” “eye movement,” “optic disc,” “optic nerve head,” “histopathological study,” “tau,” and “amyloid.” Additional terms and Boolean operators were used as needed to refine the search and ensure comprehensive coverage of the literature. Original research articles, systematic reviews, and meta‐analyses focusing on structural, functional, and histopathological ocular changes in AD were included.

## 2 PUPILLARY RESPONSE

The iris controls light levels incident on the retina by regulating the size of its central aperture, the pupil. This change in pupil size due to light or other stimuli (e.g., emotions, cognitive processes, pain, and certain medications) is known as the pupillary response.^18^ The pupil light reflex (PLR) is a parasympathetic cholinergic response of the pupil in response to light.^19^ The results of most pupillary response studies were consistent with cholinergic deficit in AD,^20^, ^21^ displaying altered PLR parameters. These studies have consistently found smaller resting pupil diameter,^22^, ^23^ reduced amplitude,^20^, ^22^, ^23^, ^24^, ^25^, ^26^ lower pupillary response velocity and acceleration,^20^, ^23^, ^25^, ^26^ and higher constriction latency^23^, ^25^, ^26^ in AD cases compared to healthy controls (HCs). Similarly, the recovery time of the pupil from the pupil flash response was slower in amyloid precursor protein (*APP*) mutation carriers (*APPGlu693Gln*) compared to non‐carriers.^27^ A longitudinal analysis^20^ of maximum pupil constriction velocity and acceleration showed significant reductions over a 3 year period in high brain Aβ load individuals, but not in low brain Aβ individuals. A recent study with a comparably larger sample size included 107 AD‐affected individuals, 44 with mixed AD and vascular cognitive dysfunction, 53 with Lewy body dementia (LBD), and 50 HCs.^23^ Their diagnostic models exhibited comparable discriminatory performance, achieving an area under the receiver operating curve (AUC) between 0.74 and 0.81 when differentiating AD from HCs and other dementia subtypes, respectively.

However, some earlier PLR studies could not detect significant differences in resting pupil diameters,^24^, ^25^, ^28^ constriction amplitude,^29^ constriction latency,^29^ and constriction velocity and acceleration^27^ between cases and control groups. The inconsistencies in the results may be attributed to the absence of age matching between the patient and control groups,^20^, ^23^, ^24^, ^30^ variations in light wavelength, and the duration of light exposure. It is important to note that the majority of previous studies that have investigated the PLR in AD have not taken into consideration baseline pupil diameter normalization, which complicates direct comparisons between subjects. Additionally, the age‐related decrease in pupil diameter^31^, ^32^ and severity of AD may function as a potential confounding variable.

Flash pupillometry or PLR has undergone recent improvements to enhance its sensitivity, including repetitive light stimulation and chromatic pupillometry. Repetitive flash pupillometry uses brief flashes of light delivered at regular intervals, while chromatic pupillometry measures pupillary parameters using different colors of light to assess light‐sensitive retinal neuronal cell dysfunction. In a repeating flash study,^30^ AD and mild cognitive impairment (MCI) groups were reported to have a less pronounced pupil size attrition and amplitude increase over time than controls. This study also found an association between higher Mini‐Mental State Examination (MMSE) scores of the participants with an increase in amplitude and a decrease in latency. Chromatic pupillometry makes it possible to isolate the contribution of rods, cones, and melanopsin‐expressing retinal ganglion cells (mRGCs) to the PLR. One study^33^ showed that pre‐symptomatic AD individuals had more variability in melanopsin function to the blue stimulus. However, no statistically significant differences in pupil response parameters to both red and blue‐colored stimuli were found. A later study^34^ showed that participants with AD had significantly smaller baseline pupil size and non‐significantly lower contraction amplitudes to blue and red light. Another illumination‐adjusted chromatic pupillometry study^35^ reported a significant difference between transient peak amplitude (difference between the normalized baseline pupil size and maximum pupil constriction) of AD and HCs for the rod and melanopsin conditions (*P* < 0.01) but not for the cone condition. These chromatic pupillometry studies have been limited by sample size and could not show a clear mRGC‐driven pupil dysfunction between individuals with early AD and the control group.

Task‐evoked pupillary responses have also been investigated in AD^36^, ^37^, ^38^ to gain insight into cognitive changes and AD‐associated dysfunction of the locus coeruleus, the primary site of noradrenalin production in the brain.^39^ Variations in pupil size during task‐evoked pupillary responses are also assumed to be associated with AD‐related changes in neural processes.^36^ One cognitive task‐guided pupil response study recorded pupillary responses during digit span recall. Their result showed that amnestic MCI participants had more pupil dilation than non‐amnestic MCI and cognitively unimpaired healthy participants at lower cognitive load.^37^ Another study hypothesized that abnormal pupillary responses would be associated with middle‐aged cognitively normal adults with higher genetic risk for AD (based on AD polygenic risk scores). They subsequently demonstrated that the pupil dilatation of these higher‐risk individuals was significantly higher during periods of high cognitive load.^38^ In a similar memory‐guided digit span task study, fewer variations in pupil size (lower change in the pupil size) across the different tasks, such as forward span, backward span, and counting of digits, were detected in AD individuals compared to the controls.^36^ A recent study^40^ demonstrated distinct pupil variables (maximum pupil dilation, maximum pupil constriction, and changes in pupil size) between HC and MCI participants in prosaccade and antisaccade oculomotor tasks. During tasks, HC participants exhibited predictive pupil metrics suggesting an intact oculomotor system supporting efficient visual processing, whereas MCI participants demonstrated deficits in visual processing. Although the results of task‐evoked pupillometry are interesting as a practical and non‐invasive ocular biomarker in AD, precise pupillary measurement using sophisticated computer‐based paradigms in a large cohort could offer a better insight into the cognitive processing in AD.

In summary, the pupillary response studies in AD seem to have limited sample sizes and show variable results (see Table 1 and Table S1 in supporting information). The light flashes are sometimes not well tolerated and cause subjects to blink,^33^ which could limit the screening ability. Assessing pupillary responses may still be useful to quickly and inexpensively evaluate the cholinergic system of the CNS. However, further studies with the application of recent computer technologies are needed to establish consistent findings and better understand the role of pupillary response in underlying AD mechanisms.

**TABLE 1:** Summary of in vivo ocular studies highlighting group differences between individuals with clinical MCI/AD and healthy controls, differences between biomarker‐confirmed AD cases and controls. Studies reporting null findings and associated study limitations are also included.

Ocular Study	Alteration	Studies demonstrating significant differences for MCI/AD versus HC	Studies demonstrating null results for MCI/AD versus HC	Studies demonstrating significant differences for biomarker positive versus HC	Studies demonstrating null results for biomarker positive versus HC	Limitations
Pupillary responses	Pupillary flash response parameters: pupil size, maximum pupil constriction, constriction velocity, dilation velocity, latency and amplitude	30; 29 #; 25 #; 26 #; 24 #; 22 #; 23 #		27 σ; 20 # α m		**a, b, c**
	Chromatic pupillometric variables: baseline and peak pupil size, contraction amplitude, sustained response, mean PLR, and PLR variability	34 #		33 # γ		
	Task‐evoked pupillary response: pupil dilation, constriction magnitude, and pupil size during tasks	36; 38; 40 #				
Lens	Aβ aggregation in the lens, supranuclear cataract	56; 57			58 α; 49 μ	**a, b, c**
Cornea	Corneal nerve fiber density, branch density, and fiber length using corneal confocal microscope	66; 67 #; 16 #; 71 # m; 72				**a, b, c**
	Corneal thickness using cSLO		68			
	Corneal dendritic cell field area and perimeter using corneal confocal microscope	69 #				
	Corneal sensitivity using corneal esthesiometer	64; 72				
Saccadic EM	Prosaccade	91; 74 #; 75 #; 92 #; 93 # λ; 76 #; 94 #; 104 #; 77; 107 #; 95 #; 105; 96; 97 #; 78 #; 89 #; 98 #.	106; 99; 100; 79			**b, c**
	Antisaccade	106 #; 74 #; 75; 92 #; 99 #; 101 #; 76 #; 94 #; 104 #; 77; 105 #; 79 #; 78 #; 80 #; 98 #.				
	Smooth pursuit	74 #; 76 #; 77 #; 96; 89	98			
Choroid	Choroidal thickness	114 #; 115 #; 116; 117 #; 109; 118; 110 #; 121 #; 119 m		110 #	120 α;	**a, b, c**
	Choroidal blood flow	130	116; 111	131 α		
	Total choroidal area, luminal area and choroidal vascularity index	112 #			126 δ λ	
Retinal structural changes	pRNFL	17 #; 146 #; 68; 147 #; 148; 149 #; 150; 151 #; 152 #; 153; 154; 155 #; 156 #; 157 #; 140 #; 158 #; 159 #; 160; 161 #; 162 # m; 163; 164; 165 #; 166 # λ; 167 # λ; 168 #; 169; 170 λ; 171 #; 172 #; 173 # λ; 174 #; 175 #; 176 #; 177 #; 178 #; 179 #; 180	109; 184; 191; 192; 187; 186; 193; 194	181 γ; 182 α #.	186 α; 195 α; 196 α λ; 197 α; 145 δ; 126 δ m; 190 α m	**c**
	mRNFL	150; 152; 165 #; 184 #; 185 #; 214 λ	198; 111 #	190 α λ #; 145 δ #	195 α; 196 α λ; 197 α; 198 δ	
	GCL	152; 183; 165 #; 186; 111 #; 214 λ	198		145 δ; 195 α; 196 α λ; 197 α; 186 α; 198 δ;190 α m	
	GCL‐IPL	149; 155 #; 162 # m; 185 #; 172; 173 # λ; 174 #; 187 #	199; 192; 111		181 γ; 126 δ m; 182 α	
	RNFL‐GCL‐IPL	148; 149; 151; 114; 188; 170; 189; 111 #				
	IPL	152; 111 #; 214 λ	165 #; 198	190 α λ #; 145 δ #; 195 α	196 α λ; 197 α; 198 δ	
	INL		165 #; 152; 198; 214 λ	145 δ #;	195 α; 198 δ; 190 α m	
	ONL	152 #; 165 #; 185	198	190 α λ #	195 α; 145 δ; 198 δ;	
	OPL	165 #; 214 λ	152; 198	145 δ #; 190 α m #	195 α; 198 δ	
	RPE	214 λ	152; 165; 198		145 δ; 198 δ	
	Macular thickness	149 #; 154; 161 #; 165 #; 171 #; 173; 185 #; 111 #;	168; 147	182 α #	181 γ	
	Macular volume	148; 154; 171 #; 173; 175	147; 192			
	Foveal thickness	149; 171; 188; 165; 111;	158; 164; 168	200 α or γ;	182 α; 126 δ m	
	FAZ area	116; 169; 170 λ; 201	202; 121; 203; 204; 187; 198 #; 189	200 α or γ; 131 γ	120 α; 205 α; 126 δ; 198 δ	
Retinal vascular changes	Retinal vascular density	116; 199 #		205 α; 226 δ	226 α; 120 α	**b, c**
	Superficial capillary plexus microvascular density	169; 203; 199 #; 202 #; 168 #; 203; 170 # λ; 198 #; 223 #; 224 #; 204 #; 187 #; 189 #	199; 201	131 γ	198 δ; 126 δ m	
	Deep capillary plexus microvascular density	203 #; 199 #; 169; 170 # λ; 189 #; 223 #; 201; 168 #	202; 203; 198; 204	198 δ;	131 γ	
	Decreased blood flow	146; 191	116			
	Reduced perfusion density	224 #; 187 #	203; 111	131 # γ	126 δ m	
	Altered microvascular network	203 #; 223 #; 229 #; 230 #; 231 #; 232 #; 233 #		131 # γ; 120 # α		
	Oxygen saturation of hemoglobin	234				
	Venule diameter changes	146; 191; 230 #; 231; 232; 235 # λ	229 #; 237 λ			
	Arterial diameter changes	233; 235 # λ	229 #	236 α		
	Increased vascular tortuosity	231	229; 230; 235 λ	236 α	120 α	
	Reduced vascular tortuosity	232; 233				
ONH changes	Hemoglobin level	17	121			**a, b, c**
	Paler	17	246			
	Cup shape	246; 180	68; 169; 194			
	Vessel density around ONH		247	205 α		
Pathological changes	Changes in reflectance spectra captured by hyperspectral imaging	270 #; 271 #;		236 # α; 272 # α; 277 # α		**a, b, c**
	Changes captured through CSLO or fundus imaging	241 #; 267 #; 268 #; 269 #; 193 #; 235 # λ				

*Notes*: #, ocular parameters showed mixed results: some measures or regions differed significantly, while not in others; μ, study on participants with Down syndrome but not compared to HC; a, low literature volume; b, fewer number of studies with pre‐clinical AD participants; c, more longitudinal studies are needed; m, finding is based on both cross‐section and longitudinal studies; α, brain Aβ positive vs. brain Aβ negative; γ, CSF biomarker positive vs. control; δ, apolipoprotein E ε4 carriers vs. non‐carrier; λ, finding is based on longitudinal study; σ, APPGlu693Gln carriers vs. non‐carrier.

Abbreviations: Aβ, amyloid beta; AD, Alzheimer's disease; CSLO, confocal scanning laser ophthalmoscopy; EM, eye movement; FAZ, foveal avascular zone; HC, healthy control; INL, inner nuclear layer; IPL, inner plexiform layer; MCI, mild cognitive impairment; mRNFL, macular retinal nerve fiber layer; ONH, optic nerve head; ONL, outer nuclear layer; OPL, outer plexiform layer; PLR, pupillometric light response; pRNFL, peripapillary retinal nerve fiber layer; RPE, retinal pigment epithelium; RNFL, retinal nerve fiber layer.

## 3 LENS

The lens is primarily composed of crystallin proteins, whose main function is to refract incident light from outside the eye and focus it onto the retina.^41^ Degeneration of the lens occurs with aging, during which the most common condition is presbyopia, which is mostly due to reduced lens elasticity.^42^ Cataracts, the gradual clouding of the lens, are also common in the aged lens.^43^ The loss of optical transparency is due to an accumulation of misfolded, insoluble proteins,^41^, ^43^, ^44^ with many contributing factors. Indeed, this condition has also been thought to be influenced by the aggregation of other proteins that were observed in individuals with AD,^45^, ^46^ indicating that the lens may be an ideal site for identifying protein accumulation and potential AD markers.

APP and Aβ were found in oxidative stress‐induced cataracts in the interior fiber cells of cultured monkey lenses.^45^ Similarly, transgenic mouse models of AD (Tg2576) showed AD‐associated human Aβ lens pathology, age‐dependent supranuclear cataract phenotype, and increased expression of human APP in the lens while in non‐transgenic mice these were absent.^47^ These findings provide additional evidence that overproduction of Aβ can also lead to pathological alterations in the eye that mirror those observed in the brain.^48^

The presence of APP and Aβ deposition in the supranuclear region was observed in the AD‐affected *post mortem* human lens,^46^ using slit‐lamp photo microscopy, mass spectrometry, and immunohistochemistry. In a follow‐up study using the same techniques, the presence of Aβ was confirmed in the *post mortem* lenses of trisomy 21, Down syndrome (DS) individuals.^49^ The extra copy of chromosome 21 can produce 1.5 times as many APPs as healthy individuals, increasing Aβ deposition in the brain, often resulting in early‐onset AD.^49^, ^50^

However, not all studies found AD pathological proteins in the lens. The reasons for these conflicting findings remain unclear but could reflect substantial differences in the study methodology. Critically, these studies involved immunohistochemical analyses^51^, ^52^, ^53^, ^54^ in clinically and neuropathologically diagnosed AD participants. The variations in staining quality, such as the application of Bennhold's^46^ versus Puchtler's^51^ staining techniques and different immunostaining antibodies, may partially account for the inconsistencies. Additionally, factors such as *post mortem* interval, cataract classification, tissue fixation methods, and whole‐mount preparations may further contribute to these discrepancies.^55^ Moreover, cataract formation in the lens is influenced by multiple complex factors, and its relationship with AD brain pathology is yet to be determined.

One exploratory study trialled a fluorescent ligand with affinity for Aβ, both in vivo and in vitro.^56^ The technique detected increasing levels of ligands bound to Aβ deeper into the supranuclear region of the lens of AD individuals. In their subsequent study with a larger sample size, the same research team used this technique to discriminate probable AD individuals from age‐matched healthy volunteers.^57^ In addition, they demonstrated a significant correlation with PET brain Aβ load and concluded that this technique could be used safely for non‐invasive AD detection and severity assessment. A later in vivo study using the Scheimpflug imaging method to measure lens opacity could not accurately identify Aβ‐positive individuals.^58^ This outcome could be attributed to the reduced sensitivity of the Scheimpflug instrument, which does not use fluorescence techniques. Although fluorescent labelling of Aβ in the lens may provide increased sensitivity compared to conventional methods, the studies have limited sample sizes (see Table 1 and Table S2 in supporting information for a comprehensive overview). Therefore, more research is required to validate findings in larger, more well‐characterized cohorts. Likewise, ongoing research is yet to establish how closely Aβ and other protein accumulations in the lens follow brain AD pathology and disease severity.

## 4 CORNEA

The cornea is the transparent exterior layer of the eye, which serves as the first refractive layer and protection for the sensitive anterior chamber. It is densely innervated by C‐ and A‐delta fibers^59^, ^60^ originating from the ophthalmic branch of the trigeminal nerve. This nerve is a part of the peripheral nervous system and is involved in facial and ocular sensations. Due to this dense innervation, changes in corneal nerve structure and density have been proposed as potential biomarkers for AD detection and monitoring. See Supplementary Section 1 and Figure S1 in supporting information for a brief overview.

Both animal and human models have been used to explore the relationship between corneal changes and AD. The proteins involved in AD pathogenesis have been detected in the cornea. An immunohistochemical mouse study^61^ determined that the transmembrane protein presenilin, a major player in the pathophysiology of AD, is present in the epithelial layers of the cornea. Further studies reported a high cytoplasmic expression of APP in the corneal epithelial cell layers.^62^ These findings were supported by evidence from human corneal fibroblasts and corneal epithelium, where expressed APP and associated APP processing proteins were reported.^63^ Additionally, proteins involved in Aβ degradation were identified in both tissues. A clinical study^64^ tested corneal sensitivity in individuals with neurological conditions, such as AD, multiple sclerosis (MS), Parkinson's disease (PD), Friedreich's ataxia (FA), and epilepsy (EP). It was found that mean corneal sensitivity was significantly less in AD individuals than in age‐ and sex‐matched controls (*p* = 0.001), and those with FA (*p* = 0.021), and MS (*p* = 0.016). However, no significant difference was observed compared to PD (*p* = 0.6) and EP individuals (*p* = 0.3).

More recently, it has become possible to examine the cornea at the cellular level using a technique known as corneal confocal microscopy (CCM). The application of this method has expanded significantly over the past few years for the non‐invasive and rapid clinical evaluation of corneal nerve fiber, particularly in diabetic peripheral neuropathy.^65^ However, only a few studies have been conducted to detect AD pathology using CCM.^16^, ^66^, ^67^, ^68^, ^69^, ^70^ They have used the HRT‐RCM (Heidelberg Retina Tomograph Rostock Corneal Module), a specialized scanning laser ophthalmoscope (SLO), for cellular‐level visualization of the cornea. Morphological assessments of corneal dendritic cells (DCs), the immune cells of this tissue, showed that individuals with MCI had a larger DC average field area and perimeter than HCs in the central and mid‐peripheral corneal regions, even though there were neither significant nerve abnormalities nor a difference in DC density.^69^ They concluded that changes in the corneal DC phenotype may occur before peripheral nerve degeneration in CNS neuropathology. It has also been demonstrated that corneal nerve fiber density, branch density, and fiber length were decreased in both MCI and dementia individuals compared to HCs.^66^, ^67^ Furthermore, this loss of corneal nerve fibers was associated with cognitive decline in MCI and dementia participants.^67^ The follow‐up study^66^ reported a superior prediction accuracy of CCM compared to the visual rating of medial temporal lobe atrophy through magnetic resonance imaging (MRI) for participants with MCI. Furthermore, the prediction accuracy was higher and comparable for dementia‐affected individuals.

Subsequently, using corneal nerve morphometry, MRI brain volumetry, and cognitive analysis in participants with 42 HC, 98 MCI, and 68 dementia patients, it was found that abnormalities in corneal parameters and MRI volumetry were associated with cognitive impairment.^16^ Also, the prediction ability of CCM (76% to 81% accuracy) compared to brain volumetry (52% to 70%) was higher for the identification of MCI and comparable for dementia (77% to 85% for CCM and 69% to 93% for MRI). In their longitudinal study, they observed reduced corneal nerve morphometry parameters, including nerve fiber density, branch density, and fiber length, in the cohort of MCI participants who converted to dementia (*n* = 33) after 2.6 years than in HCs (*n* = 12) at baseline and after 2.6 years.^71^ However, the decrease in corneal parameters was not statistically significant in the subset of MCI participants whose cognition remained stable (*n* = 74) from baseline through the 2.6 year study duration. In the AD cohort, a recent study^72^ demonstrated a significant reduction (each with *P* < 0.001) in corneal nerve fiber density, fiber length, and branch density compared to HCs using CCM. This study also reported a significant reduction in corneal sensitivity (*P* < 0.001). Despite being limited in quantity, findings from CCM research have shown potential for detecting neurodegeneration in individuals with cognitive impairment and identifying those with early or advanced stages of neurodegeneration.

The study of the cornea continues to be an interesting area of study in AD. It has the potential to both assist in current diagnostic techniques and provide the underlying causes of the condition. Studies showed promising evidence that corneal imaging measures appeared to be effective in predicting AD (please refer to Table 1 and Table S3 in supporting information for a comprehensive summary). However, these studies have only involved limited sample sizes with short follow‐up periods. There were also no precise diagnostic assessments or measurements of established AD biomarkers, such as PET brain Aβ or tau PET imaging, CSF Aβ or phosphorylated tau, CSF total tau, or fluorodeoxyglucose (FDG) PET hypometabolism. Integrating these AD biomarkers may enhance understanding of the accuracy and reliability of corneal changes as a potential biomarker for AD dementia.

## 5 EYE MOVEMENTS

Assessments of EM and oculomotor function have been investigated as potential methods for the early identification, differential diagnosis, and monitoring of AD.^73^ Although alterations in oculomotor function are not regarded as core indicators or symptoms of AD, some studies have shown that individuals with the disease have EM abnormalities.^74^, ^75^, ^76^, ^77^, ^78^ These changes vary widely depending on the stage and severity of the disease.^79^, ^80^, ^81^ The neural circuitry that controls EM may be uniquely affected by the pattern of neurodegeneration specific to AD, allowing for the clinical distinction of AD from other cognitive diseases.

Studies showed that MCI and AD can affect general gaze behavior and visual fixation. Individuals may exhibit reduced visual scanning patterns, a preference for particular visual regions, shorter fixation times, or more fixations. It may result in a restricted field of vision or a limited capacity to shift focus between different objects or locations.^82^, ^83^, ^84^ Gaze patterns were also shown to correlate with cognition.^82^ Furthermore, AD‐affected participants distributed their viewing time equally at regular and irregular shapes, while spending significantly less time viewing the novel stimuli.^83^ However, non‐AD controls spent significantly more time looking at the irregular shapes than regular ones. This finding indicated that diminished curiosity, which can be evaluated through EMs, is reflected in the gaze of AD participants.^83^

A study^84^ used a custom‐built eye‐tracking system for cognitive assessment and showed a strong correlation with the scores from the neuropsychological tests. It also demonstrated a good predictive performance in detecting individuals with MCI and dementia. A recent investigation agrees that cognitive impairment may be linked to gaze behavior.^85^ The study involved 37 individuals with no dementia, 49 with AD, and 20 with LBD. It found that visual behavior exhibited distinct correlations with cognitive impairment in AD and motor impairment in LBD, with significant differences between groups (*P* < 0.01). A classification model based on these features achieved an AUC of 0.76 for differentiating AD from HCs, 0.87 for LBD versus HCs, and 0.82 for AD versus LBD.

In the experiments with smooth pursuit EMs, dementia‐affected participants tracked the stimulus target with increased latency,^76^ reduced initial acceleration, decreased velocity, and decreased gain (the ratio of pursuit velocity to target velocity).^74^, ^76^, ^86^, ^87^ The gain in AD participants was reduced considerably at higher target velocities and lower target accelerations.^88^ In another study, the AD group had significantly lower smooth pursuit tracking efficiency than the non‐AD controls. Also, the visual tracking abnormality of AD participants was found to correlate strongly with dementia severity.^81^

Similarly, a few studies have analyzed the relationship between smooth pursuit and brain parameters. Some of these studies demonstrated no correlations between lobar gray matter volumes measured by MRI and smooth pursuit gain and latency in AD.^74^, ^89^ However, one study found an association between smooth pursuit error and reduced glucose metabolism measured by ^18^F‐FDG PET in the right posterior middle temporal gyrus, which is situated near the middle temporal complex.^77^ A potential explanation for this result is that the spatial normalization and resolution of the ^18^F‐FDG PET have a relatively low error and are more sensitive.

The majority of EM studies in AD research have analyzed saccade EM, a rapid EM between fixations. Visually guided saccades, also referred to as prosaccades and voluntary saccades, which include antisaccades, memory‐guided saccades, and predictive saccades, are the two main classes of saccadic EMs.^90^ Antisaccades are the reverse process of prosaccades, which occur when the gaze moves away from the peripheral target onset. In memory‐guided saccades, the peripheral target stimulus appears momentarily, and participants carry out saccadic EM from the central stimulus to the direction of the peripheral target stimulus. Participants in predictive saccades usually focus their gaze on anticipation of the appearance of an object in a specific location with a fixed temporal frequency (see Figure S2 in supporting information).^78^

Studies have used several conditions within these saccade paradigms, such as “gap,”^74^, ^75^, ^76^, ^77^, ^78^, ^79^, ^80^, ^89^, ^91^, ^92^, ^93^, ^94^, ^95^, ^96^, ^97^, ^98^, ^99^, ^100^, ^101^, ^102^, ^103^ “step,”^94^, ^104^, ^105^, ^106^ and “overlap.”^74^, ^75^, ^76^, ^79^, ^89^, ^92^, ^93^, ^97^, ^98^, ^99^, ^107^ The peripheral target stimulus in the gap condition emerged after the central fixation point disappeared within a certain period. In the step condition, the peripheral target stimulus appeared concurrently with the disappearance of the central fixation point, whereas, in the overlap condition, the central fixation stimulus is also perceptible after the peripheral target appearance (see Figure S3 in supporting information).^90^

The variables of saccadic EM were studied to differentiate cases (AD, MCI, or both) from HCs by keeping the target stimuli in the horizontal or vertical planes. Most studies positioned the target stimuli in the horizontal plane;^78^, ^80^, ^91^, ^92^, ^93^, ^95^, ^97^, ^99^, ^100^, ^101^, ^104^, ^106^, ^107^ however, some studies kept target stimuli in both vertical and horizontal planes.^74^, ^75^, ^76^, ^77^, ^89^, ^94^, ^96^, ^98^, ^105^ In the prosaccade and antisaccade paradigms, the majority of studies reported longer latency (the delay between the target appearing and the initiation of the saccadic EM) in the AD and MCI groups than in the controls.^74^, ^75^, ^76^, ^77^, ^79^, ^80^, ^91^, ^92^, ^93^, ^95^, ^97^, ^98^, ^99^, ^101^, ^105^ Some studies reported a non‐significant longer latency in the control group.^76^, ^94^, ^97^, ^100^, ^107^ This discrepancy may be attributed to variations in eye‐tracking technologies, sample sizes, and study populations (e.g., amnestic vs. non‐amnestic MCI,^107^ younger vs. older HCs, and ethnic groups^100^, ^101^). Also, different studies compared the amplitude, the distance travelled by the eye during saccade measured in degrees or minutes of arc. Gain, the ratio of the observed saccade amplitude divided by the anticipated saccade amplitude, was also tested. The majority of the studies reported lower values of prosaccade and antisaccade gain or amplitude in the AD and MCI groups than in the control group.^75^, ^76^, ^92^, ^93^, ^99^ In contrast, one study^78^ reported a non‐significantly smaller prosaccade amplitude in the control group than in AD. Among the saccadic variables, antisaccade error (prosaccade movements during antisaccade tasks) latency and antisaccade error rate were also found to be highly significant in distinguishing AD and MCI cases from HCs. In the previous studies, antisaccade error latency^74^, ^76^, ^77^, ^80^, ^99^, ^101^, ^105^ and antisaccade error rate^74^, ^75^, ^76^, ^77^, ^79^, ^80^, ^94^, ^99^, ^104^, ^105^, ^106^ in the AD and MCI groups were reported higher than in the control group.

A meta‐analysis^90^ of 27 EM studies with defined task conditions found that both prosaccade and antisaccade paradigms effectively differentiated patients with AD and MCI from HCs. Among them, antisaccade paradigms (*P* < 0.001) demonstrated greater sensitivity in distinguishing cases from controls compared to prosaccade paradigms (*P* < 0.01). Additionally, individuals with AD exhibited significantly longer latencies than those with MCI in the prosaccade task under both gap (standard mean difference [SMD] = 0.45) and overlap conditions (SMD = 0.26). A similar pattern was observed in the antisaccade task under the gap condition (SMD = 0.30). Also, participants with AD made significantly more error saccades than those with MCI (SMD = 0.53) in the antisaccade task.^90^ Although both prosaccade and antisaccade paradigms were shown to be susceptible to disease, aging, and ethnicity,^97^ results suggested that the antisaccade EM is more effective at differentiating cases (AD or MCI) from controls as well as MCI from AD. Larger sample sizes and longitudinal studies based on this paradigm are still needed to confirm its utility in clinical settings.

Despite the potential of EM biomarkers, it is still unclear how changes in EMs are associated with brain function or status. EM studies (see Table 1 and Table S4 in supporting information) are frequently used in research settings and are not routinely used in clinical practice to diagnose AD. There is a research gap in understanding EM patterns in individuals at the preclinical stage of the disease. Additional research is still required to develop standardized protocols, EM detection devices, and cut‐off values of EM parameters for AD identification and monitoring. Also, it is crucial to assess their reliability, sensitivity, and specificity in distinguishing AD from other conditions before using them for mass screening and longitudinal disease monitoring.

## 6 CHOROID

The choroid is a vascular layer between the retinal pigment epithelium (RPE) and the outer layer of the eye (sclera). The choroid is vital for maintaining blood flow to the retina and other ocular tissues^108^ Several studies have assessed Aβ deposits, blood flow, vessel density, thickness, and area changes in the choroid of individuals with AD‐related changes.^109^, ^110^, ^111^, ^112^ The enhanced depth imaging (EDI) technology in modern spectral‐domain optical coherence tomography (SD‐OCT) and recent swept‐source OCT (SS‐OCT) devices have enabled better observations of the choroid in the deeper layers of the eye.^113^

The results of using these choroid imaging methods have been mixed (see Table 1 and Table S5 in supporting information). SD‐OCT using EDI technology revealed decreased choroidal thickness (CT) in AD^109^, ^110^, ^114^, ^115^, ^116^, ^117^, ^118^, ^119^ and MCI participants^110^, ^115^ compared to controls. However, an early study in individuals with known brain Aβ status showed no significant difference in the choroidal thickness.^120^ A later, much larger study^121^ observed thinning of the choroid in AD participants (by an average median thickness of ≈ 35 µm), even while the retinal vessels were not yet affected, suggesting choroid thinning may be an early biomarker. The variation in results observed in this study compared to previous work^120^ might be due to the inclusion of mild AD participants as determined by the Clinical Dementia Rating (CDR), who may exhibit more pronounced choroidal pathology than individuals in preclinical stages of the disease. However, a more recent study,^122^ also with no indication of brain Aβ status, but comprised of different neurodegenerative cohorts, used SS‐OCT and demonstrated a significant increase in CT. This was adjusted for demographic factors, cardiovascular risk factors, and OCT image quality. The study was larger and included 301 HCs, 196 individuals with MCI due to AD (MCI‐AD), 112 with MCI due to cerebrovascular disease (MCI‐Va), 578 with AD, and 93 with vascular dementia (VaD). The increase in CT was observed particularly in the peripheral macular regions and in individuals with cerebrovascular‐related cognitive impairment (VaD and MCI‐Va) compared to HCs. Although MCI‐AD and AD also showed significant CT increases in certain regions, these changes were less pronounced than those observed in MCI‐Va and VaD, suggesting there may be some retinal vascular involvement in cerebrovascular disease. These results aligned with a previous *post mortem* analysis^123^ of 19 individuals (11 HC and 8 with AD), which reported thickening in superior‐temporal choroid within the macular regions in those with AD.

Aside from differences in cohorts, the discrepancies in findings may be attributed to variations in imaging modalities (SD‐OCT vs. SS‐OCT) and differences in the anatomical regions analyzed. Although EDI OCT provides improved visualization of deeper structures by positioning the regions of interest closer to the zero‐delay line, SS‐OCT offers greater penetration depth and improved choroidal visualization. Additionally, some studies assessed choroidal thickness within the Early Treatment Diabetic Retinopathy Study (ETDRS) regions,^110^, ^119^, ^121^ while others focused on areas peripheral to the ETDRS grid,^122^, ^123^ contributing to inconsistencies in reported outcomes.

Similarly, some studies have investigated alterations in the choroidal cross‐sectional area to evaluate the potential association with AD. Robbins et al.^112^ showed significantly larger total choroidal areas and luminal areas (the areas occupied by choroidal blood vessels in EDI‐OCT images^124^) in the AD and MCI groups compared to controls. They further demonstrated a significantly lower choroidal vascularity index (luminal area per unit choroidal area^125^) in the MCI group than in the control group. On the other hand, a longitudinal study analyzed choroidal structural parameters in people with carriers of higher genetic risk for AD (apolipoprotein E [*APOE*] ε4) and non‐carriers over 2 years.^126^ The result showed that baseline values of the total choroidal area, luminal area, and choroidal vascularity index did not significantly differ between groups. Furthermore, on these choroidal measures, there were neither statistically significant longitudinal differences between groups nor within groups. These results indicate choroidal changes are more apparent in the late stages of the disease. Some studies also reported that choroidal structural change is associated with other conditions such as aging, myopia, and uveitis,^127^, ^128^, ^129^ so further studies are required to examine the effects of these co‐morbidities and establish its performance as a biomarker or indicator of AD.

Moreover, studies conducted to analyze the choroidal vascular changes associated with AD and MCI reported mixed outcomes. In a previous study,^111^ blood flow measurements of the choroid, choriocapillaris perfusion density, and choroid perfusion density were compared in neuropsychological and biomarker‐proven AD, MCI, and control cohorts; however, no significant differences were found between the groups. In contrast, a subsequent study^130^ reported that choriocapillaris flow density was lower in early‐onset dementia participants than in controls and negatively correlated with both the length of the disease and the severity of the cognitive impairment measured by the Montreal Cognitive Assessment (MoCA).^130^ Another study showed increased choriocapillaris flow deficits in pathological CSF Aβ42/tau ratio participants compared to normal participants.^131^

Although choroidal changes hold potential as non‐invasive biomarkers for AD, inconsistencies in study methodologies, imaging modalities, population differences, and confounding systemic factors (e.g., age, sex, and cardiovascular disease) pose challenges for consistent results. Standardized imaging protocols, longitudinal studies on diverse cohorts, and multimodal biomarker (e.g., Aβ PET, CSF) integration are essential for validating choroidal parameter measurements for early disease detection and monitoring.

## 7 RETINA AND OPTIC NERVE

Light passes into the retina through different layers, which are: inner limiting membrane (ILM), retinal nerve fiber layer (RNFL), ganglion cell layer (GCL), inner plexiform layer (IPL), inner nuclear layer (INL), outer plexiform layer (OPL), outer nuclear layer (ONL), external limiting membrane (ELM), photoreceptive cell layer (PCL), and retinal pigment epithelium (RPE).^132^ These layers consist mostly of neurons and glial cells, while photoreceptors are only found in the PCL. The ganglion cells transmit visual signals to the brain via their non‐myelinated axons, which form the RNFL and converge at the optic disc.^133^, ^134^ These axons are myelinated by oligodendrocytes after exiting the eye, aligning the optic nerve more closely with the CNS than the peripheral nervous system. The retina, optic nerve, and CNS share several anatomical and physiological features.^15^, ^135^, ^136^ Consequently, investigating retinal changes offers a promising avenue for developing non‐invasive and differential diagnostic tools for AD.^137^ See Supplementary Section 3 and Figure S4 in supporting information for a comprehensive overview.

### 7.1 Retinal structural changes

**FIGURE 2:**
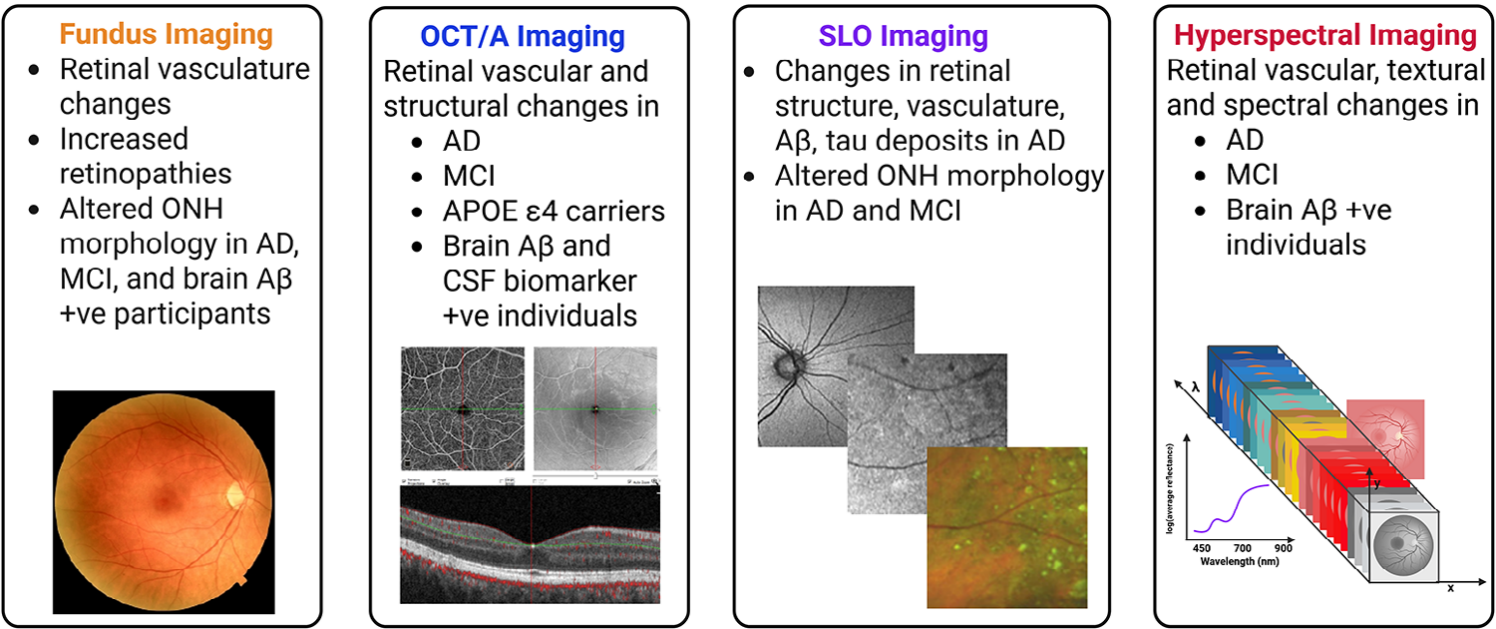
Current in vivo retinal imaging modalities and their findings on various cohorts. Color fundus and OCT imaging are the most established modalities, while SLO and hyperspectral imaging, which can provide higher resolution and spectral information, respectively, are still being developed. +ve, positive; Aβ, amyloid beta; AD, Alzheimer's disease; *APOE*, apolipoprotein E; CSF, cerebrospinal fluid; MCI, mild cognitive impairment; OCT/A, optical coherence tomography and optical coherence tomography angiography; ONH, optic nerve head; SLO, scanning laser ophthalmoscope.

In line with changes in the CNS in AD, research has also shown changes in the retina. These have been attributed to its common embryological origin with the CNS, with which it retains a close functional relationship throughout life.^15^ Extensive changes were observed in *post mortem* retinal tissue of AD cases and included thinning in the RNFL,^138^, ^139^ GCL, IPL, INL, and ONL^138^ layers; axonal loss in the optic nerve;^139^, ^140^ and neuronal degeneration in the GCL.^139^, ^141^ Consequently, retinal structural changes have drawn great interest as possible non‐invasive clinical indicators of AD using standardized approaches like OCT, OCT angiography (OCTA), and SLO imaging (see Figure 2, Table 1, and Table S6 in supporting information for a comprehensive overview). These *post mortem* studies have supported the results of in vivo retinal studies, suggesting that retinal structural imaging could be a viable biomarker for early AD detection.^68^, ^109^, ^114^, ^140^

OCT can produce a cross‐sectional image (B‐scan) of the retina by combining several one‐dimensional depth profiles (A‐scans). OCTA is an extension of the principles of OCT and detects the motion of red blood cells in blood vessels, enabling depth‐resolved volumetric data on the choroidal and retinal microcirculation without using contrast agents. The thickness of different retinal layers is typically identified and estimated through automatic segmentation and analysis of the OCT and OCTA images using advanced computer algorithms.^142^ Similarly, the SLO imaging system uses laser light sources to rapidly scan a confined region of the ocular fundus, with the reflected light captured by a detection system. This method enables the acquisition of high‐resolution images without the need for pharmacological pupil dilation. Additionally, using light backscattering, SLO can differentiate layers, measure the thickness and identify retinal blood vessels.^143^, ^144^

The majority of non‐invasive studies have focused on changes in RNFL thickness. This layer is primarily composed of projected axons from the ganglion cell layer prior to their fusion at the ONH.^132^ Its thickness is measured non‐invasively by measuring the distance between the external boundary of the RNFL and the inner limiting membrane using OCT and OCTA instruments. The RNFL thickness varies with distance from the center of the ONH and macula. Hence, most instruments compute RNFL thickness along a predefined circumference around the center of the ONH as peripapillary RNFL (pRNFL) with an average thickness of ≈ 105 µm and around the center of the macula as macular RNFL (mRNFL) with an average thickness of ≈ 35 µm.^145^

The literature describes thinning of the pRNFL,^17^, ^68^, ^140^, ^146^, ^147^, ^148^, ^149^, ^150^, ^151^, ^152^, ^153^, ^154^, ^155^, ^156^, ^157^, ^158^, ^159^, ^160^, ^161^, ^162^, ^163^, ^164^, ^165^, ^166^, ^167^, ^168^, ^169^, ^170^, ^171^, ^172^, ^173^, ^174^, ^175^, ^176^, ^177^, ^178^, ^179^, ^180^, ^181^, ^182^, ^183^ mRNFL,^145^, ^150^, ^152^, ^165^, ^184^, ^185^ GCL,^111^, ^152^, ^165^, ^183^, ^186^ ganglion cell layer and inner plexiform layer (GCL‐IPL),^149^, ^155^, ^162^, ^172^, ^173^, ^174^, ^185^, ^187^ RNFL‐GCL‐IPL (ganglion cell complex or GCC),^111^, ^114^, ^148^, ^149^, ^151^, ^170^, ^188^, ^189^ IPL,^111^, ^145^, ^152^ INL,^145^ and OPL^145^, ^165^, ^190^ in AD subjects compared to HCs. However, some studies could not detect any significant differences in pRNFL,^109^, ^126^, ^145^, ^184^, ^186^, ^187^, ^190^, ^191^, ^192^, ^193^, ^194^, ^195^, ^196^, ^197^ mRNFL,^111^, ^190^, ^195^, ^196^, ^197^, ^198^ GCL,^145^, ^190^, ^195^, ^196^, ^197^, ^198^ GCL‐IPL,^111^, ^126^, ^181^, ^182^, ^192^, ^199^ IPL,^165^, ^190^, ^196^, ^197^, ^198^ INL,^152^, ^165^, ^190^, ^195^, ^198^ ONL,^145^, ^190^, ^195^, ^198^ OPL,^152^, ^195^, ^198^ and RPE^145^, ^152^, ^165^, ^198^ layer thickness between AD subjects and HCs. These results suggest that alterations in AD‐related retinal layer thickness are subtle and less clear. Similarly, studies have found significant differences in macular thickness,^111^, ^149^, ^154^, ^165^, ^171^, ^173^, ^182^, ^185^ macular volume,^148^, ^154^, ^171^, ^173^, ^175^ foveal thickness,^111^, ^126^, ^149^, ^165^, ^182^, ^188^, ^200^ and foveal avascular zone (FAZ) area,^116^, ^131^, ^169^, ^170^, ^200^, ^201^ while others could not detect any significant differences in these measurements^120^, ^121^, ^126^, ^147^, ^158^, ^164^, ^168^, ^181^, ^187^, ^189^, ^192^, ^198^, ^202^, ^203^, ^204^, ^205^ between cases and controls. Studies also suggested a significant association of RNFL thinning with lower cognition,^149^, ^155^, ^178^, ^183^ increasing age,^155^, ^178^ and AD symptom duration^151^ in the individuals. Furthermore, studies on participants with AD‐related changes demonstrated retinal structural changes were associated with multimodal MRI changes.^206^, ^207^, ^208^ They suggest that retinal structural changes were associated with the loss of integrity of white matter fiber tracts in the visual pathway, decreased lateral geniculate nucleus volume, and functional metabolism of the primary visual cortex in AD patients.

Although most of the AD studies on structural changes in the retina revealed thinner retinal layers in cases compared to controls, some studies observed a significantly higher IPL^195^ thickness in the preclinical AD group and higher ONL thickness in the AD group^165^, ^185^ compared to controls. The inconsistency may result from the study cohorts having different stages of AD progression, study methods, participant diversity, and sample size. The majority of these studies are cross‐sectional and conducted on individuals with clinically diagnosed AD dementia, whereas others have only investigated MCI,^170^, ^182^, ^198^ both AD and MCI,^111^, ^171^, ^172^, ^173^, ^174^, ^175^, ^176^, ^177^, ^178^, ^179^, ^185^, ^187^, ^191^, ^192^, ^193^, ^199^, ^201^, ^203^, ^204^ Aβ or hyperphosphorylated tau positive,^120^, ^131^, ^181^, ^182^, ^186^, ^190^, ^195^, ^196^, ^197^, ^200^, ^205^ *APOE* ε4 gene carriers,^126^, ^145^, ^182^, ^198^ and cognitively impaired^189^ individuals as their subjects. Structural alterations in the retina have also been studied to differentiate people with AD and MCI, where significantly reduced pRNFL^175^, ^177^ was detected in AD participants compared to MCI. However, the majority of the other studies with MCI and AD subjects could not detect any differences between the groups. Consistent with these findings, a recent meta‐analysis^209^ including 53 cross‐sectional studies, which exhibited significant heterogeneity (*P* < 0.00001), assessed pRNFL thickness in 2441 AD and 2128 HC participants. The findings revealed a significant reduction in global pRNFL thickness in AD participants compared to HCs (≈ 4 µm reduction, SMD = −0.79, *P* < 0.00001). Additionally, AD participants exhibited decreased macular thickness (≈ 6 µm reduction, SMD = −0.44, *P* = 0.0003; number of AD participants = 633, number of HCs = 1376), macular volume (≈ 0.3 units reduction, SMD = −0.41, *P* = 0.02; number of AD participants = 533, number of HCs = 685), foveal thickness (≈ 6 µm reduction, SMD = −0.39, *P* < 0.0001, number of AD participants = 881, number of HCs = 977), GCC (≈ 3 µm reduction, SMD = −1.26, *P* = 0.01; number of AD participants = 310, number of HCs = 408), and larger FAZ area (≈ 0.07 units increase, SMD = 0.84, *P* = 0.01, number of AD participants = 226, number of HCs = 306) compared to controls. However, another meta‐analysis^210^ of 11 OCT studies involving individuals who were PET or CSF positive found limited evidence supporting a reduction in mean pRNFL thickness among cases compared to controls (total participants = 828, SMD = −0.20, *P* = 0.14). These findings are confirmed by another systematic review^211^ of 64 OCT studies, which demonstrated a consistent reduction in overall pRNFL and superior quadrant thickness, during the advanced stages of AD. However, these findings also indicate that pRNFL thickness may not be a reliable biomarker for early or mild‐stage AD detection. The variability in imaging methods and the extent of retinal changes among individuals at the preclinical, MCI, and advanced stages may contribute to these inconsistencies. Furthermore, ocular conditions such as gliosis and inflammation can lead to increased retinal thickness, while neurodegeneration may result in retinal atrophy and thinning depending on the disease status. These findings may suggest that the diagnostic efficacy of retinal structural changes to differentiate AD from MCI is limited. Due to these limitations, for a robust correlation of retinal data with AD disease status, the use of brain imaging information should ideally be used to determine the presence of disease pathology. Additionally, other eye conditions need to be ruled out to reduce the number of potential variables and confounding factors.

There were fewer longitudinal studies ranging from 12 to 54 months. These showed significant thinning in the pRNFL,^166^, ^167^, ^212^ mRNFL,^190^ GCC,^212^ IPL,^190^ INL,^213^ ONL,^190^ and OPL^190^ in cases over time compared to HCs. Also, mRNFL, GCL, IPL, OPL, and RPE layer thicknesses were significantly reduced, while no reduction in the INL, ELM, and Bruch's membrane layers was found before and after the diagnosis (over the 4 years) of AD.^214^ Among the layers that were significantly thinner, the pRNFL, mRNFL, GCL, and IPL are primarily composed of retinal ganglion cells, projection neurons that transmit visual information to the brain. These results from longitudinal studies support ganglion cell degeneration in AD. Other longitudinal studies have suggested that RNFL thinning is associated with the volumetric decrease of the cingulate cortex,^215^ lower cognitive performance in childhood and adulthood,^216^ higher risk of conversion to AD dementia from MCI, based on clinical and neuropsychological evaluations^173^ and a decrease in processing speed.^216^ A longitudinal decrease in GCL‐IPL thickness was also found to be associated with the conversion to AD dementia from MCI.^173^ Additionally, population‐based longitudinal studies found an association between a thinner RNFL and an increased probability of developing dementia, including AD^162^ and lower cognitive performance of the participants at baseline as well as after 3 years of assessments.^217^

Similar to the cross‐sectional studies on structural changes associated with AD, the results of longitudinal studies do not completely agree. One study^190^ detected a significantly higher OPL thickness in the temporal quadrant and no significant difference in pRNFL, GCL, and INL retinal layers of preclinical AD over 27 months than in controls. Similarly, a study using OCT did not observe any significant changes in pRNFL, mRNFL, GCL, and IPL thickness between preclinical AD and HC groups over 22 months.^196^ Furthermore, studies comparing *APOE* ε4 carriers versus non‐carriers could not observe any significant difference in pRFNL, GCL‐IPL,^126^ mRFNL, GCL, IPL, OPL, ONL, and RPE^213^ layers thickness over 2 years. Therefore, longitudinal studies with larger well‐characterized cohorts are needed to confidently establish whether the changes in the retinal layer are associated with AD.

Retinal structural atrophy is not specific to AD and can also be a sign of normal aging and other neurodegenerative conditions like glaucoma^68^ and PD.^52^ A study^218^ reported a significant reduction in retinal RNFL thickness (*P* < 0.001) in individuals with AD, LBD, and PD compared to HCs, with each group comprising 10 participants. However, no significant differences in RNFL thickness were observed among these neurodegenerative conditions, limiting its utility in differentiating among these types of dementia. Retinal changes can also be influenced by other factors, including vascular issues, inflammation, and systemic diseases like hypertension and diabetes mellitus,^219^, ^220^ which may affect prediction accuracy. Similarly, the measurement of retinal structural changes may vary depending on the retinal imaging technique and tools used. This variability prevents it from being used as a reliable biomarker. Although studies have observed an association between retinal structural atrophy and AD, it is still unclear if it is a direct or indirect effect of the disease. As a result of factors like genetics and general health, each person has a unique baseline level of retinal features. Due to this diversity, it may be challenging to set universal cut‐offs for distinguishing pathological atrophy.

In conclusion, retinal structural atrophy could be a potential biomarker for AD. However, several confounding issues must be resolved before it can be extensively used for clinical diagnosis and monitoring. Further larger scale studies to account for the diversity and distinguish different pathologies are required to establish its usefulness and overcome these practical challenges.

### 7.2 Retinal vascular changes

Similarities between the retina and CNS extend to the retinal microvascular system. Retinal microvascular changes are reflected in numerous cerebrovascular and neurodegenerative disorders, such as hypertension, diabetes mellitus, AD, and PD. Although vascular changes are frequently observed in the AD brain,^221^, ^222^ there are currently no easily accessible non‐invasive techniques to visualize the cerebral microvasculature. As a result, non‐invasive retinal imaging techniques such as OCTA, SLO, fluorescein angiography, fundus imaging, and hyperspectral imaging have gained significant attention (see Figure 2, Table 1, and Table S7 in supporting information).

Studies have analyzed microvascular density near the RNFL and GCL, known as the superficial capillary plexus (SCP) and deeper inner retinal layers, known as the deep capillary plexus (DCP) and total vascular density (VD). OCTA imaging studies have shown a significant reduction in the retinal microvascular density around SCP,^131^, ^168^, ^170^, ^187^, ^189^, ^198^, ^199^, ^202^, ^203^, ^204^, ^223^, ^224^ around DCP,^168^, ^169^, ^170^, ^189^, ^198^, ^199^, ^201^, ^203^, ^223^, ^225^ and total VD of the retina^116^, ^199^, ^205^, ^226^ in cases compared to controls. However, some studies reported no difference between the retinal microvascular density around SCP,^169^, ^198^ DCP,^131^, ^198^, ^202^, ^204^ and retinal total VD^120^, ^126^ of subjects and controls. Additionally, one study^227^ showed an apparent increase of vascular density in certain retina sectors in participants with a genetic risk factor for AD. The majority of the vascular alterations are predominantly evident in the later stages of the disease. Notably, inconsistent results were observed, which may be attributed to variations in study methodologies, cohort characteristics, and sample sizes.

Consistent with these results, a meta‐analysis^209^ of 73 studies with considerable heterogeneity in OCTA scanning parameters and image analysis techniques across studies, identified significant alterations in vascular parameters between AD participants and HCs. This study included subjects at various disease stages identified by established diagnostic criteria. The findings indicated that AD participants had significantly lower SCP microvascular density (SMD = −0.42, *P* = 0.001, number of AD participants = 318, number of HCs = 409) and reduced DCP microvascular density (SMD = −0.46, *P* = 0.001, number of AD participants = 211, number of HCs = 219) compared to HCs.

A recent multi‐center study^225^ used a deep learning classification approach on 5751 OCTA images of retinal microvasculature and choriocapillaris from 1671 participants, demonstrating high diagnostic performance in detecting early‐onset AD (EOAD) and MCI compared to HC. The model achieved superior accuracy for EOAD (AUC = 0.94 for internal data; AUC = 0.90 for external data) and MCI (AUC = 0.86 for internal data; AUC = 0.80 for external data). More pronounced OCTA feature differences were observed in EOAD than in MCI relative to HC. This study suggested that EOAD, despite occurring in younger individuals, exhibited severe pathology that impacts retinal vasculature. Furthermore, MCI, as an early‐stage condition with milder symptoms, caused less pronounced changes. This implies that dementia‐related alterations in retinal structure become more evident with disease severity.

Similarly, retinal microvascular anomalies have been observed to be associated with cognition,^116^, ^224^ structural brain changes as measured by MRI,^228^ retinal layer thickness,^198^, ^199^, ^204^ cognitive ability, and AD severity.^168^, ^189^ Moreover, retinal arterial dilation was found to correlate with CSF Aβ levels in AD and MCI subjects,^111^ whereas the retinal venous and arterial pulsation amplitudes were found to correlate with neocortical Aβ scores negatively and positively, respectively.^186^ Multiple studies have suggested a wide range of retinal vascular abnormalities, including decreased blood flow,^146^, ^191^ reduced perfusion density,^131^, ^187^, ^224^ altered microvascular network,^120^, ^131^, ^203^, ^223^, ^229^, ^230^, ^231^, ^232^, ^233^ increased retinal oxygen saturation in arterioles and venules,^234^ venule diameter changes,^146^, ^191^, ^230^, ^231^, ^232^, ^235^ and increased vascular tortuosity^230^, ^231^, ^236^ in AD cases compared to controls. Conversely, some studies reported reduced vascular tortuosity^232^, ^233^ and a higher retinal artery diameter in the vicinity of the optic disc^233^, ^236^ in the AD retina than in the controls. However, some studies could not demonstrate any significant differences in retinal perfusion density,^111^, ^126^, ^203^ decreased blood flow,^116^ artery diameter,^229^, ^235^ venular diameter,^229^ and altered vascular network^231^, ^233^ between cases and controls. Researchers have hypothesized that vascular measures such as caliber vary with the cardiac cycle and can be altered in static retinal images. So, vascular screening methods will indeed be effective if vascular parameters can account for pulse‐driven fluctuations.^60^ Other factors for these inconsistent outcomes include differences in the study instrumentation, sample size, and subjects. For example, the majority of the studies on retinal vascular changes in AD are cross‐sectional studies including individuals with AD dementia, while others have investigated cohorts with MCI,^170^, ^198^ both AD and MCI,^111^, ^187^, ^191^, ^199^, ^201^, ^203^, ^204^, ^223^, ^224^, ^230^ neocortical Aβ or hyperphosphorylated tau positive,^120^, ^131^, ^200^, ^205^, ^226^, ^236^ *APOE* ε4 gene carrier,^198^, ^226^ and cognitively impaired^189^, ^225^, ^229^ individuals as subjects. Among the studies with AD and MCI subjects, only a few were able to detect significant differences in vascular parameters.^187^, ^201^

As discussed above, most studies on retinal vascular parameters were performed cross‐sectionally in AD cohorts and had small sample sizes. This may partially explain the inconsistent findings. A prospective population‐based study with a mean follow‐up of 11.6 years involving 5553 participants reported an association between venular caliber and an increased risk of vascular dementia, but not AD.^237^ Another 2 year longitudinal study^212^ found that MCI participants who did not later develop dementia demonstrated significant VD reduction in the DCP region. Similarly, they found a higher VD reduction in the SCP, radial peripapillary capillary (RPC) plexus, and increased FAZ area in the whole MCI group, including both converters and non‐converters to dementia. These results support the assumption that vascular alterations may be involved in the onset and progression of AD. However, a 2 year longitudinal study^126^ comparing *APOE* ε4 carriers and non‐carriers showed no significant differences in perfusion density, VD, and FAZ area. This inconsistency suggests that more research is needed for ocular vasculature to be used as a viable biomarker of AD.

*Post mortem* studies involving AD participants and transgenic mouse models have demonstrated impairment of both the blood–brain barrier^238^ and the blood–retinal barrier.^239^, ^240^ Observed changes include pericyte loss,^239^ Aβ deposition,^239^ and retinal capillary degeneration^240^ in mice, as well as reduced vascular platelet‐derived growth factor receptor‐β expression in individuals with AD and MCI (see Supplementary Section 4 in supporting information for a summary). Interestingly, vascular amyloidosis and other microvascular alterations have been observed in the retinas of MCI and AD individuals. Additionally, there was a strong correlation between retinal vascular abnormalities and the severity of cerebral amyloid angiopathy (CAA).^239^, ^241^, ^242^ However, the question remains whether retinal vascular Aβ deposition is a cause or an effect of brain pathology. Also, translating these histological findings into a practical clinical screening method requires the development of an effective non‐invasive imaging technique capable of detecting AD‐related molecular changes (e.g., Aβ aggregation) in the retina.

Overall, studies have found retinal vascular abnormalities in both human and transgenic AD mouse models. However, vascular changes can occur due to venous constriction, vascular leakage, and some cerebrovascular and neurodegenerative diseases, such as PD, diabetes, hypertension, and normal aging.^68^, ^169^, ^194^, ^243^, ^244^, ^245^ The degree of impairment and nature of retinal vascular abnormalities in people with AD may vary widely. This variation may be affected by genetic factors, disease stages, co‐occurring medical conditions, and other circumstances. Although retinal vascular alterations seem to have promise as a potential biomarker for AD, recognizing the AD‐related changes from the non‐AD alterations presents challenges and limitations that must be overcome before they can be used clinically for AD diagnosis, prognosis, or monitoring. Longitudinal studies, better cohort characterization, standardized research designs, interdisciplinary approaches, and collaboration are crucial to address these challenges and establish the clinical significance of retinal vascular changes in AD.

### 7.3 Optic nerve head changes

The optic disc or ONH is a feature of the posterior ocular fundus where retinal ganglion cell axons exit the eye, and it is a connection point for the ocular blood supply. Researchers have investigated the ONH structure non‐invasively using several techniques, including OCT, OCTA, fundus photography, SLO, and Doppler imaging as potential AD detection methods.^17^, ^121^, ^205^, ^246^, ^247^ Earlier studies using fundus photography and SLO imaging demonstrated that AD participants had a significantly higher cup‐to‐disc ratio, larger cup volume, decreased disc rim area, and a non‐significantly higher pallor area‐to‐disc area ratio compared to HCs.^180^, ^246^ These ONH parameters were also shown to correlate with Alzheimer's Disease Assessment Scale (ADAS) scores and disease duration.^246^ However, later studies using SLO and OCT could not detect any difference in ONH disc area, rim area, cup/disc area ratio, and cup volume between AD and HC participants.^68^, ^169^, ^194^ Variations in imaging equipment, study cohorts (e.g., clinical^68^, ^169^, ^180^, ^194^, ^246^, ^247^ vs. biomarker‐proven AD participants^205^), disease duration, severity, and most importantly, participant age, likely contribute to inconsistent findings. Similarly, optic disc color analysis^17^ using Laguna ONhE (optic nerve head hemoglobin) software showed that papillary paleness related to axonal loss and blood perfusion changes were higher in mild or moderate AD participants. However, a later study using the same software^121^ did not find significant differences in hemoglobin measurements in the ONH between individuals with mild AD (based on the CDR) and controls.

More recently, an OCTA imaging study showed vessel density around the ONH (3–6 mm ring) was significantly higher in PET brain Aβ‐positive participants compared to Aβ‐negative participants.^205^ Another OCTA imaging study showed lower vascular density in the AD (confirmed by MRI and cognition test) group at the nerve head level (from the inner limiting membrane to 150 µm below) and radial peripapillary capillary level (from the internal limiting membrane to the RNFL) than in the control group.^247^ These findings indicate that the ONH can be altered by AD pathogenesis; however, some ONH parameter changes are also observed in aging and other ocular conditions, such as glaucoma.^248^ Also, a few studies have investigated ONH changes in AD with considerable variability in the study cohorts (see Table 1 and Table S8 in supporting information for a comprehensive overview). Most prior research has focused on cognitively diagnosed AD cases, which may not accurately reflect the underlying biochemical changes occurring in the brain and retina. So, further study is needed to establish whether ONH changes can be used as reliable AD indicators.

### 7.4 Pathological changes in the retina

**FIGURE 3:**
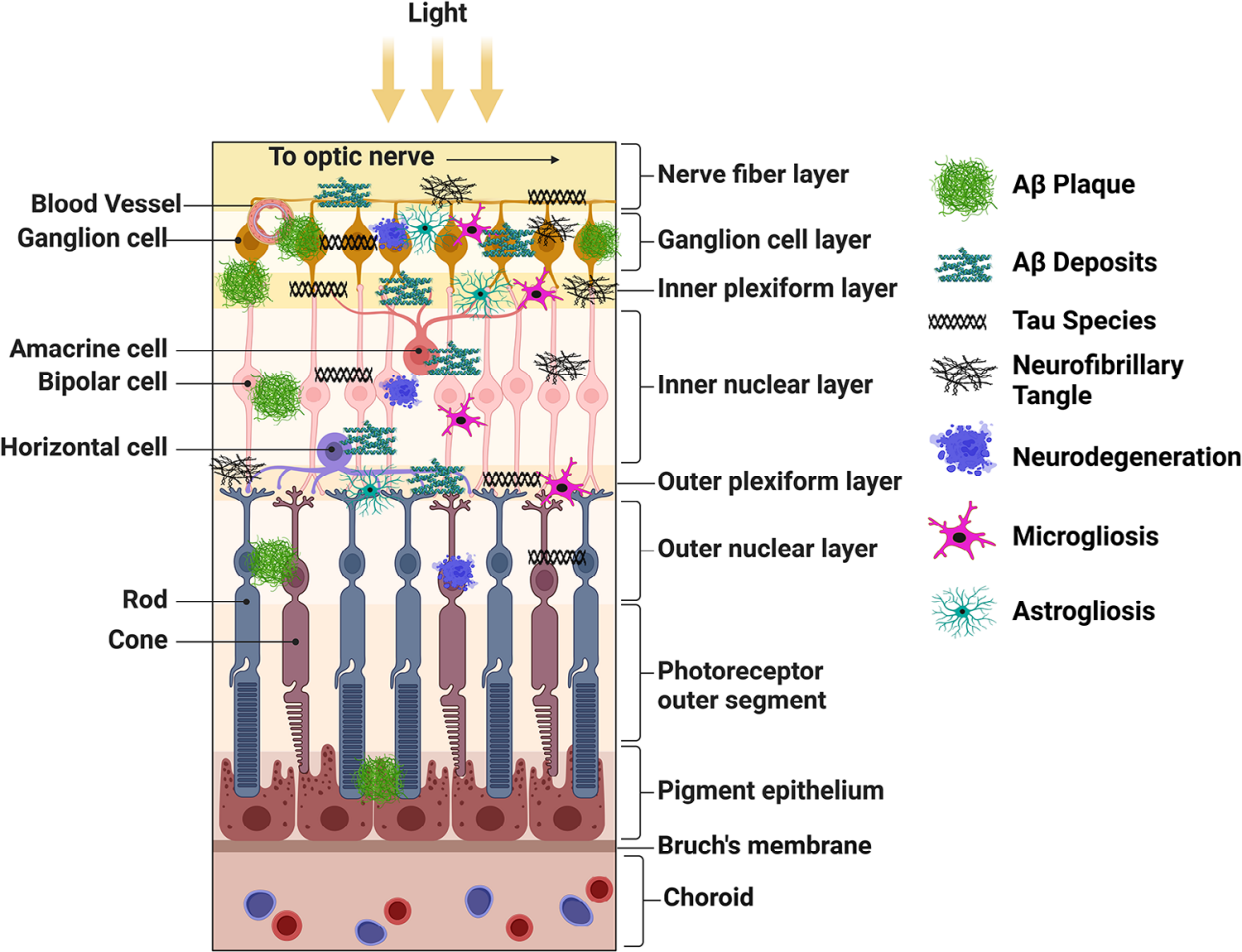
Observed retinal pathologies in AD and MCI patients at different layers of the retina. The figure presents a schematic representation of pathological alterations across the various retinal cell layers associated with AD. Aβ plaques and fibrillar Aβ deposits are identified throughout the retinal layers, including the retinal RNFL,^251^ GCL,^140^, ^241^, ^250^, ^251^, ^253^, ^254^ IPL,^241^, ^250^, ^251^, ^253^ INL,^140^, ^241^, ^250^, ^251^, ^253^ OPL,^250^ ONL,^241^ ELM,^241^ and RPE.^241^ Tau deposition, in the form of different isoforms and neurofibrillary tangles, is frequently observed in the RNFL,^256^, ^257^ GCL,^241^, ^255^, ^256^, ^257^ IPL,^253^, ^254^, ^255^, ^256^, ^257^, ^258^, ^259^ INL,^241^, ^255^, ^256^, ^258^, ^259^ OPL,^253^, ^254^, ^255^, ^257^, ^258^, ^259^ and ONL.^256^ Neurodegeneration is primarily detected deeper in the retina, particularly in the GCL,^241^ INL,^241^ and ONL.^241^ Additionally, inflammatory responses such as microgliosis have been reported in the GCL,^252^ IPL,^252^ INL,^250^ and OPL,^250^, ^252^ whereas astrogliosis is typically observed in the GCL,^252^ IPL,^250^ and OPL.^250^ Aβ, amyloid beta; AD, Alzheimer's disease; ELM, external limiting membrane; GCL, ganglion cell layer; INL, inner nuclear layer; IPL, inner plexiform layer; MCI, mild cognitive impairment; ONL, outer nuclear layer; OPL, outer plexiform layer; RNFL, retinal nerve fiber layer; RPE, retinal pigment epithelium.

AD is characterized by the formation of extracellular Aβ plaques and intracellular neurofibrillary tangles composed of hyperphosphorylated tau.^4^ Koronyo‐Hamaoui et al.^249^ was the first to report the presence of Aβ plaques in the *post mortem* retina of individuals with confirmed and probable or possible AD. Subsequent *post mortem* investigations have identified various forms of retinal Aβ deposition, including oligomeric or fibrillar,^239^, ^241^, ^250^, ^251^, ^252^, ^253^ and plaque‐like aggregates (Figure 3).^140^, ^241^, ^249^, ^252^, ^254^ Additionally, some studies^239^, ^241^ have reported perivascular Aβ accumulation along retinal blood vessels, with some cases showing substantial deposits. However, other studies^52^, ^54^, ^253^, ^255^ have not found significant differences in retinal APP or Aβ pathology between AD and control retinal tissues. These inconsistencies may be due to methodological variations, such as differences in tissue processing techniques and antibody sensitivity,^52^, ^54^, ^253^, ^255^ small study size,^52^, ^253^, ^255^ or the use of fewer tissues in the cross‐sections than in whole‐mount sample preparations^52^ and lower concentrations of Aβ‐binding antibodies.

In addition to Aβ‐related retinal pathology, several studies have identified various tau species, including hyperphosphorylated tau (p‐tau), within different retinal layers of the AD retina (Figure 3). Although a few investigations have reported the presence of neurofibrillary tangles,^254^, ^256^, ^257^ most *post mortem* human retinal studies have primarily detected different tau species, including diffuse p‐tau deposits,^253^, ^255^, ^256^, ^258^, ^259^ with limited evidence for fibrillar or more mature tau forms. Conversely, some previous studies^52^, ^54^ failed to observe any tau pathology in AD retinas, which may be attributed to differences in sample size, use of non‐optimal techniques, limited anatomical regions analysis, or the use of varying tau isoform‐specific markers. Further research is needed to clarify the characteristics of retinal tauopathy in AD, including its spatial distribution, progression from immature to mature forms, potential for propagation, and its role in retinal inflammation and neurodegeneration.

These findings on Aβ pathology and tauopathy highlight the necessity of using standardized experimental protocols to reliably detect AD‐related Aβ and tau pathology in the human retina. To address methodological variability and enhance the reproducibility of results, collaborative efforts that integrate diverse research approaches are essential. See Supplementary Section 4 in supporting information for a detailed overview of observed retinal amyloidopathy and tauopathy in both human and mouse models.

In addition to amyloidopathy and tauopathy, studies have consistently reported neurodegeneration, APP presence, pericyte loss, and gliosis in the human and AD mouse retina.^239^, ^250^, ^252^, ^254^, ^260^, ^261^ However, these are also associated with ocular pathologies, such as PD, age‐related macular degeneration (AMD), and glaucoma.^262^, ^263^, ^264^, ^265^, ^266^ Therefore, biomarkers that can differentiate between age‐related and disease‐related changes in the retina would be ideal, so that retinal imaging might be used as part of an in vivo AD biomarker assessment panel.

In vivo imaging has been previously used for detecting pathological changes in the AD retina. The first in vivo fluorescence retinal imaging study^249^ used intravenous injection of curcumin for the detection of Aβ plaques in the AD mouse model retina. In their follow‐up studies,^241^, ^267^, ^268^ human volunteers were administered curcumin orally for several days prior to imaging their retinas with an SLO. The AD participants were found to have elevated numbers of retinal Aβ deposits highlighted with curcumin‐based fluorescence.^241^ This was also observed in cognitively impaired participants compared to HCs.^267^, ^268^ However, it is critical to note that curcumin is not an antibody, so its specificity for Aβ might be limited, indicating the possibility of false positives. Other SLO studies have also suggested increased Aβ depositions,^269^ increased accumulation of hard drusen (AMD‐like pathologies),^235^ and higher reflective granular membranes (potentially manifestations of inner retinal gliosis)^193^ in AD and MCI participants. The greater presence of hard drusen in AD participants versus HCs, even after adjusting for age (*P* = 0.001), suggests a potential link between AMD and AD.^235^ Nonetheless, further research is required to elucidate the underlying mechanisms and validate these associations. Similarly, fibrillar tau has also been detected in vivo by fluorescence in the mouse retina, using intravenously administered fluorophore and subsequent observation with SLO.^255^ Their longitudinal observation also confirmed an increase in the proportion of ganglion cells containing fibrillar tau aggregates. Although still experimental, these fluorescence‐based retinal imaging techniques are minimally invasive. They show potential for large‐scale screening of at‐risk populations and monitoring clinical responses in AD.

Recent studies have used non‐invasive in vivo retinal hyperspectral imaging (rHSI) on AD participants and mouse models to evaluate the retinal Aβ load.^261^, ^270^, ^271^, ^272^, ^273^, ^274^ This technique is an evolving imaging modality based on conventional spectroscopy and computer imaging techniques. It collects several monochrome images over a range of specific wavelengths, rather than the standard single‐color fundus image.^275^ Early studies in mice^273^ used topical endoscopic fundus imaging to capture images in the 400 to 700 nm wavelength range. They showed that the spectra from soluble Aβ aggregates in the transgenic mouse retina were similar to those earlier reported in *post mortem* retinas from human AD cases. In a subsequent study, human retinas were analyzed in the 400 to 1000 nm spectrum, which revealed that the rHSI signature has a higher sensitivity in the early stages of AD during which cognitive decline was mild (MMSE score 22–26 out of 30).^271^ Another in vivo rHSI study of 5xFAD mice in the wavelength range of 320 to 680 nm suggested that an increase in reflectance at shorter wavelengths coincides with the increased level of Aβ oligomers in the retina.^274^ Similarly, the reflectance at longer wavelengths (> 550 nm) was observed to increase with the advancing age of mice when Aβ plaque deposition also increased in the retina.

Modern imaging techniques produce increasing volumes of data which are impossible for human interpretation. Artificial intelligence (AI) is now increasingly being used for the analysis of large datasets. Integration of AI with bioinformatics and computer science has accelerated biological discoveries, including AD research, and has the potential to revolutionize both diagnosis and treatment.^276^ Machine learning (ML) is a subdivision of AI that analyzes the features of the data and creates statistical algorithms to generalize unfamiliar data. It enables the identification of patterns in the data and makes predictions based on those patterns. AI has been used to extract novel features or to analyze previously recognized biomarkers using various imaging modalities.

In one study,^270^ the retinal reflectance obtained from hyperspectral imaging together with RNFL thickness from OCT was used to train an ML approach to discriminate AD participants from HCs. It demonstrated that the combination of rHSI and OCT can improve the accuracy of an rHSI‐based model for AD detection. Another in vivo rHSI study on the AD mouse model revealed that hyperspectral scores (HSs) for *App^NL^* ^‐^ *^G^* ^‐^ *^F^* mice due to retinal Aβ burden were considerably higher than those for age‐matched wild‐type mice. The results of this study suggest that rHSI can serve as a potential non‐invasive biomarker for AD diagnosis and monitoring.^261^ Recent studies^236^, ^272^, ^277^ have used a similar hyperspectral camera to capture retinal images in the wavelength range of 450 to 905 nm in individuals with elevated brain Aβ, as confirmed by PET imaging, alongside age‐matched controls. One study^272^ achieved an AUC of 0.87 using an ML model based on hyperspectral reflectance scores, while another study^236^ reported a sensitivity of 0.82 by incorporating vascular and textural features. In our analysis,^277^ ML models using retinal reflectance features yielded AUCs ranging from 0.72 to 0.90, depending on the features taken from retinal regions. Variability in classification performance across studies may be attributed to differences in sample size, analytic retinal features, and ML models (e.g., DROP‐D,^272^ SVM,^236^ and Integrated PCA‐FLD^277^).

Similarly, retinal tau is also drawing attention as a potential AD marker. An ex vivo hyperspectral imaging study^257^ on retinal cross‐sections has shown that both Aβ and p‐tau in the AD retina can also be detected through distinct rHSI spectra with no requirement for labeling. In general, these studies demonstrate that as a non‐invasive, label‐free technique, rHSI shows great promise for the widespread screening of AD (see Table 1 and Table S9 in supporting information for a summary). However, the studies to validate the concept are limited in size and number, as this technology is not yet widely available.[Table alz70476-tbl-0002]

For more established retina imaging technologies, various open data–sharing initiatives have begun collecting and disseminating clinical and molecular data from diverse cohorts. Studies have used ML models, using openly accessible two‐dimensional images obtained through OCT, SLO, and fundus cameras, to develop classification algorithms.^278^, ^279^, ^280^, ^281^, ^282^ One such open‐access database and repository is the UK Biobank dataset. It is a comprehensive public archive that contains extensive phenotypic, genetic, and imaging data, including color fundus images and OCT scans. One of the aforementioned studies used this large database and developed a deep learning‐based method for AD diagnosis, using 85,711 retinal fundus images from 111 AD participants and 111 HCs.^278^ The model demonstrated strong classification performance, achieving an AUC of 0.929, a sensitivity of 0.837, and a specificity of 0.891 on validation data. Additionally, multiple studies have used AI methods to develop classification models;^279^, ^280^, ^281^, ^282^, ^283^, ^284^ however, these approaches have not yet reached the practical and technological readiness for implementation in clinical settings^285^ (see Table S10 in supporting information). In this context, it will be essential to develop a mechanistic understanding of retinal changes throughout the AD spectrum before applying machine learning algorithms to detect, diagnose, or monitor AD‐related changes to ensure the validity of the respective techniques.[Table alz70476-tbl-0003]

Overall, these promising findings from multiple studies indicated the presence of Aβ and tau pathologies in the retina of both human and transgenic mouse AD models (Figure 3). Immunohistochemical staining and advanced imaging techniques such as OCT, SLO, and rHSI have been extensively used to demonstrate the association of ocular changes that reflect AD pathology. Recent studies using non‐invasive in vivo rHSI techniques have also shown promising results in distinguishing AD subjects from controls. This reinforces the idea that pathological changes in the retina closely reflect brain neuropathology. This indicates that retinal changes could be valuable biomarkers for non‐invasive AD detection. However, translating these observed pathological changes into reliable, cost‐effective tools for clinical settings presents significant challenges.

## 8 CHALLENGES AND FORESIGHT FOR TRANSLATION OF OCULAR ALTERATIONS AS A DIAGNOSTIC TOOL FOR AD

Many ocular changes in AD might not solely be associated with AD but also with age, other neurological or ocular conditions, or other health issues. This lack of specificity can result in reduced diagnostic accuracy. Also, the severity of ocular changes may vary among AD individuals, making it difficult to establish consistent diagnostic criteria and thresholds.^43^, ^127^, ^286^ Eye imaging may also be limited by conditions, such as cataracts, that obstruct the view of the retina. Furthermore, while some ocular changes may be indicative of advanced stages of AD, research is still needed to determine whether they reflect the brain pathology at preclinical stages or are due to other forms of dementia. Table 2 illustrates the challenges in translating ocular alterations into a reliable biomarker for AD.

**TABLE 2 alz70476-tbl-0002:** Challenges in translating ocular changes into reliable biomarkers for AD.

Challenge	Explanation
Lack of a standardized histopathological method for analyzing AD‐associated retinal pathology	Variability in pathological techniques, staining protocols, and sample sizes leads to inconsistencies in findings. Differences in staining methods (e.g., Bennhold's^46^ vs. Puchtler's^51^ techniques) and immunostaining antibodies further complicate comparability. Additionally, *post mortem* interval, tissue fixation, preservation methods, and whole‐mount preparations introduce discrepancies. The unclear relationship between Aβ/p‐tau pathology in the retina and brain raises critical questions regarding disease progression and protein propagation between the retina and brain.^258^, ^287^ Harmonization of histopathological methods is necessary to facilitate the translation of ex vivo results into in vivo diagnostic applications.
Lack of standardized methods to collect data and process images across in vivo imaging techniques	Retinal imaging studies lack a unified framework for data acquisition and analysis, impeding cross‐study comparability. Ocular imaging needs more consistent protocols and cooperation between research and clinical groups similar to diabetic retinopathy^220^ and Aβ PET measured in the Centiloid scale.^10^ Variability in imaging instruments across manufacturers, frequent software updates, and resolution modifications introduce subtle but significant differences in data collection and interpretation. The absence of standardized methodologies limits the development of a widely accessible reference database, similar to large‐scale studies such as Alzheimer's Disease Neuroimaging Initiative (ADNI)^9^ for AD biomarker research.
Inconsistent inclusion and exclusion criteria for participants in AD biomarker studies	Variability in study objectives, diagnostic frameworks, and disease progression stages results in inconsistent participant inclusion/exclusion criteria. Many studies exclude non‐AD neurodegenerative conditions (e.g., vascular dementia, frontotemporal dementia) and co‐existing ocular conditions (e.g., glaucoma, cataracts, diabetic retinopathy),^126^, ^236^, ^277^ while some lack clear criteria for exclusion of these conditions. The severity of AD among study participants often varies, encompassing individuals with mild, moderate, and severe stages of the condition. Additionally, inconsistencies in cognitive assessments (MMSE, MOCA)^115^, ^130^ and biomarker measurements (PET brain Aβ, CSF markers)^58^, ^181^, ^202^ reduce the comparability of findings.
Lack of consistency in effect and sample sizes	Small and inconsistent sample sizes reduce the statistical power and reliability of findings. Effect sizes (Cohen d, SMD, Pearson *r*, odds ratios) are often overlooked, leading to findings that may lack practical significance. Histological studies typically include 5 to 75 samples per group, while non‐invasive studies using OCT/OCTA include 5 to 100 participants per group. In contrast, large population‐based studies analyzing RNFL changes^162^, ^217^ and fundus imaging^279^, ^280^ have followed thousands of participants over multiple years. These discrepancies hinder result interpretation and generalizability.
Limited longitudinal studies	Most retinal biomarker studies rely on cross‐sectional designs, which capture changes at a single time point but fail to provide insights into disease progression. The lack of long‐term follow‐ups makes it difficult to determine whether retinal alterations are early biomarkers of AD or secondary effects of neurodegeneration. Many longitudinal studies have small cohorts and short follow‐up durations, limiting statistical power and generalizability. The absence of well‐validated longitudinal data restricts the integration of retinal biomarkers into clinical practice.
Limited cohorts and co‐pathology groups	The lack of diverse and well‐characterized participant groups limits the applicability of retinal imaging as a diagnostic and monitoring tool. AD often coexists with other neurodegenerative conditions, such as vascular dementia and Parkinson's disease. Many studies have small sample sizes with limited diversity in age, genetic risk factors (e.g., *APOE* ε4 status), and comorbidities,^198^, ^226^, ^227^ making it difficult to generalize findings. The absence of studies addressing co‐pathologies complicates the determination of whether observed ocular changes are AD specific or influenced by other conditions.
Device availability, costs, and operator expertise	Although OCT and fundus imaging are common in ophthalmology, specialized modalities such as hyperspectral imaging and advanced CCM are less accessible. Many techniques (e.g., pupillometry, CCM, hyperspectral imaging) are operator dependent or require significant training, raising concerns about large‐scale implementation. The high cost and limited availability of advanced imaging devices pose additional barriers to widespread adoption in research and clinical settings.

Abbreviations: Aβ, amyloid beta; AD, Alzheimer's disease; *APOE*, apolipoprotein E; CCM, corneal confocal microscopy; CSF, cerebrospinal fluid; MMSE, Mini‐Mental State Examination; MoCA, Montreal Cognitive Assessment; OCT, optical coherence tomography; OCTA, optical coherence tomography angiography; PET, positron emission tomography; p‐tau, hyperphosphorylated tau; RNFL, retinal nerve fiber layer; SMD, standard mean difference.

As some of the least invasive measures, pupillary response and corneal imaging have shown promising results. However, the limited numbers of human clinical studies indicate that reproducible findings in larger cohorts and longitudinal studies are still needed. EM studies have also demonstrated potential, particularly at the clinical stages of AD. These methods still require more sensitive instruments with advanced computational techniques and should be validated in preclinical AD stages to test their sensitivity.

Among ocular biomarkers investigated, OCT, OCTA, SLO, and fundus imaging of retinal structural and vascular changes have been the most extensively studied. Meta‐analyses further indicate that these modalities yielded the highest number of consistent findings with large effect sizes at clinical AD stages.^90^, ^209^, ^210^, ^211^ This is made possible as they have been more widely adopted than other ocular imaging technologies. Extensive studies on various cohorts using these modalities could serve as a strong foundation for developing retinal imaging biomarkers for AD in clinical applications.^287^

Studies using rHSI, particularly those integrating AI, have demonstrated promise in detecting retinal changes at preclinical stages. Because this is a relatively new area of research, there are still methodological and study design differences, cohort inclusion criteria, region‐of‐interest selection, image segmentation, and analysis methods. Also, rHSI technology is still developing, and many technological challenges remain to be resolved.

The studies reviewed had diverse approaches to study cohort construction, some of which led to conflicting results. Ideally, a thorough characterization of cohort members would establish a robust foundation for a reliable investigation into the effectiveness of ocular diagnosis approaches to AD. However, building cohorts that have been defined by established AD biomarkers and the latest neuropathological assessment criteria are both costly and time consuming. A large part of this cost is from brain imaging, which is also not widely available. Here, collaboration is key to maximizing cost effectiveness and sharing of cohort data for investigative consistency.

Overall, OCT, OCTA, SLO, and retinal fundus imaging remain the current most promising modalities for clinical diagnosis and large‐scale screening due to their higher number of consistent findings, widespread availability, cost effectiveness, and ease of implementation. Pupillary response testing also holds potential as a cost‐effective, portable screening tool if the research approaches could be standardized, with larger cohort studies and longitudinal testing. A detailed comparison of these methods is presented in Table S11 in supporting information.

Brain‐related ocular changes have the potential to serve as non‐invasive, cost‐effective, reliable, and widely accessible biomarkers for AD in clinical applications. However, future studies should concentrate on measures that mitigate potential barriers to enhance clinical translation. A proposed study strategy using ocular imaging technology as a source of biomarkers for AD is shown in Table 3.

**TABLE 3 alz70476-tbl-0003:** Proposed study strategy for minimizing challenges in ocular imaging for ad biomarkers.

Proposed strategy	Action plans
Standardize imaging protocols	Develop and implement standardized imaging protocols across research centers, including consistent image acquisition settings, processing techniques, image analysis software, AI‐based automated quantification methods, and quality control measures.
Harmonize imaging devices and address gaps in operator expertise	Encourage multi‐center studies using harmonized imaging systems or apply cross‐device calibration techniques to minimize inter‐device variability. Improve operator proficiency through joint training programs to ensure more consistent image acquisition across collaborative sites. Develop AI‐driven automated image acquisition and analysis tools to minimize reliance on operator expertise and provide more objectivity.
Increase study sample size on diverse cohorts	Conduct studies on large sample sizes, increase cohort sizes through multi‐center collaborations, population‐based studies, and longitudinal study designs to improve statistical power.
Conduct longitudinal studies	Implement longitudinal imaging studies with repeated measures to track disease progression and validate ocular biomarkers over time.
Investigate effects of co‐pathologies and confounding ocular conditions on AD‐related changes	Establish rigorous inclusion/exclusion criteria, consider sub‐group analysis (e.g., AD with and without glaucoma), and collect detailed ophthalmic history. Application of AD‐specific molecular contrast agents through intravenous injection or ophthalmic eye drops before in vivo imaging may enhance the detection of disease‐specific ocular changes associated with AD pathology.
Validate AD‐related ocular changes against gold‐standard AD biomarkers	Identify ocular imaging markers that correlate with established AD biomarkers (e.g., PET brain Aβ, CSF Aβ/tau) and cognitive assessments to validate clinical relevance.
Promote data sharing across multi research centers	Promote data sharing between research centers and implement quality controls, similar to other well‐established large‐scale studies, to enable consistent data comparability and replicability.
Minimize reliance on expensive imaging technology	Prioritise the development of cost‐effective imaging modalities like OCT and fundus scanning for early large‐scale screening. Emerging techniques, which tend to be inherently more costly and less available, should be reserved for validation studies until such time that their performance is proven. Collaborations should be initiated with studies that measure established AD biomarkers to recruit participants for ocular investigations.

Abbreviations: Aβ, amyloid beta; AD, Alzheimer's disease; AI, artificial intelligence; CSF, cerebrospinal fluid; OCT, optical coherence tomography; PET, positron emission tomography.

## 9 CONCLUSIONS

This review compiles recent studies examining ocular biomarkers for AD. The eye is the only body part in which blood vessels and neural tissue can be non‐invasively viewed or imaged with high resolution. Due to the intimate connection with the brain, examining the eye can reveal information about the brain. Therefore, the immediate need for inexpensive, accurate dementia diagnosis could potentially be met, even if partially, by ocular biomarkers. However, the contradictory results of some studies weaken the case for their effectiveness. In this review, we noted a diverse range of investigative approaches. However, consistency is needed in key areas of implementation to maximize the benefits of using this highly accessible part of the body to diagnose AD.

## CONFLICT OF INTEREST STATEMENT

The authors declare no conflicts of interest. Author disclosures are available in the supporting information.

## CONSENT STATEMENT

This article does not contain original or personally identifying data of human participants. Therefore, consent of study participants is not applicable for this review article.

## Supporting information



Supporting Information

Supporting Information
