# Supplementary material for: Ocular changes as potential biomarkers for early diagnosis of Alzheimer's disease
Source: Alzheimers Dement. 2025 Aug 24;21(8):e70476. doi: 10.1002/alz.70476 (PMC12375435; doi:10.1002/alz.70476)
Supplement: Supplementary file 2 — Supporting Information [file ALZ-21-e70476-s002.docx]

**Supplementary Materials**

**Ocular changes as potential biomarkers for early diagnosis of Alzheimer’s disease**

**Poudel et al. (2025)**

# Supplementary Section 1: Cornea

The cornea is the transparent exterior layer of the eye, which serves as the first refractive media and is also a protective layer for the sensitive anterior chamber. Whilst it is made up of multiple functional layers, neurologically, it also contains three nerve layers: sub-basal nerve plexus (between the basal epithelium and Bowman's layers), sub-epithelial nerve plexus (between the Bowman's layer and the anterior stroma) and stromal nerve plexus (within the stroma) (see Supplementary Figure 1).^1^ Corneal nerves originate from the ophthalmic branch of the trigeminal nerve and consist of both tiny C- and A-delta fibres.^2^ These sensory nerves are especially dense in the cornea and possess nociceptors, which are triggered by noxious mechanical, chemical, and thermal stimuli. Like other branches of the trigeminal nerve, they project into the trigeminal brainstem sensory complex. Thus, they form part of the peripheral nervous system (PNS), which is responsible for sensation in the face and eyes and its associated motor functions. Because of this extensive innervation, it was proposed that corneal structure and nerve density changes could be used for AD detection and monitoring.


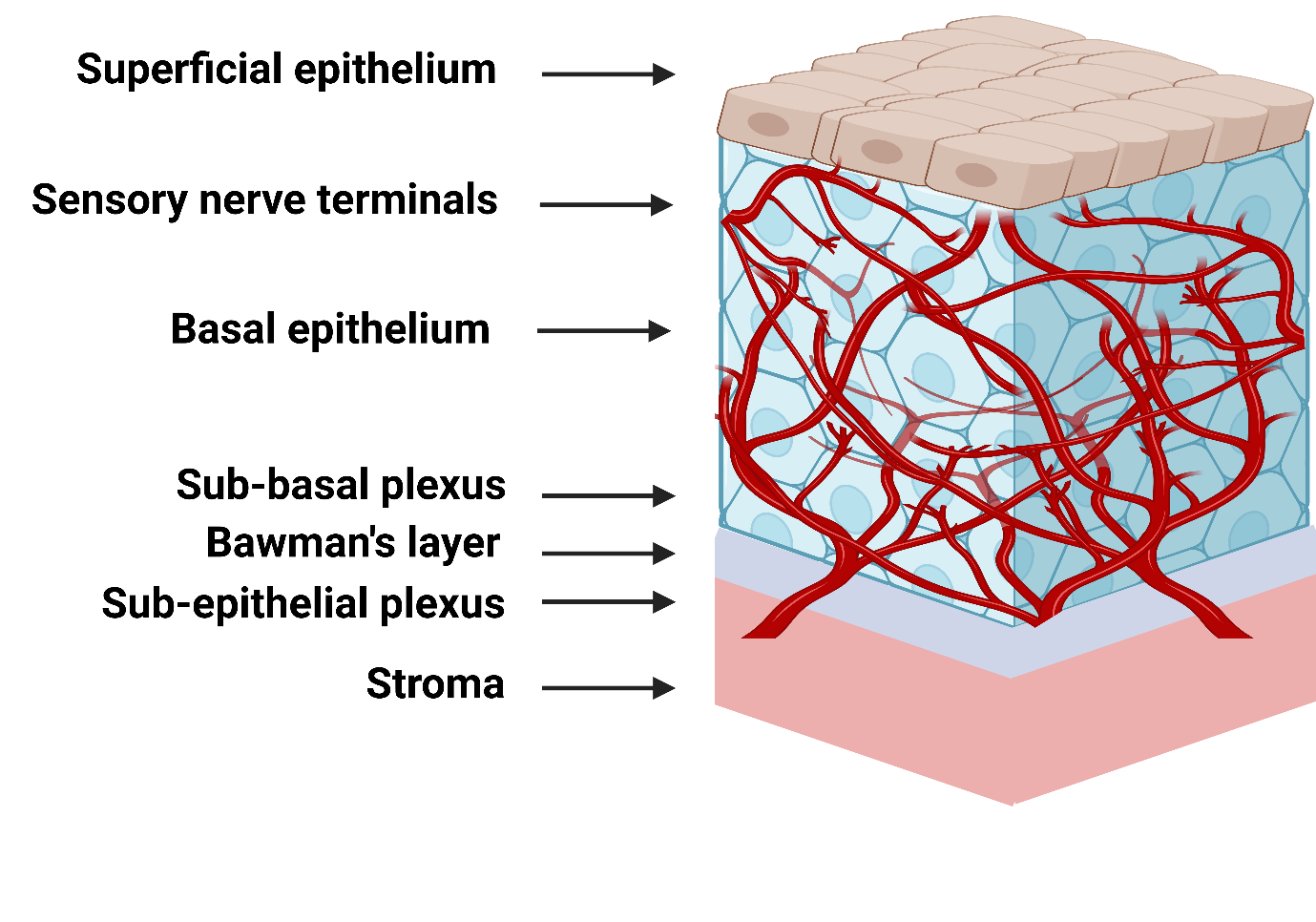


**Supplementary Figure S1: Corneal layers and corneal nerves distribution**: red lines depict the branching of the corneal nerves, which penetrate the different layers of the cornea with an abundance of corneal nerve fibers.

# Supplementary Section 2: Eye Movements in Alzheimer’s Disease Study


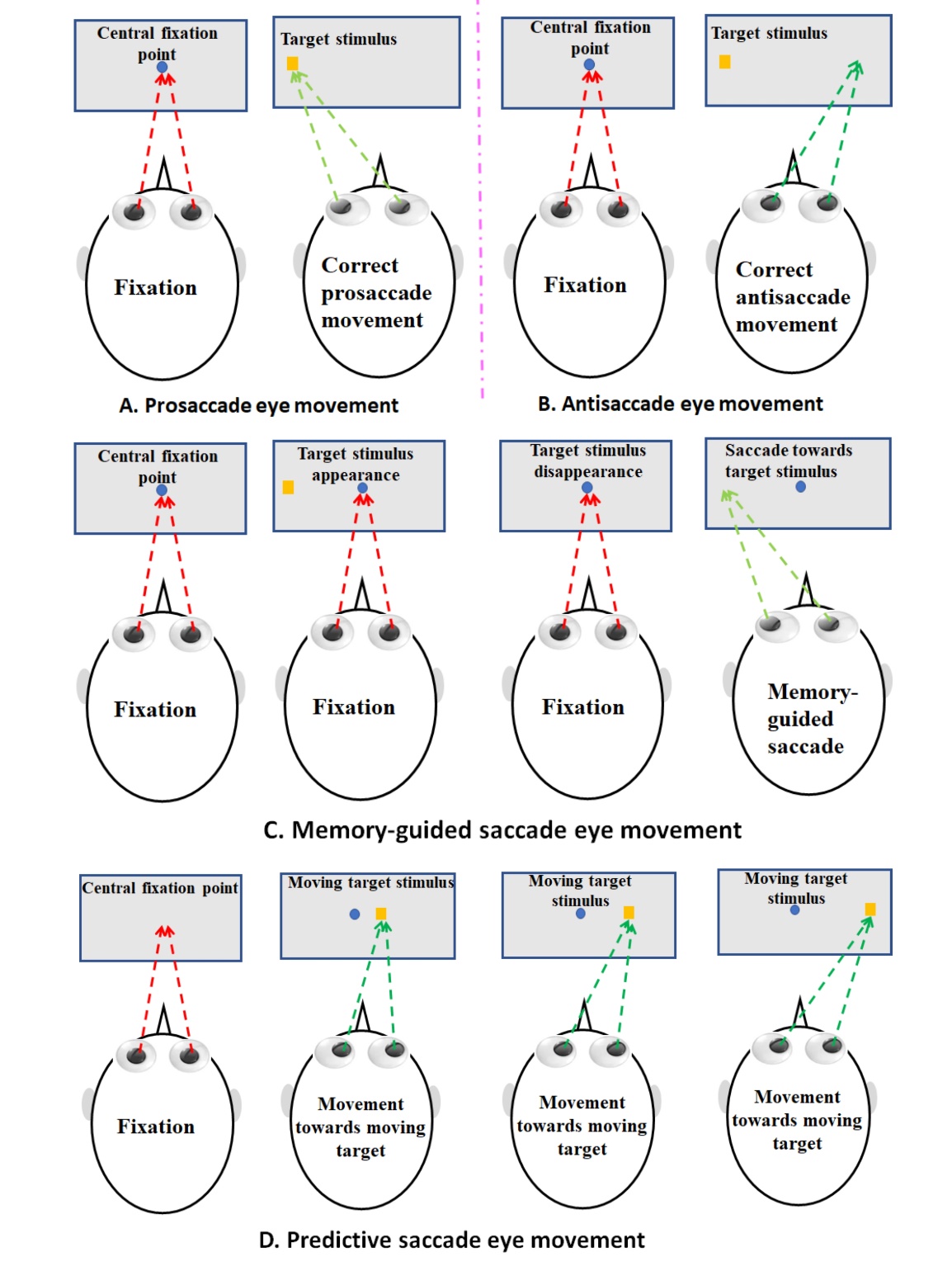


**Supplementary Figure S2: Saccadic paradigms:** (**A**) Visually guided saccade or prosaccade: a random visual stimulus is presented to one of the sides of the central fixation point, and participants are instructed to respond with rapid and precise eye movements. (**B**) Antisaccade: The eye movements are pointed in the direction of a location in the visual field that contrasts the stimuli. (**C**) Memory-guided saccade: participants are instructed to respond to a visual stimulus from central fixation based on their memory after the offset of the visual stimulus and central fixation. (**D**) Predictive saccade: a visible target moves through spatial variations in a specified time frame.^3^


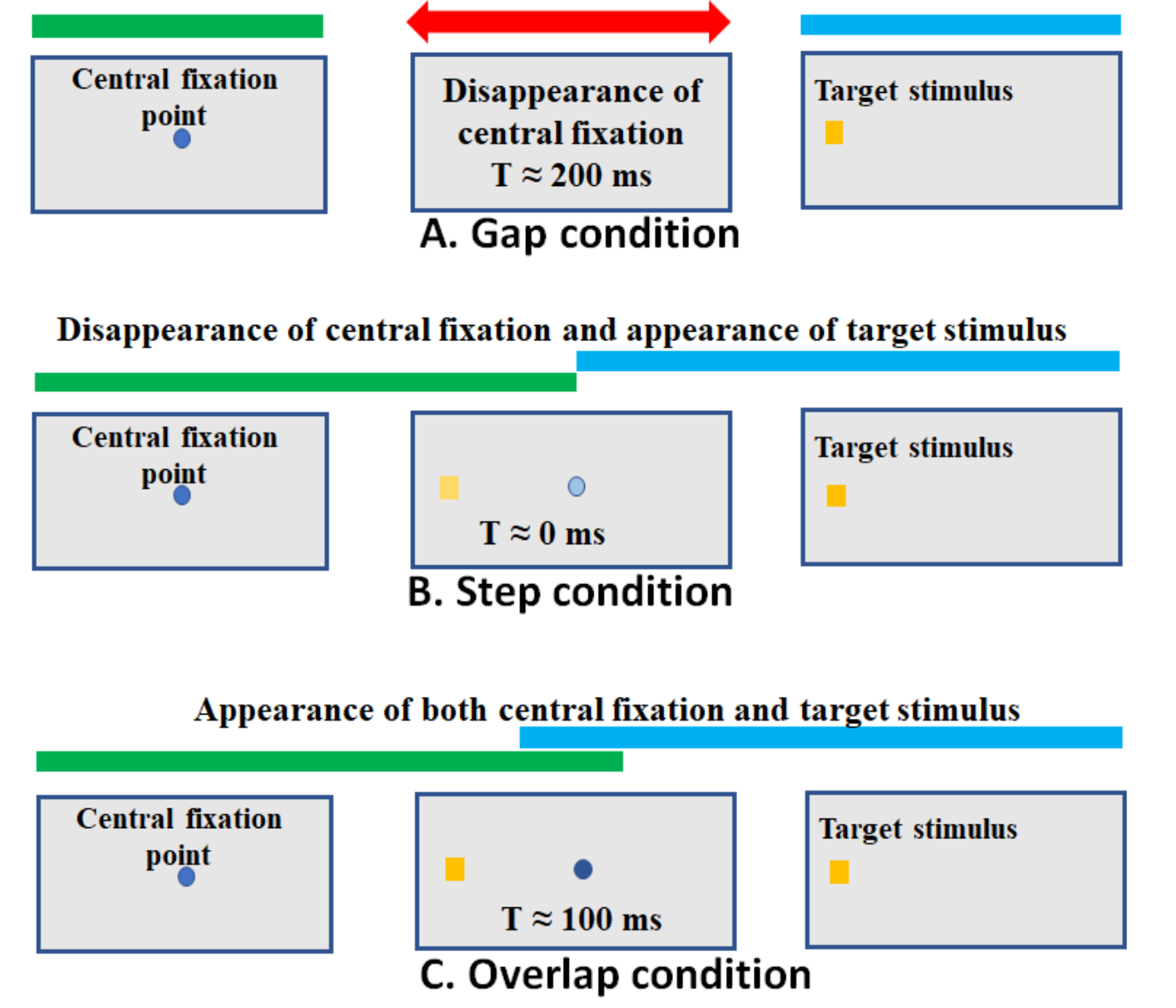


**Supplementary Figure S3:** **Conditions for different saccade paradigms**: Examples of a trial for saccade paradigms, illustrating (A) gap, (B) step and (C) overlap conditions.^3^

# Supplementary Section 3: Retina and Optic Disc

The retina is a multi-layered sensory structure found at the back of the eye. It is composed of ten different layers tasked with converting light into electrical impulses for transmission to the brain for further processing. Light focused on the retina passes through each of the following layers in succession: inner limiting membrane (ILM), retinal nerve fiber layer (RNFL), ganglion cell layer (GCL), inner plexiform layer (IPL), inner nuclear layer (INL), outer plexiform layer (OPL), outer nuclear layer (ONL), external limiting membrane (ELM), photoreceptive cell layers (PCL), and retinal pigment epithelium (RPE) (see Supplementary Figure 4).^4^ These tissue layers contain three different types of cells: photoreceptors, neurons, and glia.^4^

Ganglion cells are the neurons that project the converted information to the brain. Their non-myelinated axons form the RNFL and gather at the area forming the optic disc. These axons are myelinated by oligodendrocytes, rather than Schwann cells like peripheral nerves, upon exiting the ocular globe and forming the optic nerve.^5^ This continues as the optic tract and connects within the brain to the lateral geniculate nucleus, pretectal nuclei, and superior colliculus, which distribute the visual information to various brain regions for processing.^6^ Although the optic nerve is classified as the second of twelve pairs of cranial nerves, anatomically and developmentally, it is part of the central nervous system instead of the peripheral nervous system.^7^ As such, it is sheathed in all three meningeal layers (dura, arachnoid and pia mater), unlike peripheral nerves.^8^ Indeed, the retinal vasculature, the optic nerve, and the central nervous system share several similar characteristics.^7,9^ While the majority of AD pathology occurs in the brain, its disease markers have also been found in parts of the eye. These include the cornea, choroid, and retina, all of which are more accessible for imaging compared to the brain.^10^ Observation of changes in the retina and its biology might pave the way for the development of non-invasive, differential AD diagnostic methods. Figure 2 depicts AD-associated retinal changes observed through non-invasive imaging methods.


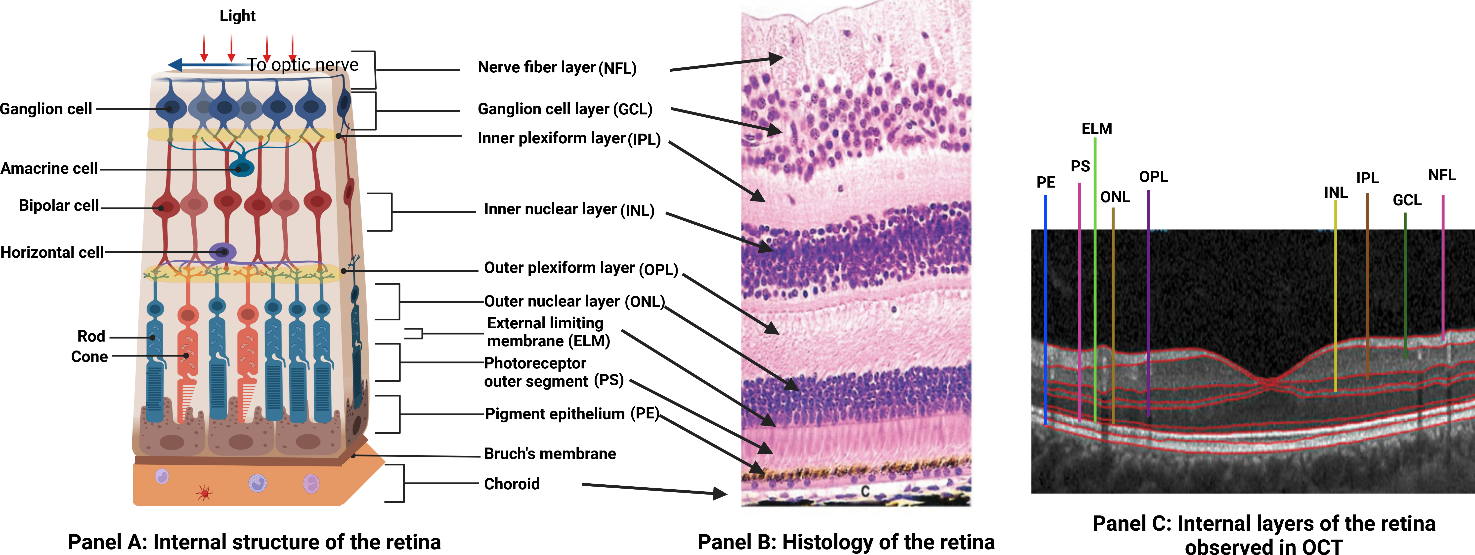


**Supplementary Figure S4: Internal structure of the retina showing different retinal layers**.

# Supplementary Section 4: AD Associated Retinal Changes Observed in Ex Vivo Studies

1. **Amyloidopathy through ex vivo methods**

Alzheimer’s disease is characterized by the formation of intercellular Aβ plaques and intracellular hyperphosphorylated tau neurofibrillary tangles in neurons.^11^ The Koronyo-Hamaoui *et al.* (2011)^12^ study was the first to identify Aβ plaques in the *post mortem* retina of definitive and probable or possible AD cases. In their subsequent immunohistochemical studies, they identified significantly more retinal Aβ42 deposition in AD and MCI patients than in controls. This was strongly associated with increased retinal macrogliosis, microgliosis, and tissue atrophy. These conditions were abundant in the superior and inferior temporal regions of the retina, with significant accumulations in the inner layers and peripheral subregions of MCI and AD compared to healthy retinas.^13,14^ Cognition was found to be associated with all retinal changes, where retinal Aβ42, far-peripheral AβOi, and microgliosis showed the highest correlations.^14^

Similar studies on retinal immunohistochemical analyses using Aβ-specific monoclonal antibodies and Aβ fluorescent binding compounds, such as curcumin, Congo-Red, Thioflavin-S, and Gallyas silver stain, revealed that all neuropathologically proven AD cases had retinal Aβ deposits in the form of oligomers or plaques.^12-20^ Some studies also detected retinal Aβ accumulations along blood vessels (perivascular), with some cases demonstrating significant deposits.^13,17^ Quantitative analysis showed a 2.74^16^ and 4.7-fold^13^ increase in Aβ42-containing plaque deposits in the retina of AD patients compared to age- and sex-matched controls. Furthermore, biochemical studies provided additional evidence by demonstrating increased amounts of Aβ in the retinas of AD patients.^14,17,21,22^ Retinal Aβ42 and Aβ40, as measured using ELISA, were significantly higher in AD patients compared to age- and sex-matched HCs.^14,17^ Additionally, high molecular weight species of Aβ42 and Aβ40 have also been found in the retina and associated hippocampal tissue of individuals carrying the APOE ɛ4 genotype,^22^ further demonstrating the close connection between eye and brain. However, a few studies did not exhibit discernible differences between control and AD-affected tissues for the retinal APP and Aβ pathology.^23-26^ This discrepancy may be due to differences in tissue processing technique and sensitivity,^23-26^ small study size,^23-25^ or the use of fewer tissues in the cross-sections than in whole-mount sample preparations^24^ and lower concentrations of Aβ-binding antibodies.^25^ These findings emphasize the importance of employing standardized experimental techniques to detect AD-related retinal Aβ pathology in the human retina. Overall, collaborative works that integrate diverse research methodologies are essential to minimizing methodological inconsistencies in studying retinal pathologies associated with AD.

In studies of AD participants and AD transgenic mouse models, both the blood-brain barrier^27^ and the blood-retinal barrier^17,28^ were found to be impaired. A considerable loss of pericyte cells in *post mortem* AD retinas was observed^17^, with significant Aβ deposition in pericytes and the microvasculature. Their study further revealed an early and gradual reduction in vascular platelet-derived growth factor receptor-β (PDGFR-β) in participants with MCI and AD, than in cognitively normal controls. A transgenic mouse study^28^ illustrated the probable processes of retinal vascular degeneration, and reported a significant degeneration in the retinal capillaries of AD transgenic mice compared with age- and sex-matched wild-type mice.

Similar to the human AD retina, multiple forms of Aβ have been identified in the retinas of AD mouse models, including APP_SWE_/PS1_∆E9_, Tg2576, PSAPP, 3xTg-AD, and 5xFAD.^12,19,21,29-32^ In a biochemical analysis study, Alexandrov *et al.* (2011)^21^ reported increased βAPP, Aβ40, and Aβ42 peptides and the innate immune and inflammatory response-regulator complement factor H (CFH) levels in the brain and retina of four different Tg-AD models: Tg2576, PSAPP, 3xTg-AD, and 5xFAD. These transgenic mouse models developed retinal Aβ deposits at a range of ages. The Tg2576 mice showed deposits at ten months whilst 5xFAD mice showed the earliest onset at two months. Although Tg2576 and 5xFAD mice had the highest retina-to-brain Aβ peptide ratios, the 5xFAD mice showed the highest concentration of Aβ peptides and the lowest amounts of CFH in both the brain and the retina. CFH was also found to be downregulated in human AD brains, suggesting an impaired ability to regulate the complement system and an elevated susceptibility to inflammation and neuronal damage.^33^ Overall, this suggests that 5xFAD is not only a useful model for studying the mechanisms of Aβ-mediated pathology in the brain but also for examining AD-related retinal changes.^21^ An immunohistochemical study using 5xFAD mice showed that soluble Aβ oligomers (Aβo), which are believed to be a primary cause of AD pathophysiology, are more prevalent in the nuclear retinal layers.^29^ This study also demonstrated that age-dependent Aβo accumulation in the retina is inversely proportional to the deposition of retinal Aβ plaques. Similarly, in another ex vivo study, Vandenabeele *et al.* (2021)^30^ demonstrated that soluble Aβ accumulates in the retina of *App^NL^*^-^*^G^*^-^*^F^* mice at an earlier age and progresses to the growth of Aβ plaques by mid-life. This increasing Aβ load corresponds with microglia reactivity, astrogliosis, and changes in retinal vein morphology. They also reported a distinct pre-plaque stage of AD during which only soluble forms of Aβ exist, followed by a stage where soluble, oligomeric types of Aβ are most likely in equilibrium with Aβ plaques. The changes in the brain and retina of mouse models are interesting and can reflect the human AD pathophysiology and help to gain insights into disease development and progression.

1. **Tauopathy through ex vivo methods**

In addition to retinal-Aβ-related pathologies, some studies have revealed different tau species including hyperphosphorylated (pTau) deposition in the retina of AD patients and the retina of mouse models.^16,23,25,31,34^ One study suggested that the development of retinal tau in transgenic mice preceded that of tau in the brain.^34^ Some studies reported the accumulation of pTau tangles in the GCL of pre-symptomatic triple transgenic mice, and this accumulation increased significantly as the disease progressed.^31,34^

Most studies on human *post mortem* retinas of AD individuals were able to identify diffuse pTau deposits but not fibrillar or other forms of tau.^16,23,25^ A study^23^ reported diffuse forms of pTau in IPL and OPL, which were more prominent in the superior regions than in the medial parts of the retina, with a positive gradient towards the periphery. Similar diffuse pTau staining was recorded in the GCL, IPL, INL and OPL of AD retinas following immunofluorescence staining.^25^ Most studies^35-38^ have identified various tau isoforms in the retina, including total tau, paired helical filaments (PHFs), pretangles, oligomeric tau, and phosphorylated tau (p-tau). However, only a limited number of investigations^16,36,37^ have reported the presence of neurofibrillary tangle-like structures across different retinal layers (Figure 3). One study found that individuals with AD, primary tauopathies, and tau pathology in the brain had higher amounts of tau phosphorylated at epitopes Ser202/Thr205 and Thr217 in the retina compared to both HCs and those with other neurodegenerative diseases.^38^ These epitopes showed a positive correlation with the hippocampal and cortical pTau (Ser202/Thr205) burden and Braak staging..^38^ Furthermore, Elevated levels (1.8–9.2-fold) of oligomeric and citrullinated tau isoforms, along with increased MC-1+ tau tangles, predominantly in the form of pretangles such as S202/T205, S214, S396, S404, and T231, were identified in the retinas of individuals with MCI and AD than in HC.^36^ Also, a recent *post mortem* study^35^ analyzing 18 retinas from AD, 143 from HC and 3 from individuals from other neurodegenerative conditions identified distinct stages of retinal tau pathology with a molecular composition distinct from species found in cerebral tauopathies. The severity of retinal pTau pathology was significantly associated with AD (odds ratio = 3.193 which means AD retinas are 3.193 times as likely to develop the tau pathology compared to non-AD, *P* = .001). These findings indicate that the accumulation of pathogenic tau species in the retina reflects the presence of tau pathology in the brain. In contrast, some previous investigations failed to show the existence of tau in the retina of individuals with AD,^24,26^ potentially due to variations in sample size, use of non-optimal techniques, analysis of limited anatomical regions, and different analytical tau isoforms. Developing a more advanced, non-invasive imaging method for finding specific retinal tauopathy could serve as an additional biomarker for tracking the progression of the disease. However, further research is necessary to gain a comprehensive understanding of tauopathy in human retinas, including its localization, progression (from immature to mature forms), propagation, and impact on retinal inflammation and degeneration.

# Supplementary Tables

## **Supplementary Table S1**: Studies on the changes in pupillary responses associated with AD.

| **Publication** | **Method** | **Sample Size** | **Significantly different parameters** | **Non-significant parameters** |
| --- | --- | --- | --- | --- |
| Prettymann *et al*. (1997)^39^ | Pupillary flash response | AD= 9, HC = 9 | ↓Resting pupil diameter, ↓amplitude and velocity in AD | Latency of the reflex response |
| Fotou *et al*. (2000)^40^ | Pupillary flash response | AD= 10 (5 under medication and 5 medications free), HC = 9 | ↓Latency and amplitude for maximum constriction in medication-free AD patients compared to HC | Latency and amplitude between AD patients under medication and HC |
| Granholm *et al*. (2003)^41^ | Pupillary flash response | AD = 15, HC = 15 | ↓Constriction amplitude | Resting pupil diameter and latency to minimum pupil size |
| Fotou *et al*. (2007)^42^ | Pupillary flash response | AD = 23, HC = 23 | ↑Constriction latency, ↑latency to maximum velocity, ↑latency to minimum pupil size, ↑constriction amplitude, ↓maximum constriction velocity and acceleration | Resting pupil diameter and minimum pupil size |
| Frost *et al*. (2013)^43^ | Pupillary flash response | AD = 19, HC = 17 | ↓Constriction velocity and acceleration, ↑latency to min pupil Size, ↓constriction amplitude, ↓ resting pupil size, ↓mean dilation velocity, ↓75% recovery time and ↑ percentage recovery 3.5 seconds after stimulus | Resting pupil diameter and minimum pupil size |
| Frost *et al*. (2013)^44^ | Pupillary flash response | APPGlu693Gln carriers = 6, APPGlu693Gln non-carriers = 6 | ↑75% recovery time and ↓ percentage recovery 3.5 seconds after stimulus | NA |
| Bittner *et al*. (2014)^45^ | Repetitive light stimulation | AD =66, MCI = 42, HC = 44 | NA | Pupil size, latency, and amplitude between the groups |
| Frost *et al*. (2017)^46^ | Pupillary flash response | For cross-sectional: AD = 14, cognitively healthy Aβ+ = 38, Aβ− = 77; For longitudinal: cognitively healthy Aβ+ = 11, Aβ− = 19 | *AD vs cognitively healthy (Aβ+ and Aβ−): ↓maximum acceleration, ↓maximum velocity, ↓average velocity, and ↓constriction amplitude; * cognitively healthy Aβ+ vs Aβ−: ↓maximum velocity; * Over 3 years: ↓maximum acceleration and ↓maximum velocity in cognitively healthy Aβ+ group | * cognitively healthy Aβ+ vs Aβ−: maximum acceleration, average velocity, and amplitude; *Longitudinal: average velocity and amplitude in cognitively healthy Aβ+ group and all pupillary parameters for Aβ− groups over 3 years |
| Kremen *et al*. (2019)^47^ | Task-evoked pupillary response | Cognitively normal people with high risk of AD based on AD-PRSs = 539 | Pupil dilation was significantly increased when cognitive demand was elevated in cognitively normal individuals who were at a higher genetic risk for AD, as indicated by the AD-PRS. | NA |
| Oh *et al*. (2019)^48^ | Chromatic pupillometry | Pre-AD (CSF Aβ_42_/Tau ratio biomarker positive) = 10, HC = 10 | PLR variability in the pre-AD group compared to control in blue light | Baseline and peak pupil size, sustained response, difference of the PLR between pre-AD compared to HC on chromatic pupillometry. |
| Kawasaki *et al*. (2020)^49^ | Pupillary flash reflex using portable chromatic pupillometer | AD = 16, HC = 16 | ↓Baseline pupil size | Pupillary contraction amplitude to all red and blue lights |
| Haj *et al*. (2022)^50^ | Cognitive task guided pupillary response | AD = 24, HC = 24 | ↓Variations in pupil size across the different cognitive tasks, such as forward span, backward span and counting of digits | NA |
| Gramkow et al. (2024)^51^ | Pupillary flash response | AD = 107, HC = 50 | ↓ baseline pupil diameter, ↓peak constriction pupil diameter, ↑latency, ↓relative pupil change, ↓constriction velocity, ↓maximum constriction velocity, ↓dilation velocity in AD than in HC | time till 75% of baseline value |
| Opwonya et al. (2024)^52^ | Task-evoked pupillary response | MCI = 213, HC = 514 | ↓pupil dilation in response to stimuli, ↓pupil dilation in antisaccade task (AS), ↑constriction magnitude in AS and ↓pupil size during AS in MCI than in HS | Baseline pupil size, pupil dilation difference between task conditions, |

Abbreviations: AD, Alzheimer’s disease; MCI, mild cognitive impairment; HC, healthy controls; Aβ+, brain amyloid beta positive; Aβ−, brain amyloid beta negative; CSF, cerebrospinal fluid; PLR, pupillary light response; AD-PRS, AD polygenic risk scores; APPGlu693Gln, Amyloid-Beta Precursor Protein genetic mutation; NA, not available.

## **Supplementary Table S2**: Studies on detecting AD-related changes in the lenses of human and animal models.

| **Publication** | **Method** | **Sample Size** | **Major findings** |
| --- | --- | --- | --- |
| Frederikse *et al*. (1996)^53^ | Immunohistochemistry, Immunoblotting | NA | βAPP and Aβ levels in mammalian lenses rise in response to H_2_O_2_ or UV radiation, indicating their potential involvement in the process by which oxidative damage causes lens opacification. |
| Goldstein *et al*. (2003)^54^ | Immunohistochemistry, slit lamp stereomicroscopy | AD = 9, HC = 8 | *↑Deposition of electron-dense Aβ-immunoreactive aggregates within lens fibre-cell cytoplasm in the supranuclear subregion of lenses of individuals with AD than in HCs; *AD-associated supranuclear cataract, concentrations of Aβ_1–42_ and Aβ_1–40_ in the human lens was equivalent to those in the aged human cerebral cortex; *Aβ amyloidopathy was not detected in non-AD lenses |
| Moncaster *et al*. (2010)^55^ | Immunohistochemistry, slit lamp stereomicroscopy | Down syndrome (DS) = 15, HC = 34 | * DS-associated supranuclear cataract (SNC); *↑Aβ peptides in DS (≈AD) vs HC lenses; *Aβ and amyloidopathy co-localize in SNC, DS lenses; *Cytosolic Aβ aggregates in DS lens fibers; *Aβ amyloidopathy not detected in non-AD, HC lenses |
| Kerbage *et al*. (2013)^56^ | Fluorescent ligand Eye laser scanning | AD =5, HC = 5 | * The fluorescent signature specifically associated with binding of Aβ in the supranuclear regions were twofold higher among AD participants as compared to HCs; * the deeper supranuclear region exhibited a stronger signal compared to other supranuclear regions in both groups. |
| Michael *et al*. (2013)^57^ | Immunohistochemistry, dark field stereomicroscope | AD = 21, HC = 15 | *No traces of beta-amyloid were detected in cataract lenses from donors with AD; * No traces of beta-amyloid were detected in cataract lenses from control donors. |
| Ho *et al*. (2014)^24^ | Immunohistochemistry | AD = 21, HC = 6 | *No amyloid deposits or abnormal tau accumulations were observed in the lens, retina, or other ocular structures of AD patients; * Findings indicated that β‐amyloid, phospho‐tau and α‐synuclein either do not deposit in the eye in a manner similar to that of the brain or are present at reduced levels or in altered forms. |
| Michael *et al*. (2014)^58^ | Immunohistochemistry, Raman Microscopy | AD = 7 (1 had pronounced bilateral cortical lens opacities, 1 moderate and 5 only minor or no opacities) | *Absence of amyloid-β in cataracts of AD patients; *The β-sheets that characterize Aβ are significantly less abundant in lens opacities compared to brain plaques; *Amyloid-β fibrils significantly alter the Raman profile of proteins and lipids; * Cortical lens opacities are not specific to persons with Alzheimer’s disease. |
| Kerbage *et al*. (2015)^59^ | Fluorescent ligand Eye laser scanning | AD = 20, HC = 20 | ↑Fluorescence signature in AD patient lenses compared to HC. |
| Bei *et al*. (2015)^60^ | Scheimpflug photography | Aβ+ = 15, Aβ−= 27 | Participants who were positive for AD biomarkers exhibited more advanced cataracts and increased cortical light scattering, however, there was no statistical significance after age adjustment. |
| Williams *et al*. (2017)^26^ | Immunohistochemistry | AD = 17, HC = 2 | No indication of inclusions, deposits, or other accumulation of proteins in any case, in any part of the globe. |
| Moncaster *et al*. (2022)^61^ | Immunohistopathology, amyloid histochemistry, immunoblot analysis, epitope mapping, immunogold electron microscopy, quantitative immunoassays, and tryptic digest mass spectrometry peptide sequencing | Down syndrome participants = 21; participants for quantitative traits in lens and brain analysis = 1249; Tg2576 transgenic mice = 4 and littermate control mice =4 for lens explant culture; mice for light scattering spectroscopy = 20 (5 groups) | * Expression of human APP, accumulation of hAβ peptides, age- dependent Aβ supranuclear lens opacification and development of hAβ molecular and ultrastructural pathologies in the transgenic mice; *detected hAβ in conditioned medium obtained from lens explant cultures prepared from Tg+ mice, but not Tg− control mice. This result is consistent with constitutive hAβ generation in the lens; **In-vitro* studies demonstrated that hAβ stimulated mouse lens protein aggregation detected by quasi-elastic light scattering (QLS) spectroscopy. |

Abbreviations: AD, Alzheimer’s disease; MCI, mild cognitive impairment; HC, healthy controls; Aβ+, brain amyloid beta positive; Aβ−, brain amyloid beta negative; βAPP, beta-amyloid precursor protein; NA, not available.

## **Supplementary Table S3**: AD-related corneal changes in animal and human models at different studies.

| **Publication** | **Method** | **Sample Size** | **Significantly different parameters/Major findings** | **Non-significantly different parameters** |
| --- | --- | --- | --- | --- |
| Frederikse and Zigler (1998)^62^ | Immunohistochemistry, western blotting | NA | Presenilin mRNA in the mouse and monkey cornea | NA |
| Dutescu *et al*. (2009)^63^ | Immunocytochemistry | NA | Cytoplasmic expression of APP and probably Aβ in corneal epithelia for transgenic mice. | NA |
| Ornek *et al*. (2015)^64^ | Corneal esthesiometer | AD = 20, HC = 32 | ↓ mean corneal sensitivity in AD than in HC | NA |
| Cesareo *et al*. (2015)^65^ | SLO (Heidelberg Retinal Tomograph III) | AD = 51, HC = 67 | NA | Central corneal thickness |
| Choi *et al*. (2019)^66^ | Cell culture and biochemistry | NA | Both corneal fibroblasts and the corneal epithelium showed the expression of APP, along with proteins involved in APP processing and degradation of Aβ. | NA |
| Ponirakis *et al*. (2019)^67^ | Corneal confocal microscopy | Dementia = 26, MCI = 30, and HC = 20 | ↓Corneal nerve fiber length among AD, MCI, and HC groups; ↓fiber density between dementia vs HC and dementia vs MCI; ↓branch density between MCI vs HC. | Corneal nerve branch density between MCI vs HC; Corneal branch density between dementia vs MCI. |
| Dehghani *et al*. (2020)^68^ | Corneal confocal microscopy | AD = 5, MCI = 5 and HC = 5 | ↑Corneal DC field area and perimeter in people with MCI, compared to HC, ↓corneal DCs in the whorl region of MCI eyes exhibited reduced circularity and roundness indices and ↑aspect ratio compared to HC. | ↓Quantitative parameters for corneal nerve architecture in the AD and MCI groups than in HC participants. |
| Al-Janahi *et al*. (2020)^69^ | Corneal confocal microscopy | Dementia = 66 (AD = 19), MCI = 80, and HC = 36 | ↓corneal nerve fiber density, branch density, and fiber length in patients with MCI and dementia than in HC. | NA |
| Ponirakis *et al*. (2022)^70^ | Corneal confocal microscopy | Dementia = 66 (AD = 19), MCI = 80, and HC = 36 | ↓Corneal nerve fiber density, branch density, and fiber length in MCI vs HC and dementia vs HC; ↓corneal nerve fiber density in dementia vs HC | ↓corneal nerve branch density and fiber length in dementia vs HC |
| Ponirakis *et al*. (2023)^71^ | Corneal confocal microscopy | MCI progressed to dementia (MPD) = 107, MCI not progressed to dementia (NMD) = 74, and HC =12 | ↓Corneal nerve fiber density (CNFD), ↓corneal nerve branch density (CNBD), ↓corneal nerve fiber length (CNFL), ↓CNBD/CNFD in MPD compared to MND at baseline;↓CNFD, ↓CNBD, and ↓CNFL between MPD vs HC and MND vs HC at baseline;↓CNBD/CNFD between MPD vs HC at baseline;↓CNFD, ↓CNBD and ↓CNFL in participants with MCI who reverted to HC over 2.6 years compared to HC. | CNBD/CNFD in MND compared to HC at baseline; CNBD/CNFD in participants with MCI who reverted to HC over 2.6 years compared to HC; ↓CNFD, ↓CNBD, ↓CNFL and CNBD/CNFD between MCI who reverted to HC over 2.6 years compared to who remained in MCI |
| Gundogan *et al.* (2024)^72^ | Corneal confocal microscopy, Cochet-Bonnet esthesiometer for sensitivity measurement | AD = 22; HC = 18 | ↓nerve fiber length (NFL), ↓ nerve fiber density (NFD), and ↓nerve branch density (NBD); ↓corneal sensitivity | NA |

Abbreviations: AD, Alzheimer’s disease; MCI, mild cognitive impairment; HC, healthy controls; APP, amyloid precursor protein; DC, dendritic cells; mRNA, messenger RNA; NA, not available; SLO, scanning laser ophthalmoscope.

## **Supplementary Table S4**: Summary of studies on saccadic eye movement in participants with AD and MCI.

| **Study** | **Cohort size** | **Paradigm** | **Condition** | **Significant Variables** | **Non-Significant variables** |
| --- | --- | --- | --- | --- | --- |
| Alichniewicz et. al. (2013)^73^ | HC = 19,  aMCI = 23 | Prosaccade | Horizontal S | NA | Proportion of correct saccade, omission, and reaction time |
|  |  | Antisaccade | Horizontal S | ↓Proportion of correct saccade | Proportion of omission and reaction time |
| Baucart *et al*. (2014)^74^ | HC = 15, AD = 14 | Prosaccade | Horizontal | ↑Latency Gap | NA |
| Boxer *et al*. (2006)^75^ | HC = 20, AD = 18 | Smooth pursuit | Horizontal | ↓Gain, ↓acceleration | ↑Latency |
|  |  |  | Vertical | ↓Gain, ↓acceleration | ↑Latency |
|  |  | Prosaccades | Horizontal | ↑Latency O, | ↑Latency G, ↓gain, ↓velocity |
|  |  |  | Vertical | ↑Latency O, | ↓gain, ↑velocity |
|  |  | Antisaccade | Horizontal/G | ↓Correct response, ↓Errors corrected | Correct latency |
| Boxer *et al*. (2012)^76^ | HC = 27, AD = 10 | Prosaccade | Horizontal | ↑Latency O or G, | Velocity O or G, gain |
|  |  |  | Vertical | ↑Latency O or G, | Velocity O or G, gain |
|  |  | Antisaccade | Horizontal/G | ↓Correct response | NA |
| Chehrehnegar, et. al. (2019)^77^ | AD = 21, HC = 59, aMCI = 40 | Prosaccade | Horizontal | ↓Gain and between aMCI and control on G and O, ↑velocity between AD and HC groups on G. | Gain between aMCI and AD on G and O, velocity between HC vs aMCI and aMCI vs AD on G. |
|  |  | Antisaccade | Horizontal | ↓Gain between aMCI and HC on G and O, ↓velocity between AD and HC groups on G, ↑latency between AD vs HC and aMCI vs AD on G, latency between AD vs aMCI on O. | Gain between aMCI vs AD on G and O. |
| Crawford *et al*. (2013)^78^ | HC (elderly) = 18; HC (young) = 17; AD = 18 | Prosaccade | Horizontal G and O | NA | Latency, amplitude, omissions, and anticipations |
|  |  | Antisaccade | Horizontal G | Inhibition errors, uncorrected errors, and reaction time | Latency and omissions |
|  |  |  | Horizontal O | Uncorrected errors | Latency, omissions, inhibition error and reaction time |
| Crawford *et al*. (2015)^79^ | HC = 25, AD = 11 | Prosaccade | Horizontal G and O (longitudinal study) | ↑Reaction time in G condition, slower reaction time of AD and HC groups in O condition compared to G condition. | Gap effect (difference between overlap and gap latency) |
| Crawford *et al*. (2019)^80^ | HC = 95, AD = 42, MCI = 65 | Antisaccade | Horizontal G | ↑Errors followed by a correction, ↑errors with no correction in AD and MCI compared to the HC group, | Relative latency difference between correct and incorrect antisacades |
| Garbutt *et al*. (2008)^81^ | HC = 27, AD = 28 | Smooth pursuit | Horizontal | ↓Acceleration, ↓Gain | ↑Latency |
|  |  |  | Vertical | ↑Latency, ↓acceleration, ↓Gain | NA |
|  |  | Prosaccade | Horizontal | ↑Latency O and G, | Velocity O and G, gain O and G |
|  |  |  | Vertical | ↑Latency O | Velocity O, gain O |
|  |  | Antisaccade | Horizontal/G | ↓Correct response | Antisaccade latencies |
| Holden et. al. (2018)^82^ | HC = 27, AD = 23, MCI = 29 | Prosaccade | Horizontal | ↓Gain G and ↓correct saccade G between MCI and HC | Latency and velocity G among groups, correct saccade between MCI and AD, |
|  |  |  | Vertical | ↑Correct saccade S between MCI and AD | Correct saccade S between MCI and HC as well as MCI and AD, Latency S among groups |
|  |  | Antisaccade | Horizontal G | ↑Error and between MCI and HC, ↑self-correction rate of AD with MCI and groups, ↑latency of correct saccade between MCI and AD group. | Error between MCI and AD, latency of correct saccade between MCI and control groups. |
| Kaufman et. al. (2012)^83^ | AD = 30, HC = 31 | Prosaccade | Horizontal S | ↑Task errors | NA |
|  |  | Antisaccade | Horizontal S | ↑Task errors, uncorrected, fixation and omission errors | NA |
| Lage *et al*. (2020) ^84^ | AD = 18,  HC = 29 | Prosaccade | Horizontal or vertical G | ↑Prosaccade error, ↑Prosaccade latency | NA |
|  |  | Antisaccade | Horizontal or vertical G | ↑Corrected antisaccade latency, ↑antisaccade error, ↓percentage corrected antisaccades, ↓percentage successful antisaccades | NA |
|  |  | Smooth pursuit | Sinusoidal pursuit | ↑Horizontal pursuit error, ↑vertical pursuit error | Horizontal and vertical pursuit gain |
| Laurens et. al. (2019)^85^ | AD = 23, aMCI = 25, HC = 26 | Prosaccade | Horizontal O | ↑Error rates between AD vs HC and AD vs aMCI, ↑anti-saccade errors committed by aMCI and AD during prosaccade tasks | Shorter response latency in AD than in HCs and aMCI patients, anticipation rate or the percentage of express saccades |
| Lenoble et. al. (2015)^86^ | HC = 27 (elder), 26 (young); AD = 24 | Prosaccade with natural or urban scenes and indoor or outdoor scenes | Horizontal G | NA | Latencies among the three groups |
| Lenoble et. al. (2018)^87^ | Old Control = 12(elder), 12 (young), AD = 12 | Prosaccade towards the congruent or incongruent scene | Horizontal G | ↑Latency in AD group compared to others, saccade accuracy towards congruent objects among three groups | Saccade accuracy between AD and old control in implicit task-eye response. |
| Noiret et. al. (2018)^88^ | AD = 20, HC = 20 | Prosaccade | Horizontal or vertical S | ↑Latency, ↑Latency variability | NA |
|  |  | Antisaccade | Horizontal or vertical S | ↑Correct latency, ↑correct latency variability, ↓proportion of correct saccade, ↓proportion of corrected saccade, ↑time-to-correct saccade | Incorrect AS latency, Incorrect AS latency variability |
|  |  | Predictive saccades | Horizontal or vertical S | ↑Latency, ↑anticipated saccade gain | Non-anticipated saccade gain |
| Pavisic et. al. (2017)^89^ | Young ons*et* Alzheimer’s disease = 36 HC = 21 | Fixation | Central target | ↑Intrusive saccades, ↓fixation duration on target | Number of square wave jerks |
|  |  | Prosaccade | Horizontal and vertical | ↓Accuracy, ↑latency, ↑number of saccades to fixate the target | NA |
|  |  | Smooth Pursuit | Horizontal | ↓Spent time on pursing the target | NA |
|  |  |  | Vertical | ↓Spent time on pursing the target | NA |
| Polden and Crawford, (2023)^90^ | AD = 65, aMCI = 42, naMCI = 46, HC = 98 | Prosaccade | Horizontal G and O | NA | Prosaccade mean latencies and CV (coefficient of variation or the ratio of the standard deviation in relation to the mean) scores in gap and overlap condition among four groups. |
|  |  | Antisaccade | Horizontal G | ↑Antisaccade mean latencies and percentage error between AD vs. HC, AD vs. naMCI, aMCI VS naMCI and aMCI vs. Control groups. | Mean latencies and CV scores between AD vs. aMCI and HC vs naMCI, percentage error between AD vs. aMCI and HC vs naMCI. |
| Polden et. al. (2020)^91^ | AD = 32, MCI = 45, OEP= 96 | Prosaccade | Horizontal | ↑Reaction time G: AD vs. OEP, MCI vs. OEP | Reaction time O: AD vs. OEP, MCI vs. OEP; Gap effect (O latency – G latency): AD vs. OEP, MCI vs. OEP |
| Riek et. al. (2023)^92^ | AD = 24, MCI = 68, HC = 149 | Prosaccade | Horizontal G | ↑Disengagements (shifting gaze away from the fixation point displayed onscreen and failing to return to it) between MCI vs HC | Amplitude between AD, MCI, and HC groups, prosaccade task disengagements between AD vs HC |
|  |  | Antisaccade | Horizontal G | ↑Regular errors between MCI vs HC and AD vs HC, ↑voluntary override time (time after stimulus appearance) between MCI vs HC | Disengagements, expressed latency (guessing/anticipating saccade), express errors between three groups, voluntary override time (time after stimulus appearance) between AD vs, MCI and AD vs Control groups. |
| Shakespear et. al. (2015)^93^ | AD = 32, HC = 22 | Fixation | Central target | ↑Square wave jerk frequency, ↓Fixation period | Large intrusive saccade frequency |
|  |  | Prosaccade | Horizontal and vertical | ↑Peak velocity G or O | time to fixate on target, latency, amplitude error and number of saccades made on G or O condition |
|  |  | Smooth pursuit | Sinusoidal pursuit | ↓Pursuit gain and ↑number of saccades made | NA |
| Wilcockson et.al. (2019)^94^ | AD = 68, aMCI = 42, naMCI = 47, HC = 92 | Antisaccade | Horizontal G | ↑Latency between AD vs naMCI, aMCI vs naMCI and aMCI vs HC groups; ↑uncorrected error between AD vs HC and aMCI vs HC groups. | Latency between the aMCI vs AD and naMCI vs HC groups, uncorrected error between aMCI vs AD and naMCI vs HC groups, higher proportion of aMCI errors compared to the naMCI group errors. |
| Tao et al. (2024)^95^ | AD = 23,  MCI = 23,  HC = 20 | Prosaccade | Horizontal or vertical | AD vs HC: ↓Accuracy O, ↑latency O or G and ↑completion time O or G.  AD vs MCI: ↑latency O or G, ↑completion time O, | MCI vs HC: Accuracy, latency, completion time and saccade velocity in O or G; AD vs HC: saccade velocity O or G and Accuracy G; AD vs MCI: Accuracy O or G, saccade velocity O or G and completion time G, |
|  |  | Antisaccade | Horizontal or vertical | MCI vs HC: ↓Error correction; AD vs HC: ↓Error correction, ↓accuracy and ↓saccade velocity; AD vs MCI: ↓Error correction, ↑completion time and ↓accuracy. | MCI vs HC: Accuracy, latency, completion time and saccade velocity; AD vs HC: Saccade latency and completion time; AD vs MCI: latency and saccade velocity |
|  |  | Smooth Pursuit | Horizontal | NA | All the measured saccadic parameters |

Abbreviations: AD, Alzheimer’s disease; MCI, mild cognitive impairment; HC, healthy controls; aMCI, amnestic MCI; naMCI, non-amnestic MCI; OEP, older European participants; G, gap condition; O, overlap condition; S, step condition; NA, not available.

## **Supplementary Table S5**: Studies on detecting AD-associated choroidal changes in different subjects.

| **Publication** | **Method** | **Sample Size** | **Significantly different parameters** | **Non-significant** |
| --- | --- | --- | --- | --- |
| Bayhan *et al*. (2014)^96^ | OCT | AD = 31, HC = 30 | ↓Choroidal thickness | Choroidal thickness at 3.0 mm temporal to the fovea |
| Gharbia *et al*. (2014)^97^ | OCT | AD = 21, HC = 21 | ↓Choroidal thickness | NA |
| Bulut *et al*. (2016)^98^ | OCT | AD = 41, MCI = 38, and HC = 44 | ↓Choroidal thickness in both patients with AD and MCI than in HC. | Choroidal thickness between AD and MCI |
| Trebbastoni *et al*. (2017)^99^ | OCT | AD = 39, HC = 39 (longitudinal) | ↓Choroidal thickness (adjusted for baseline CT, age, sex, axial length, and smoking). | NA |
| Cunha *et al*. (2017)^100^ | OCT | AD = 50, HC1 = 152 (Mean Age =71.03 years), HC2 = 50 (Mean Age = 82.1) | ↓Choroidal thickness in AD patients compared to HC1 group. | Choroidal thickness in AD patients compared to HC2group. |
| Bulut *et al*. (2018)^101^ | OCT/OCTA | AD = 26, HC = 26 | ↓Choroidal thickness | Choroidal flow rate |
| Querques *et al*. (2019)^102^ | OCTA | AD = 12, MCI = 12, HC = 32 | NA | Choriocapillaris (CC) perfusion density and choroidal perfusion density among AD, MCI, and HC groups. |
| Haan *et al*. (2019)^103^ | OCT | Aβ+ = 48, Aβ− = 38 | NA | Choroidal thickness |
| López-De-Eguileta *et al*. (2020)^104^ | OCT | Amyloid positive = 63 (MCI = 51 and dementia = 12), and HC = 63 | ↓Choroidal thickness at five selected locations out of fourteen in amyloid positive than in HC. | Choroidal thickness at nine selected locations out of fourteen between amyloid positive and HC groups; Choroidal thickness at all choroidal locations among people with dementia, MCI, and HC groups. |
| Salobrar-Garcia *et al*. (2020)^105^ | OCT | AD = 17, HC =15 | ↓choroidal thickness at nasal, superior and inferior regions around macula of AD than in control group. | Choroidal thickness in temporal region of macula between AD and HC. |
| Robbins *et al*. (2021)^106^ | OCTA | AD = 67 (112 eyes), MCI = 74 (143 eyes), and HC = 137 (248 eyes) | After adjusting the covariates: ↑Total choroidal area and ↑luminal area in AD and MCI patients compared with HC; ↓ choroidal vascularity index in MCI compared to HC. | After adjusting the covariates: Sub foveal choroidal thickness among patients with AD and MCI and HC groups. |
| Zhang *et al*. (2021)^107^ | OCTA | AD = 25, HC = 20 | ↓Choriocapillaris flow density | NA |
| Li *et al*. (2021)^108^ | OCT | AD = 37, HC = 34 | ↓Choroidal thickness | NA |
| Ma *et al*. (2022)^109^ | OCT/OCTA | At baseline: APOE ε4 carriers = 98, APOE ε4 non-carriers = 120; After 2 years: APOE ε4 carriers = 71, APOE ε4 non-carriers = 78 | NA | Total choroidal area, luminal area, and choroidal vascularity index at baseline and after two years |
| Corradetti *et al*. (2024) ^110^ | OCTA | pathological Aβ_42_/tau individuals = 13; normal Aβ_42_/tau individuals = 10 | ↓choriocapillaris flow deficits in cases than in HC. | NA |

Abbreviations: OCT, optical coherence tomography; SD-OCT, spectral domain OCT; OCTA, optical coherence tomography angiography; AD, Alzheimer’s disease; MCI, mild cognitive impairment; HC, healthy controls; Aβ+, brain amyloid positive; Aβ−, brain amyloid negative; APOE, apolipoprotein E; SLO, scanning laser ophthalmoscope; NA, not available.

## **Supplementary Table S6**: Summary of studies evaluating the retinal structural changes in different subjects obtained through OCT, OCTA and SLO

| **Alterations** | **Imaging method** | **Significance difference between controls and cases** | **Subject** | **Study** |
| --- | --- | --- | --- | --- |
| pRNFL | OCT | Yes | AD | Bambo *et al*.^111^; Berisha *et al*.^112^; Chi *et al*.^113^; Cipollini *et al*.^114^; Cunha *et al*.^115^; Cunha *et al*.^116^; Eraslan *et al*.^117^; Garcia-Martin *et al*.^118^; Güneş *et al*.^119^; Iseri *et al*.^120^; Jindahra *et al*.^121^; Kirbas *et al*.^122^; Kromer *et al*.^123^; La Morgia *et al*.^15^; Larrosa *et al*.^124^; Lu *et al*.^125^; Marziani *et al*.^126^; Moschos *et al*.^127^; Mutlu *et al*.^128^; Parisi *et al*.^129^; Polo *et al*.^130^; Salobrar-Garcia *et al*.^131^; Shi *et al*.^132^; Trebbastoni *et al*.^133^; Yan *et al*.^134^; Zabel *et al*.^135^ |
|  |  |  | MCI | Criscuolo *et al*.^136^ |
|  |  |  | AD and MCI | Ascaso *et al*.^137^, Cheung *et al*.^138^; Choi *et al*.^139^; Ferrari *et al*.^140^; Gao *et al*.^141^; Kesler *et al*.^142^; Liu *et al*.^143^; Oktem *et al*.^144^;Paquet *et al*.^145^ |
|  |  |  | CSF biomarker + ve | Asanad *et al*.^146^ |
|  |  |  | Brain Aβ+ | Byun *et al*.^147^ |
|  |  | No | AD | Gharbia *et al*.^97^; Salobrar-Garcia *et al*.^148^; Golzan *et al*.^149^ |
|  |  |  | AD and MCI | Feke *et al*.^150^; Pillai *et al*.^151^; Yoon *et al*.^152^; Zhang et al.^153^ |
|  |  |  | Brain Aβ+ | Golzan *et al*.^149^; Snyder *et al*.^154^; Van de Kreeke *et al*.^155^; Van de Kreeke *et al*.^156^; Santos *et al*.^157^ (both baseline and longitudinal) |
|  |  |  | APOE ε4 carriers | López-Cuenca *et al*.^158^; Ma *et al*.^109^ |
|  | SLO | Yes | AD | Cesareo *et al*.^65^; Danesh-Meyer et al.^159^ |
|  |  | No | AD | Kurna et al.^160^ |
| mRNFL | OCT | Yes | AD | Cunha *et al*.^116^; Garcia-Martin *et al*.^118^; Salobrar-Garcia *et al*.^131^; Salobrar-Garcia *et al*.^148^; Shao *et al*.^161^; Sharma et al.^162^ |
|  |  |  | Brain Aβ+ | Santos *et al*.^157^ (longitudinal) |
|  |  |  | APOE ε4 carriers | López-Cuenca *et al*.^158^ |
|  |  | No | MCI | Shin *et al*.^163^ |
|  |  |  | AD and MCI | Querques *et al*.^102^ |
|  |  |  | APOE ε4 carriers | Shin *et al*.^163^ |
|  |  |  | Brain Aβ+ | Snyder *et al*.^154^; Van de Kreeke *et al*.^155^; Van de Kreeke *et al*.^156^; Santos *et al*.^157^ (baseline) |
| GCL | OCT | Yes | AD | Garcia-Martin *et al*.^118^; Lian *et al*.^164^; Salobrar-Garcia *et al*.^131^; Golzan *et al*.^149^; Sharma et al.^162^ |
|  |  |  | AD and MCI | Querques *et al*.^102^ |
|  |  |  | Brain Aβ+ |  |
|  |  | No | APOE ε4 carriers | López-Cuenca *et al*.^158^; Shin *et al*.^163^ |
|  |  |  | Brain Aβ+ | Snyder *et al*.^154^; Van de Kreeke *et al*.^155^; Van de Kreeke *et al*.^156^; Golzan *et al*.^149^; Santos *et al*.^157^ (both baseline and longitudinal) |
|  |  |  | MCI | Shin *et al*.^163^ |
| GCL-IPL | OCT | Yes | AD | Cunha *et al*.^115^; Jindahra *et al*.^121^; Mutlu *et al*.^128^; Shao *et al*.^161^ |
|  |  |  | AD and MCI | Cheung *et al*.^138^; Choi *et al*.^139^; Ferrari *et al*.^140^;Yoon *et al*.^152^ |
|  | OCT | No | CSF biomarker + ve | Asanad *et al*.^146^ |
|  |  |  | Brain Aβ+ | Byun *et al*.^147^ |
|  |  |  | AD and MCI | Jiang *et al*.^165^; Pillai *et al*.^151^ Querques *et al*.^102^ |
|  |  |  | APOE ε4 carriers | Ma *et al*.^109^ |
| RNFL-GCL-IPL | OCT | Yes | AD | Cipollini *et al*.^114^; Cunha *et al*.^115^; Eraslan *et al*.^117^; Bayhan *et al*.^96^; Farzinvash *et al*.^166^ |
|  |  |  | AD and MCI | Querques *et al*.^102^ |
|  |  |  | MCI | Criscuolo *et al*.^136^; |
|  |  |  | Cognitively impaired | Yang *et al*.^167^ |
| IPL | OCT | Yes | AD | Garcia-Martin *et al*.^118^; Sharma et al.^162^ |
|  |  |  | AD and MCI | Querques *et al*.^102^ |
|  |  |  | APOE ε4 carriers | López-Cuenca *et al*.^158^ |
|  |  |  | Brain Aβ+ | Santos *et al*.^157^ (longitudinal); ↑Snyder *et al*.^154^ |
|  |  | No | AD | Salobrar-Garcia *et al*.^131^ |
|  |  |  | Brain Aβ+ | Van de Kreeke *et al*.^155^; Van de Kreeke *et al*.^156^; Santos *et al*.^157^ (baseline) |
|  |  |  | APOE ε4 carriers | Shin *et al*.^163^ |
|  |  |  | MCI | Shin *et al*.^163^ |
| INL | OCT | Yes | APOE ε4 carriers | López-Cuenca *et al*.^158^ |
|  |  | No | AD | Garcia-Martin *et al*.^118^; Sharma et al.^162^ |
|  |  |  | MCI | Shin *et al*.^163^ |
|  |  |  | APOE ε4 carriers | Shin *et al*.^163^ |
|  |  |  | Brain Aβ+ | Snyder et al.^154^; Santos *et al*.^157^ (both baseline and longitudinal) |
| ONL | OCT | Yes | AD | Garcia-Martin *et al*.^118^; ↑Salobrar-Garcia *et al*.^131^; ↑Shao *et al*.^161^ |
|  |  |  | Brain Aβ+ | Santos *et al*.^157^ (longitudinal) |
|  |  | No | Brain Aβ+ | Snyder et al.^154^; Santos *et al*.^157^ (baseline) |
|  |  |  | MCI | Shin *et al*.^163^ |
|  |  |  | APOE ε4 carriers | López-Cuenca *et al*.^158^; Shin *et al*.^163^ |
| OPL | OCT | Yes | AD | Salobrar-Garcia *et al*.^131^; Sharma et al.^162^ |
|  |  |  | Brain Aβ+ | Santos *et al*.^157^ (both baseline and longitudinal) |
|  |  |  | APOE ε4 carriers | López-Cuenca *et al*.^158^ |
|  |  | No | AD | Garcia-Martin et al.^118^; |
|  |  |  | Brain Aβ+ | Snyder *et al*.^154^ |
|  |  |  | APOE ε4 carriers | Shin *et al*.^163^ |
|  |  |  | MCI | Shin *et al*.^163^ |
| RPE | OCT | Yes | AD | Sharma et al.^162^ |
|  |  | No | AD | Garcia-Martin *et al*.^118^; Salobrar-Garcia *et al*.^131^ |
|  |  |  | APOE ε4 carriers | López-Cuenca *et al*.^158^; Shin *et al*.^163^ |
|  |  |  | MCI | Shin *et al*.^163^ |
| Macular thickness | OCT | Yes | AD | Cunha *et al*.^115^; Iseri *et al*.^120^; Salobrar-Garcia *et al*.^131^ |
|  |  |  | Brain Aβ+ | Byun *et al*.^147^ |
|  |  |  | AD and MCI | Ascaso *et al*.^137^; Choi *et al*.^139^; Shao *et al*.^161^; Querques *et al*.^102^ |
|  |  | No | AD | Yan *et al*.^134^; Chi *et al*.^113^ |
|  |  |  | CSF biomarker + ve | Asanad *et al*.^146^ |
| Macular volume | OCT | Yes | AD | Cipollini *et al*.^114^; Iseri *et al*.^120^ |
|  |  |  | AD and MCI | Ascaso *et al*.^137^; Choi *et al*.^139^; Gao *et al*.^141^ |
|  |  | No | AD | Chi *et al*.^113^ |
|  |  |  | AD and MCI | Pillai *et al*.^151^ |
| Foveal thickness | OCT | Yes | AD | Cunha *et al*.^115^; Farzinvash *et al*.^166^; Salobrar-Garcia *et al*.^131^ |
|  |  |  | AD and MCI | Querques *et al*.^102^ |
|  |  |  | Brain Aβ+ | Byun *et al*.^147^ |
|  |  |  | APOE ε4 carriers | Ma *et al*.^109^ |
|  |  |  | PET and/or CSF biomarker +ve | O’Bryhim *et al*.^168^ |
|  |  | No | AD | Larrosa *et al*.^124^; Polo *et al*.^130^; Yan *et al*.^134^ |
| FAZ area | OCTA | Yes | AD | Bulut *et al*.^101^; Zabel *et al*.^135^; |
|  |  |  | MCI | Criscuolo *et al*.^136^ |
|  |  |  | AD and MCI | Wu *et al*.^169^ |
|  |  |  | PET and/or CSF biomarker +ve | O’Bryhim *et al*.^168^ |
|  |  |  | CSF Aβ42/tau ratio positive | Corradetti *et al*.^110^ |
|  | OCTA | No | AD | Lahme *et al*.^170^; Salobrar-Garcia *et al*.^105^ |
|  |  |  | AD and MCI | Chua *et al*.^171^; Wang *et al*.^172^; Yoon *et al*.^152^ |
|  |  |  | Brain Aβ+ | Haan *et al*.^103^; Van de Kreeke *et al*.^173^ |
|  |  |  | APOE ε4 carriers | Ma *et al*.^109^; Shin *et al*.^163^ |
|  |  |  | Cognitively impaired | Yang *et al*.^167^ |

Abbreviations: pRNFL, peripapillary retinal nerve fiber layer; OCT, optical coherence tomography; OCTA, optical coherence tomography angiography; AD, Alzheimer’s disease; MCI, mild cognitive impairment; Aβ+, brain amyloid positive; Aβ−, brain amyloid negative; CSF, cerebrospinal fluid; +ve, positive; APOE, apolipoprotein E; mRNFL, macular retinal nerve fiber layer; GCL, ganglion cell layer; GCL-IPL, ganglion cell layer and inner plexiform layer; RNFL-GCL-IPL, ganglion cell complex or GCC; IPL, inner plexiform layer; INL, inner nuclear layer; ONL, outer nuclear layer; OPL, outer plexiform layer; RPE, retinal pigment epithelium; FAZ, foveal avascular zone.

## **Supplementary Table S7**: Summary of studies evaluating the retinal vascular changes in different subjects obtained through different imaging methods.

| **Alterations** | **Imaging method** | **Significant difference between controls and cases** | **Subject** | **Study** |
| --- | --- | --- | --- | --- |
| Total retinal vascular density | OCTA | Yes | AD | Bulut at al.^101^ |
|  |  |  | Brain Aβ+ | Van de Kreeke *et al*.^173^ |
|  |  |  | AD and MCI | Jiang *et al*.^165^ |
|  |  |  | APOE ε4 carriers | Elahi *et al*.^174^ |
|  |  | No | Brain Aβ+ | Elahi *et al*.^174^; Haan *et al*.^103^ |
| Superficial capillary plexus microvascular density (SCP) | OCTA | Yes | AD | Chua *et al*.^171^; Jiang *et al*.^165^; Lahme *et al*.^170^; Yan *et al*.^134^ ; Zabel *et al*.^135^ |
|  |  |  | MCI | Chua *et al*.^171^; Criscuolo *et al*.^136^; Shin *et al*.^163^ |
|  |  |  | AD and MCI | Jianyang *et al*.^175^; Ma *et al*.^176^; Wang *et al*.^172^; Yoon *et al*.^152^ |
|  |  |  | CSF Aβ42/tau ratio positive | Corradetti *et al*.^110^ |
|  |  |  | Cognitively impaired | Yang *et al*.^167^ |
|  |  | No | APOE ε4 carriers | Shin *et al*.^163^; Ma *et al*.^109^ |
|  |  |  | MCI | Jiang *et al*.^165^ |
|  |  |  | AD and MCI | Wu *et al*.^169^; |
| Deep capillary plexus microvascular density (DCP) | OCTA | Yes | AD | Chua *et al*.^171^; Jiang *et al*.^165^; Zabel *et al*.^135^ |
|  |  |  | MCI | Criscuolo *et al*.^136^; Jiang *et al*.^165^ |
|  |  |  | Cognitively impaired | Yang *et al*.^167^ |
|  |  |  | APOE ε4 carriers | Shin *et al*.^163^ |
|  |  |  | AD and MCI | Jianyang *et al*.^175^; Wu *et al*.^169^ |
|  |  | No | AD | Lahme *et al*.^170^; Yan *et al*.^134^ |
|  |  |  | MCI | Chua *et al*.^171^; Shin *et al*.^163^ |
|  |  |  | AD and MCI | Wang *et al*.^172^ |
|  |  |  | CSF Aβ42/tau ratio positive | Corradetti *et al*.^110^ |
| Decreased blood flow | laser Doppler instrument | Yes | AD | Berisha *et al*.^112^ |
|  |  |  | AD and MCI | Feke *et al*.^150^ |
|  | OCTA | No | AD | Bulut *et al*.^101^ |
| Reduced perfusion density | OCTA | Yes | AD and MCI | Ma *et al*.^176^; Yoon *et al*.^152^ |
|  |  |  | CSF Aβ42/tau ratio positive | Corradetti *et al*.^110^ |
|  |  | No | APOE ε4 carriers | Ma *et al*.^109^ |
|  |  |  | AD and MCI | Chua *et al*.^171^; Querques *et al*.^102^; |
| Altered microvascular network | OCTA | Yes | AD and MCI | Chua *et al*.^171^; Jianyang *et al*.^175^ |
|  |  |  | CSF Aβ42/tau ratio positive | Corradetti *et al*.^110^ |
|  | SLO | Yes | AD and MCI | Pead *et al*.^177^ |
|  |  |  | Cognitively impaired | Cabrera DeBuc *et al*.^178^ |
|  | Fundus photography | Yes | AD | Cheung *et al*. (fractals)^179^; Frost *et al*. (fractals; vascular branching coefficient and vascular asymmetry)^180^; Williams *et al*. (fractals)^181^ |
|  |  |  | Brain Aβ+ | Haan *et al*.^103^ (fractals - arterioles) |
|  |  | No | AD | Cheung *et al*. (vascular bifurcation)^179^; Williams *et al*. (vascular bifurcation)^181^ |
|  |  |  | Brain Aβ+ | Haan *et al*.^103^ (fractals - venules) |
| Oxygen saturation of haemoglobin | spectrophotometric retinal oximeter | Yes | AD | Einarsdottir *et al*.^182^ |
| Venule diameter changes | laser Doppler instrument | Yes | AD | Berisha *et al*.^112^ |
|  |  |  | AD and MCI | Feke *et al*.^150^ |
|  | SLO | Yes | AD and MCI | Pead *et al*.^177^ |
|  |  |  | AD | Csincsik 2018^183^ |
|  |  | No | Cognitively impaired | Cabrera DeBuc *et al*.^178^ |
|  | Fundus photography | Yes | AD | Cheung *et al*.^179^; Frost *et al*.^180^ |
|  |  | No | AD | Jong *et al*^184^ |
| increased vascular tortuosity | Fundus photography | Yes | AD | Cheung *et al*.^179^ |
|  |  | No | Brain Aβ+ | Haan *et al*.^103^ |
|  | Hyperspectral imaging | Yes | Brain Aβ+ | Sharafi *et al*.^185^ |
|  | SLO | No | AD and MCI | Pead *et al*.^177^; Csincsik 2018^183^ |
|  |  |  | Cognitively impaired | Cabrera DeBuc *et al*.^178^; |
| reduced vascular tortuosity | Fundus photography | Yes | AD | Frost *et al*.^180^; Williams *et al*.^181^ |
| Artery diameter changes | Fundus photography | Yes | AD | Williams *et al*.^181^ |
|  | SLO | No | AD | Csincsik 2018^183^ |
|  |  |  | Cognitively impaired | Cabrera DeBuc *et al*.^178^; |
|  | Hyperspectral imaging | Yes | Brain Aβ+ | Sharafi *et al*.^185^ |

Abbreviations: OCTA, optical coherence tomography angiography; AD, Alzheimer’s disease; MCI, mild cognitive impairment; +ve, positive; Aβ+, brain amyloid positive; Aβ−, brain amyloid negative; APOE, apolipoprotein E; SLO, scanning laser ophthalmoscope.

## **Supplementary Table S8**: Summary of AD-related ONH changes reported across different studies.

| **Study** | **Method** | **Sample Size** | **Significantly different parameters** | **Non-significant** |
| --- | --- | --- | --- | --- |
| Tsai *et al*. (1991)^186^ | Fundus photography and ONH analysis | Fundus imaging: AD = 30, HC = 32; ONH Topography analysis: AD = 26, HC = 36 | ↑Cup-to-disc ratio and cup volume, ↓disc rim area in AD compared to HC. | Pallor area-to-disc area ratio between AD and HC; however, patients with higher pallor area-to-disc area ratios had higher Alzheimer's Disease Assessment Scale. |
| Danesh-Meyer et al. (2006)^159^ | SLO imaging of retina | HC = 40; AD = 50 | ↑Cup/disc area ratio; ↓Rim area, and↓ Cup volume |  |
| Kurna et al., (2014)^160^ | SLO imaging of retina | HC = 25; AD = 24 | NA | Rim area, rim volume, height variation contour, linear cup/disc ratio, and cup shape measures |
| Bambo *et al*. (2015)^111^ | SD-OCT | AD = 56, HC = 56 | ↓Hemoglobin amount in optic disk calculated using Laguna ONhE, ↑paler ONH | NA |
| Cesareo *et al*. (2015)^65^ | SLO (Heidelberg Retinal Tomograph III) | AD = 51, HC = 67 | NA | ↑Cup Shape Measure (ONH) and ↑Vertical cup/disc ratio (ONH) |
| Zabel et al., (2019)^135^ | SD-OCT | AD = 27; HC = 27 | NA | Disc area, Rim area; Cup/disc area ratio; Cup volume |
| JA Van de Kreeke *et al*. (2020)^173^ | OCTA | Aβ+ = 13, Aβ− = 111 | ↑Retinal vessel density around ONH | NA |
| Salobrar-Garcia *et al*. (2020)^105^ | SD-OCT, OCTA, Laguna ONhE program | AD =17, HC = 49 | NA | ONH hemoglobin values between AD and control groups |
| Chaitanuwong *et al*. (2023)^187^ | OCTA | AD = 24, HC = 39 | ↓Optic disc vessel density at the nerve head level, and ↓optic disc vessel density at the radial peripapillary capillary level | NA |

Abbreviations: AD, Alzheimer’s disease; MCI, mild cognitive impairment; HC, healthy controls; ONH, optic nerve head; Aβ+, amyloid beta positive; Aβ−, amyloid beta negative; OCT, optical coherence tomography; SD-OCT, spectral domain OCT; OCTA, optical coherence tomography angiography; NA, not available; SLO, scanning laser ophthalmoscope.

## **Supplementary Table S9**: Summary of AD-associated hyperspectral retinal imaging studies on animal and human models.

| **Research** | **Scanning system** | **Study method** | **Sample size** | **Major findings** |
| --- | --- | --- | --- | --- |
| More *et al*. (2015)^32^ | Hyperspectral  dark field microscope (400 – 1000 nm) | -Imaging of cell culture (SH-SY5Y human neuroblastic cells), -Ex vivo imaging of transgenic Alzheimer’s (APP/PS1) and wild type mice brain and retina. | APP/PS1 mice = 10 (females) with anti-Alzheimer’s drug and for vehicle control = 10 (females); wild type mice = 10 (females) | - The mouse model revealed that optical property anomalies of the retina were associated with AD pathology, which correlated with AD progression and were present before the formation of retinal plaques, -The spectral Aβ signature consistent with variations in Rayleigh light-scattering, indicating the influence of low-order Aβ1-42 accumulation in the retina ranging from soluble colloidal form to insoluble plaques. |
| More *et al*. (2016)^188^ | Topical endoscopic fundal imaging (TEFI) camera (400 – 800 nm) | -In vivo imaging of transgenic Alzheimer’s (APP/PS1) and wild type mice retina. | APP/PS1 mice = 8; wild type mice = 8 | - Rayleigh light scattering changes detected in mouse retinas were due to progressive increase in soluble amyloid aggregates over six months, with a significant accumulation at seven months. |
| More *et al*. (2019)^189^ | Custom built imaging system (400 – 1000 nm) | -In vivo retinal imaging. | - AD = 19 and HC = 16. | The rHSI signature may have a higher sensitivity in the initial stages of AD patients with a higher MMSE score (22-26 out of 30). |
| Sharafi *et al*. (2019)^185^ | MHRC, Optina Diagnostics, Montreal, Canada (450-905 nm) | -In vivo imaging of brain Aβ positive, MCI and age-matched HCs | Brain Aβ+ = 16; brain Aβ− = 30 | - A significant difference in spatial/spectral texture measures developed across retinal arterioles and surrounding tissues between Aβ+ and Aβ− individuals, - Aβ+ subjects had more tortuous retinal venules and larger arteriolar diameters than Aβ− subjects. |
| Hadoux *et al*. (2019)^190^ | MHRC, Optina Diagnostics, Montreal, Canada (450-905 nm) | -In vivo retinal imaging of brain Aβ positive individuals and age-matched HCs  - In vivo retinal imaging of 5×FAD mice and age-matched wild type mice. | - Brain Aβ positive individuals = 15 and HC = 20, - 5×FAD mice = 12 and wild type mice = 10. | - Hyperspectral retinal scores calculated using a machine learning approach were higher for participants with higher brain amyloid load compared to age-matched HCs as determined by PET. - Similar differences were found in the retinal reflectance of 9 to 14 months 5×FAD mice compared to their age-matched HCs. |
| Lemmens *et al*., (2020)^191^ | Hyperspectral snapshot camera (460 – 420 nm) | -In vivo retinal imaging of AD participants and HCs. | AD = 17; HC = 22 | rHSI and OCT, in conjunction with a machine learning technique, can improve the accuracy of a hyperspectral imaging-based model for AD detection and classification. |
| Lim *et al*. (2021)^192^ | Custom-built bench ophthalmoscope (380 – 680 nm) | -*In-vitro* imaging of 1 mg purified human Aβ42 solution, -In vivo imaging of transgenic 5×FAD and wild type control mice. | 5×FAD transgenic mice = 9 (6 months), 9 (12 months) and 8 (17 months); wild type mice = 12 (6 months), 13 (12 months) and 15 (17 months) | - The reflectance at longer wavelengths was observed to increase with the age of mice, indicating an increase in Aβ deposition in the retina, -The *in-vitro* hyperspectral imaging of the Aβ solution showed a higher reflectance than the vehicle control, with the differences more observable in the short visible spectrum (<500 nm). |
| Vandena-beele *et al*. (2021)^30^ | Hyperspectral visible and near infrared snap scan camera (470-900 nm) | -In vivo imaging of AppNL-G-F and APP/PS1 AD and wild type control mice. | - AppNL-G-F mice = 9 (3 months) and 10 (18 months); wild type mice = 7 (3 months) and 8 (18 months)  - APP/PS1 AD mice = 4 (18 months); wild type mice = 8 (18 months) | - Average hyperspectral scores of 3-months-old AppNL-G-F and wild type mice were significantly different. - The hyperspectral scores (HS) of retinal amyloid burdens were significantly higher for AppNL-G-F and APP/PS1 AD mice compared to the age-matched wild-type mice at the age of 18 months. |
| Du *et al*., (2022)^37^ | Custom-built hyperspectral imaging system (420 - 720nm) | -Ex vivo hyperspectral imaging, immunostaining and analyzing the amyloid and pTau load of retina using deep network. | - For histological examination: 12 retinas from AD (n=2), MCI (n=3), and HC(n=5). -Additional retinas from AD cases (n=3) were employed for histological and rHSI analyses, followed by network training. | -Detection of spectral signature for pTau. - Development of label free prediction model of the retinal Aβ and pTau distribution using the deep learning method. |
| Poudel *et al.* (2024)^193^ | MHRC, Optina Diagnostics, Montreal, Canada (450-905 nm) | In vivo imaging of individuals with various brain Aβ loads | Aβ+ = 32; Aβ− = 34 | - A Significant difference in the reflectance spectra of individuals with increasing brain amyloid loads in 450-585 nm wavelengths.  - Retinal features obtained in the superior view of the right eye showed higher inter-subject variability.  -The classification model developed using spectral features showed 0.758–0.879 accuracy, 0.718–0.909 sensitivity, 0.764–0.912 specificity, and 0.745–0.891 area under curve for the right eye. |

Abbreviations: AD, Alzheimer’s disease; MCI, mild cognitive impairment; HC, healthy controls, MHRC, metabolic hyperspectral retinal camera; PET, positron emission tomography; OCT, optical coherence tomography; rHSI, retinal hyperspectral imaging; Aβ+, amyloid beta positive; Aβ−, amyloid beta negative; nm, nanometer.

## **Supplementary Table S10**: Summary of AD studies evaluating the performance of different AI models for the discrimination of cases and controls using non-invasive imaging methods.

| **Publication** | **AI Technique** | **Sample Size** | **Model Performance** |
| --- | --- | --- | --- |
| Nunes et al. (2019)^194^ | SVM ML method | Fundus images of AD = 20 and HC = 27 | A median sensitivity of 79.5% for the clinical class classification of AD from HC |
| Sharafi et al. (2019)^185^ | PCA and SVM ML methods | Hyperspectral images of Aβ + = 16 and Aβ− = 30 | Accuracy of 85% to differentiate Aβ+ cases from Aβ− subjects. |
| Hadoux et al. (2019)^190^ | DROP-D ML method | Hyperspectral images of Aβ+ = 15 and Aβ− = 20 | 0.87 (0.69–1.0) validation AUC to differentiate Aβ+ cases from Aβ− subjects. |
| Lemmens et al. (2020)^191^ | LDA ML method | Hyperspectral images of AD = 17 and HC = 20 | AUC of 0.74 to discriminate probable AD cases from HC |
| Wisely et al. (2020)^195^ | DL method | Color map of GCL-IPL thickness and SCP images from OCTA and FAF images from SLO of AD = 36 (62 eyes) and HC = 123 (222 eyes) | Identification of AD from HC with an AUC of 0.841 for GC-IPL thickness and patient clinical data. |
| Zhang et el. (2021)^196^ | ELM DL and SVM ML models | 332 vascular-related features extracted from fundus images of AD = 22, MCI = 26 and HC = 38 | Classification performance of the SVM model for the control group, MCI group, and dementia group were 0.85, 0.81, and 0.81 AUCs, respectively |
| Tian et al. (2021)^197^ | SVM ML method | Vessels map from 244 quality fundus images of AD = 87 and HC = 87 | Classification accuracy of 82.44% for the clinical class classification of AD from HC |
| Kim et al. (2022)^198^ | DL method | 85,711 retinal fundus images of AD = 111, HC = 111 | 0.929 area under curve (AUC), 0.837 Sensitivity, and 0.891 specificity |
| Cheung et al. (2022)^199^ | DL method | 12,949 fundus images of AD = 648 and HC = 3240 | The DL model achieved accuracies ranging from 79·6% (±15·5% (SD)) to 92·1% (± 11·4% (SD)) and ROCs ranging from 0·73 (± 0·24 (SD)) to 0·91 (±0·10 (SD)) for discrimination of patients with AD from those without. In the datasets with brain amyloid PET scores, the model had accuracies ranged from 80·6% (±13·4% (SD)) to 89·3% (±13·7% (SD)), and ROCs ranged from 0·68 (±0·24 (SD)) to 0·86 (±0·16 (SD)) to differentiate amyloid β positive from negative individuals. |
| Corbin et al. (2022)^200^ | DL method | 25,737 fundus images of 14711 participants with different cognitive abilities | variance explained by the model of 22.4% for the prediction of the global cognition of the participants. |
| Poudel et al. (2024)^193^ | Integrated PCA and LDA method | Hyperspectral images of Aβ+ = 32 and Aβ− = 34 (cutoff = 1.35 SUVR); cases = 18, controls = 48 (cutoff = 10 Centiloid); cases = 11, controls = 55 (cutoff = 20 Centiloid) | AUC of 0.847 to differentiate Aβ+ cases from Aβ− subjects with cutoff value of 1.35 SUVR; AUC of 0.823 to differentiate cases from controls with cutoff value of 10 Centiloid and AUC of 0.891 to differentiate cases from controls with cutoff value of 20 Centiloid. |

Abbreviations: AI, artificial intelligence; ML, machine learning; DL, deep learning; ELM, extreme learning machine; SVM, support vector machine; PCA, principal component analysis; LDA, linear discrimination analysis; DROP-D, Dimension reduction by orthogonal projection for discrimination; AD, Alzheimer’s disease; MCI, mild cognitive impairment; HC, healthy control; AUC, area under curve; SD, standard deviation; GC-IPL, ganglion cell layer and inner plexiform layer; FAF, fundus autofluorescence; SLO, scanning laser ophthalmoscope; Aβ+, amyloid beta positive; Aβ−, amyloid beta negative; PET, positron emission tomography; AUC, area under the curve; SUVR, Standardized Uptake Value Ratio.

## **Supplementary Table S11**: Comparative analysis of ocular biomarkers for AD detection

| **Ocular Biomarker** | **Ease of Measurement** | **Device Availability** | **Cost-Effectiveness** | **Time Efficiency** | **Consistency of Findings** | **Volume of Literature** | **Potential for Widespread Screening** |
| --- | --- | --- | --- | --- | --- | --- | --- |
| **Pupillary Response** | Moderate (requires standardised image acquisition methods) | Limited; (pupillometry devices needed) | Moderate (not widely available in clinics) | Fast (few seconds) | Moderate (some conflicting findings) | Moderate (more studies on large sample size are needed) | High (portable, potential for mass screening) |
| **Lens (Cataract/Aβ Deposits)** | Low (requires specialized autofluorescence imaging) | Limited; (primarily in research) | Expensive (Specialized imaging techniques needed) | Fast (few seconds) | Low (few studies, limited replication) | Low (more studies on large sample size are needed) | Low (not widely available) |
| **Corneal Changes (Nerve Fiber Density, Length, Sensitivity)** | Moderate (requires corneal confocal microscopy) | Limited; (high-end research devices required) | Expensive (high cost for specialized imaging) | Low (Time-consuming) | Moderate (inconsistent in pre-clinical participants) | Moderate (More studies needed with pre-clinical participants) | Moderate (requires operator expertise) |
| **Eye Movement (Saccades, Fixation, Pursuit Tracking)** | Moderate (requires eye-tracking technology) | Limited; (not common in clinical settings) | Expensive (Specialized imaging techniques needed) | Moderate (repeated measurements required) | Moderate (growing evidence in clinical stages) | Moderate (More studies needed with pre-clinical participants) | Moderate (requires specialized setup) |
| **Choroidal Changes (Thickness, Area, Luminal Area, Vascularity Index, Blood Flow)** | Challenging (requires enhanced depth imaging-OCT or SS-OCT) | Available mainly in research settings | Expensive | Moderate (high depth and quality image needed) | Moderate (some variability in results) | Moderate | Low (specialized imaging technique and image processing method required) |
| **Retinal Structural and Vascular Changes (SLO/OCT/A)** | Easy (non-invasive, automated, widely used in clinics) | Widely available in ophthalmology clinics and hospitals | Cost-effective compared to neuroimaging | Moderate (a few minutes to get quality images) | High (higher consistent findings on RNFL thinning, vascular alterations) | High (strong correlation with AD pathology) | High (can be implemented in routine eye exams) |
| **Retinal Fundus Photography** | Easy (widely used, non-invasive) | Commonly available in clinical and research settings | Low-cost (standard in eye exams) | Fast (a seconds to min per scan) | Moderate to high (some promising findings on retinal vascular changes, drusen, and Aβ deposition) | Moderate (varies by parameter analyzed) | High (widely available, potential for primary care and screening) |
| **Hyperspectral Imaging** | Difficult (requires specialized imaging and analysis method) | Very limited; mostly in research | Expensive (tunable laser source device is required) | Moderate (needs no eye movement and blinking during scanning) | Low (limited studies, still experimental) | Moderate (recently studies expanding with comparable effect size) | Moderate (not available for clinical use, operator expertise required) |

Abbreviations: OCT, optical coherence tomography; OCTA, optical coherence tomography angiography; SLO, scanning laser ophthalmoscope; SS-OCT, swept source OCT.

# Supplementary references

1. Lim JKH, Li Q-X, He Z, et al. The Eye As a Biomarker for Alzheimer's Disease. Review. *Frontiers in Neuroscience*. 2016-November-17 2016;10

2. Guerrero-Moreno A, Baudouin C, Melik Parsadaniantz S, Réaux-Le Goazigo A. Morphological and Functional Changes of Corneal Nerves and Their Contribution to Peripheral and Central Sensory Abnormalities. *Frontiers in cellular neuroscience*. 2020;14:610342-610342.

3. Opwonya J, Doan DNT, Kim SG, et al. Saccadic Eye Movement in Mild Cognitive Impairment and Alzheimer’s Disease: A Systematic Review and Meta-Analysis. *Neuropsychology Review*. 2022/06/01 2022;32(2):193-227.

4. Mahabadi N, Al Khalili Y. Neuroanatomy, Retina. *StatPearls*. 2023.

5. Straatsma BR, Foos RY, Heckenlively JR, Taylor GN. Myelinated retinal nerve fibers. *Am J Ophthalmol*. Jan 1981;91(1):25-38.

6. Smith AM, Czyz CN. Neuroanatomy, Cranial Nerve 2 (Optic). *StatPearls*. StatPearls Publishing

Copyright © 2025, StatPearls Publishing LLC.; 2025.

7. London A, Benhar I, Schwartz M. The retina as a window to the brain-from eye research to CNS disorders. *Nat Rev Neurol*. Jan 2013;9(1):44-53.

8. Le A, Shin A, Park J, Poukens V, Demer JL. Bilaminar Structure of the Human Optic Nerve Sheath. *Curr Eye Res*. Jul 2020;45(7):864-872.

9. Frost S, Martins RN, Kanagasingam Y. Ocular biomarkers for early detection of Alzheimer's disease. *J Alzheimers Dis*. 2010;22(1):1-16.

10. Hussain A, Sheikh Z, Subramanian M. The Eye as a Diagnostic Tool for Alzheimer&rsquo;s Disease. *Life*. 2023;13(3):726.

11. McKhann G, Drachman D, Folstein M, Katzman R, Price D, Stadlan EM. Clinical diagnosis of Alzheimer's disease: Report of the NINCDS-ADRDA Work Group* under the auspices of Department of Health and Human Services Task Force on Alzheimer's Disease. *Neurology*. 1984;34(7):939-939.

12. Koronyo-Hamaoui M, Koronyo Y, Ljubimov AV, et al. Identification of amyloid plaques in retinas from Alzheimer's patients and noninvasive in vivo optical imaging of retinal plaques in a mouse model. *NeuroImage*. 2011;54 Suppl 1:S204-S217.

13. Koronyo Y, Biggs D, Barron E, et al. Retinal amyloid pathology and proof-of-concept imaging trial in Alzheimer’s disease. *JCI Insight*. 2017;2(16)

14. Koronyo Y, Rentsendorj A, Mirzaei N, et al. Retinal pathological features and proteome signatures of Alzheimer’s disease. *Acta Neuropathologica*. 2023/04/01 2023;145(4):409-438.

15. La Morgia C, Ross-Cisneros FN, Koronyo Y, et al. Melanopsin retinal ganglion cell loss in Alzheimer disease. *Ann Neurol*. Jan 2016;79(1):90-109.

16. Grimaldi A, Pediconi N, Oieni F, et al. Neuroinflammatory Processes, A1 Astrocyte Activation and Protein Aggregation in the Retina of Alzheimer’s Disease Patients, Possible Biomarkers for Early Diagnosis. Original Research. *Frontiers in Neuroscience*. 2019-September-04 2019;13

17. Shi H, Koronyo Y, Rentsendorj A, et al. Identification of early pericyte loss and vascular amyloidosis in Alzheimer’s disease retina. *Acta Neuropathologica*. 2020/05/01 2020;139(5):813-836.

18. Xu QA, Boerkoel P, Hirsch-Reinshagen V, et al. Müller cell degeneration and microglial dysfunction in the Alzheimer’s retina. *Acta Neuropathologica Communications*. 2022/10/05 2022;10(1):145.

19. Tsai Y, Lu B, Ljubimov AV, et al. Ocular changes in TgF344-AD rat model of Alzheimer's disease. *Investigative ophthalmology & visual science*. 2014;55(1):523-534.

20. Lee S, Jiang K, McIlmoyle B, et al. Amyloid Beta Immunoreactivity in the Retinal Ganglion Cell Layer of the Alzheimer’s Eye. Original Research. *Frontiers in Neuroscience*. 2020-July-31 2020;Volume 14 - 2020

21. Alexandrov PN, Pogue A, Bhattacharjee S, Lukiw WJ. Retinal amyloid peptides and complement factor H in transgenic models of Alzheimer's disease. *Neuroreport*. 2011;22(12):623-627.

22. Schultz N, Byman E, Netherlands Brain B, Wennström M. Levels of Retinal Amyloid-β Correlate with Levels of Retinal IAPP and Hippocampal Amyloid-β in Neuropathologically Evaluated Individuals. *Journal of Alzheimer's disease : JAD*. 2020;73(3):1201-1209.

23. den Haan J, Morrema THJ, Verbraak FD, et al. Amyloid-beta and phosphorylated tau in post-mortem Alzheimer's disease retinas. *Acta neuropathologica communications*. 2018;6(1):147-147.

24. Ho C-Y, Troncoso JC, Knox D, Stark W, Eberhart CG. Beta-amyloid, phospho-tau and alpha-synuclein deposits similar to those in the brain are not identified in the eyes of Alzheimer's and Parkinson's disease patients. *Brain pathology (Zurich, Switzerland)*. 2014;24(1):25-32.

25. Schön C, Hoffmann NA, Ochs SM, et al. Long-term in vivo imaging of fibrillar tau in the retina of P301S transgenic mice. *PLoS One*. 2012;7(12):e53547.

26. Williams EA, McGuone D, Frosch MP, Hyman BT, Laver N, Stemmer-Rachamimov A. Absence of Alzheimer Disease Neuropathologic Changes in Eyes of Subjects With Alzheimer Disease. *Journal of neuropathology and experimental neurology*. 2017;76(5):376-383.

27. Ma Q, Zhao Z, Sagare AP, et al. Blood-brain barrier-associated pericytes internalize and clear aggregated amyloid-β42 by LRP1-dependent apolipoprotein E isoform-specific mechanism. *Molecular Neurodegeneration*. 2018/10/19 2018;13(1):57.

28. Shi H, Koronyo Y, Fuchs D-T, et al. Retinal capillary degeneration and blood-retinal barrier disruption in murine models of Alzheimer’s disease. *Acta Neuropathologica Communications*. 2020/11/23 2020;8(1):202.

29. Habiba U, Merlin S, Lim JKH, et al. Age-Specific Retinal and Cerebral Immunodetection of Amyloid-β Plaques and Oligomers in a Rodent Model of Alzheimer's Disease. *J Alzheimers Dis*. 2020;76(3):1135-1150.

30. Vandenabeele M, Veys L, Lemmens S, et al. The AppNL-G-F mouse retina is a site for preclinical Alzheimer’s disease diagnosis and research. *Acta Neuropathologica Communications*. 2021/01/06 2021;9(1):6.

31. Grimaldi A, Brighi C, Peruzzi G, et al. Inflammation, neurodegeneration and protein aggregation in the retina as ocular biomarkers for Alzheimer’s disease in the 3xTg-AD mouse model. *Cell Death &amp; Disease*. 2018;9(6)

32. More SS, Vince R. Hyperspectral imaging signatures detect amyloidopathy in Alzheimer's mouse retina well before onset of cognitive decline. *ACS Chem Neurosci*. Feb 18 2015;6(2):306-15.

33. Lukiw WJ, Zhao Y, Cui JG. An NF-kappaB-sensitive micro RNA-146a-mediated inflammatory circuit in Alzheimer disease and in stressed human brain cells. *J Biol Chem*. Nov 14 2008;283(46):31315-22.

34. Chiasseu M, Alarcon-Martinez L, Belforte N, et al. Tau accumulation in the retina promotes early neuronal dysfunction and precedes brain pathology in a mouse model of Alzheimer’s disease. *Molecular Neurodegeneration*. 2017;12(1)

35. Walkiewicz G, Ronisz A, Van Ginderdeuren R, et al. Primary retinal tauopathy: A tauopathy with a distinct molecular pattern. *Alzheimer's & Dementia*. 2024;20(1):330-340.

36. Shi H, Mirzaei N, Koronyo Y, et al. Identification of retinal oligomeric, citrullinated, and other tau isoforms in early and advanced AD and relations to disease status. *Acta Neuropathologica*. 2024/07/09 2024;148(1):3.

37. Du X, Koronyo Y, Mirzaei N, et al. Label-Free Hyperspectral Imaging and Deep-Learning Prediction of Retinal Amyloid β-Protein and Phosphorylated Tau. *bioRxiv*. 2022:2022.06.03.494650.

38. Hart de Ruyter FJ, Morrema THJ, den Haan J, et al. Phosphorylated tau in the retina correlates with tau pathology in the brain in Alzheimer’s disease and primary tauopathies. *Acta Neuropathologica*. 2023/02/01 2023;145(2):197-218.

39. Prettyman R, Bitsios P, Szabadi E. Altered pupillary size and darkness and light reflexes in Alzheimer's disease. *J Neurol Neurosurg Psychiatry*. Jun 1997;62(6):665-8.

40. Fotiou F, Fountoulakis KN, Tsolaki M, Goulas A, Palikaras A. Changes in pupil reaction to light in Alzheimer’s disease patients: a preliminary report. *International Journal of Psychophysiology*. 2000/07/01/ 2000;37(1):111-120.

41. Granholm E, Morris S, Galasko D, Shults C, Rogers E, Vukov B. Tropicamide effects on pupil size and pupillary light reflexes in Alzheimer's and Parkinson's disease. *Int J Psychophysiol*. Feb 2003;47(2):95-115.

42. Fotiou DF, Brozou CG, Haidich AB, et al. Pupil reaction to light in Alzheimer's disease: evaluation of pupil size changes and mobility. *Aging Clin Exp Res*. Oct 2007;19(5):364-71.

43. Frost S, Kanagasingam Y, Sohrabi H, et al. Pupil response biomarkers for early detection and monitoring of Alzheimer's disease. *Curr Alzheimer Res*. Nov 2013;10(9):931-9.

44. Frost SM, Kanagasingam Y, Sohrabi HR, et al. Pupil response biomarkers distinguish amyloid precursor protein mutation carriers from non-carriers. *Curr Alzheimer Res*. Oct 2013;10(8):790-6.

45. Bittner DM, Wieseler I, Wilhelm H, Riepe MW, Müller NG. Repetitive pupil light reflex: potential marker in Alzheimer's disease? *J Alzheimers Dis*. 2014;42(4):1469-77.

46. Frost S, Robinson L, Rowe CC, et al. Evaluation of Cholinergic Deficiency in Preclinical Alzheimer's Disease Using Pupillometry. *Journal of Ophthalmology*. 2017;2017

47. Kremen WS, Panizzon MS, Elman JA, et al. Pupillary dilation responses as a midlife indicator of risk for Alzheimer's disease: association with Alzheimer's disease polygenic risk. *Neurobiology of Aging*. 2019/11/01/ 2019;83:114-121.

48. Oh AJ, Amore G, Sultan W, et al. Pupillometry evaluation of melanopsin retinal ganglion cell function and sleep-wake activity in pre-symptomatic Alzheimer’s disease. *PLOS ONE*. 2019;14(12):e0226197.

49. Kawasaki A, Ouanes S, Crippa SV, Popp J. Early-stage Alzheimer’s disease does not alter pupil responses to colored light stimuli. *Journal of Alzheimer's Disease*. 2020;75(4):1273-1282.

50. El Haj M, Chapelet G, Moustafa AA, Boutoleau-Bretonnière C. Pupil size as an indicator of cognitive activity in mild Alzheimer's disease. *Excli j*. 2022;21:307-316.

51. Gramkow MH, Clemmensen FK, Sjælland NS, Waldemar G, Hasselbalch SG, Frederiksen KS. Diagnostic performance of light reflex pupillometry in Alzheimer's disease. *Alzheimers Dement (Amst)*. Jul-Sep 2024;16(3):e12628.

52. Opwonya J, Kim K, Lee KH, Kim JI, Kim JU. Task-evoked pupillary responses as potential biomarkers of mild cognitive impairment. *Alzheimer's & Dementia: Diagnosis, Assessment & Disease Monitoring*. 2024;16(4):e70019.

53. Frederikse PH, Garland D, Zigler JS, Jr., Piatigorsky J. Oxidative stress increases production of beta-amyloid precursor protein and beta-amyloid (Abeta) in mammalian lenses, and Abeta has toxic effects on lens epithelial cells. *J Biol Chem*. Apr 26 1996;271(17):10169-74.

54. Goldstein LE, Muffat JA, Cherny RA, et al. Cytosolic β-amyloid deposition and supranuclear cataracts in lenses from people with Alzheimer's disease. *The Lancet*. 2003/04/12/ 2003;361(9365):1258-1265.

55. Moncaster JA, Pineda R, Moir RD, et al. Alzheimer's Disease Amyloid-β Links Lens and Brain Pathology in Down Syndrome. *PLoS ONE*. 2010;5(5):e10659.

56. Kerbage C, Sadowsky CH, Jennings D, Cagle GD, Hartung PD. Alzheimer's disease diagnosis by detecting exogenous fluorescent signal of ligand bound to Beta amyloid in the lens of human eye: an exploratory study. *Front Neurol*. 2013;4:62.

57. Michael R, Rosandić J, Montenegro GA, et al. Absence of beta-amyloid in cortical cataracts of donors with and without Alzheimer's disease. *Exp Eye Res*. Jan 2013;106:5-13.

58. Michael R, Otto C, Lenferink A, et al. Absence of amyloid-beta in lenses of Alzheimer patients: a confocal Raman microspectroscopic study. *Exp Eye Res*. Feb 2014;119:44-53.

59. Kerbage C, Sadowsky CH, Tariot PN, et al. Detection of Amyloid β Signature in the Lens and Its Correlation in the Brain to Aid in the Diagnosis of Alzheimer’s Disease. *American Journal of Alzheimer's Disease &amp; Other Dementiasr*. 2015;30(8):738-745.

60. Bei L, Shui YB, Bai F, Nelson SK, Van Stavern GP, Beebe DC. A test of lens opacity as an indicator of preclinical Alzheimer Disease. *Exp Eye Res*. Nov 2015;140:117-123.

61. Moncaster JA, Moir RD, Burton MA, et al. Alzheimer's disease amyloid-β pathology in the lens of the eye. *Experimental Eye Research*. 2022/08/01/ 2022;221:108974.

62. Frederikse PH, Zigler JS, Jr. Presenilin expression in the ocular lens. *Curr Eye Res*. Sep 1998;17(9):947-52.

63. Dutescu RM, Li QX, Crowston J, Masters CL, Baird PN, Culvenor JG. Amyloid precursor protein processing and retinal pathology in mouse models of Alzheimer's disease. *Graefes Arch Clin Exp Ophthalmol*. Sep 2009;247(9):1213-21.

64. Örnek N, Dağ E, Örnek K. Corneal sensitivity and tear function in neurodegenerative diseases. *Curr Eye Res*. Apr 2015;40(4):423-8.

65. Cesareo M, Martucci A, Ciuffoletti E, et al. Association Between Alzheimer's Disease and Glaucoma: A Study Based on Heidelberg Retinal Tomography and Frequency Doubling Technology Perimetry. *Front Neurosci*. 2015;9:479.

66. Choi S-i, Lee B, Woo JH, Jeong JB, Jun I, Kim EK. APP processing and metabolism in corneal fibroblasts and epithelium as a potential biomarker for Alzheimer's disease. *Experimental Eye Research*. 2019/05/01/ 2019;182:167-174.

67. Ponirakis G, Al Hamad H, Sankaranarayanan A, et al. Association of corneal nerve fiber measures with cognitive function in dementia. *Ann Clin Transl Neurol*. Apr 2019;6(4):689-697.

68. Dehghani C, Frost S, Jayasena R, et al. Morphometric Changes to Corneal Dendritic Cells in Individuals With Mild Cognitive Impairment. Original Research. *Frontiers in Neuroscience*. 2020-December-09 2020;14

69. Al-Janahi E, Ponirakis G, Al Hamad H, et al. Corneal nerve and brain imaging in mild cognitive impairment and dementia. *Journal of Alzheimer's Disease*. 2020;77(4):1533-1543.

70. Ponirakis G, Hamad HA, Khan A, et al. Loss of corneal nerves and brain volume in mild cognitive impairment and dementia. *Alzheimer's & Dementia: Translational Research & Clinical Interventions*. 2022;8(1):e12269.

71. Ponirakis G, Al Hamad H, Omar DAM, et al. Corneal nerve loss predicts dementia in patients with mild cognitive impairment. *Ann Clin Transl Neurol*. Apr 2023;10(4):599-609.

72. Gundogan AO, Oltulu R, Belviranli S, et al. Corneal innervation changes ın Alzheimer’s: implications for sensory dysfunction. *International Ophthalmology*. 2024/06/24 2024;44(1):270.

73. Alichniewicz K, Brunner F, Klünemann H, Greenlee M. Neural correlates of saccadic inhibition in healthy elderly and patients with amnestic mild cognitive impairment. Original Research. *Frontiers in Psychology*. 2013-July-24 2013;4

74. Boucart M, Calais G, Lenoble Q, Moroni C, Pasquier F. Differential processing of natural scenes in posterior cortical atrophy and in Alzheimer’s disease, as measured with a saccade choice task. Original Research. *Frontiers in Integrative Neuroscience*. 2014-July-25 2014;8

75. Boxer AL, Garbutt S, Rankin KP, et al. Medial versus lateral frontal lobe contributions to voluntary saccade control as revealed by the study of patients with frontal lobe degeneration. *J Neurosci*. Jun 7 2006;26(23):6354-63.

76. Boxer AL, Garbutt S, Seeley WW, et al. Saccade Abnormalities in Autopsy-Confirmed Frontotemporal Lobar Degeneration and Alzheimer Disease. *Archives of Neurology*. 2012;69(4):509-517.

77. Chehrehnegar N, Nejati V, Shati M, et al. Behavioral and cognitive markers of mild cognitive impairment: diagnostic value of saccadic eye movements and Simon task. *Aging Clinical and Experimental Research*. 2019/11/01 2019;31(11):1591-1600.

78. Crawford TJ, Higham S, Mayes J, Dale M, Shaunak S, Lekwuwa G. The role of working memory and attentional disengagement on inhibitory control: effects of aging and Alzheimer's disease. *AGE*. 2013/10/01 2013;35(5):1637-1650.

79. Crawford T. The disengagement of visual attention in Alzheimer's disease: a longitudinal eye-tracking study. Original Research. *Frontiers in Aging Neuroscience*. 2015-June-23 2015;7

80. Crawford TJ, Taylor S, Mardanbegi D, et al. The Effects of Previous Error and Success in Alzheimer’s Disease and Mild Cognitive Impairment. *Scientific Reports*. 2019/12/27 2019;9(1):20204.

81. Garbutt S, Matlin A, Hellmuth J, et al. Oculomotor function in frontotemporal lobar degeneration, related disorders and Alzheimer's disease. *Brain*. May 2008;131(Pt 5):1268-81.

82. Holden JG, Cosnard A, Laurens B, et al. Prodromal Alzheimer’s Disease Demonstrates Increased Errors at a Simple and Automated Anti-Saccade Task. *Journal of Alzheimer's Disease*. 2018;65:1209-1223.

83. Kaufman LD, Pratt J, Levine B, Black SE. Executive deficits detected in mild Alzheimer's disease using the antisaccade task. *Brain and Behavior*. 2012;2(1):15-21.

84. Lage C, López-García S, Bejanin A, et al. Distinctive Oculomotor Behaviors in Alzheimer's Disease and Frontotemporal Dementia. *Front Aging Neurosci*. 2020;12:603790.

85. Laurens B, Planche V, Cubizolle S, et al. A Spatial Decision Eye-Tracking Task in Patients with Prodromal and Mild Alzheimer's Disease. *J Alzheimers Dis*. Aug 12 2019;71(2):613-621.

86. Lenoble Q, Bubbico G, Szaffarczyk S, Pasquier F, Boucart M. Scene Categorization in Alzheimer's Disease: A Saccadic Choice Task. *Dementia and Geriatric Cognitive Disorders Extra*. 2015;5(1):1-12.

87. Lenoble Q, Corveleyn X, Szaffarczyk S, Pasquier F, Boucart M. Attentional capture by incongruent object/background scenes in patients with Alzheimer disease. *Cortex*. 2018/10/01/ 2018;107:4-12.

88. Noiret N, Carvalho N, Laurent É, et al. Saccadic Eye Movements and Attentional Control in Alzheimer's Disease. *Archives of Clinical Neuropsychology*. 2018;33(1):1-13.

89. Pavisic IM, Firth NC, Parsons S, et al. Eyetracking Metrics in Young Onset Alzheimer's Disease: A Window into Cognitive Visual Functions. *Front Neurol*. 2017;8:377.

90. Polden M, Crawford TJ. Eye Movement Latency Coefficient of Variation as a Predictor of Cognitive Impairment: An Eye Tracking Study of Cognitive Impairment. *Vision*. 2023;7(2). doi:10.3390/vision7020038

91. Polden M, Wilcockson TDW, Crawford TJ. The Disengagement of Visual Attention: An Eye-Tracking Study of Cognitive Impairment, Ethnicity and Age. *Brain Sciences*. 2020;10(7). doi:10.3390/brainsci10070461

92. Riek HC, Brien DC, Coe BC, et al. Cognitive correlates of antisaccade behaviour across multiple neurodegenerative diseases. *Brain Communications*. 2023;5(2):fcad049.

93. Shakespeare TJ, Kaski D, Yong KXX, et al. Abnormalities of fixation, saccade and pursuit in posterior cortical atrophy. *Brain*. 2015;138(7):1976-1991.

94. Wilcockson TDW, Mardanbegi D, Xia B, et al. Abnormalities of saccadic eye movements in dementia due to Alzheimer's disease and mild cognitive impairment. *Aging (Albany NY)*. Aug 2 2019;11(15):5389-5398.

95. Tao M, Cui L, Du Y, et al. Analysis of eye movement features in patients with Alzheimer's disease based on intelligent eye movement analysis and evaluation system. *Journal of Alzheimer’s Disease*. 2024;102(4):1249-1259.

96. Bayhan HA, Aslan Bayhan S, Celikbilek A, Tanık N, Gürdal C. Evaluation of the chorioretinal thickness changes in A lzheimer's disease using spectral‐domain optical coherence tomography. *Clinical & experimental ophthalmology*. 2015;43(2):145-151.

97. Gharbiya M, Trebbastoni A, Parisi F, et al. Choroidal thinning as a new finding in Alzheimer's disease: evidence from enhanced depth imaging spectral domain optical coherence tomography. *J Alzheimers Dis*. 2014;40(4):907-17.

98. Bulut M, Yaman A, Erol MK, et al. Choroidal Thickness in Patients with Mild Cognitive Impairment and Alzheimer's Type Dementia. *J Ophthalmol*. 2016;2016:7291257.

99. Trebbastoni A, Marcelli M, Mallone F, et al. Attenuation of choroidal thickness in patients with Alzheimer disease. *Alzheimer Disease & Associated Disorders*. 2017;31(2):128-134.

100. Cunha JP, Proença R, Dias-Santos A, et al. Choroidal thinning: Alzheimer's disease and aging. *Alzheimer's & Dementia: Diagnosis, Assessment & Disease Monitoring*. 2017;8:11-17.

101. Bulut M, Kurtuluş F, Gözkaya O, et al. Evaluation of optical coherence tomography angiographic findings in Alzheimer's type dementia. *Br J Ophthalmol*. Feb 2018;102(2):233-237.

102. Querques G, Borrelli E, Sacconi R, et al. Functional and morphological changes of the retinal vessels in Alzheimer's disease and mild cognitive impairment. *Sci Rep*. Jan 11 2019;9(1):63.

103. Haan J, Kreeke JA, Berckel BN, et al. Is retinal vasculature a biomarker in amyloid proven Alzheimer's disease? *Alzheimer's &amp; Dementia: Diagnosis, Assessment &amp; Disease Monitoring*. 2019;11(1):383-391.

104. López-De-Eguileta A, Lage C, López-García S, et al. Evaluation of choroidal thickness in prodromal Alzheimer’s disease defined by amyloid PET. *PLOS ONE*. 2020;15(9):e0239484.

105. Salobrar-Garcia E, Méndez-Hernández C, Hoz R, et al. Ocular Vascular Changes in Mild Alzheimer's Disease Patients: Foveal Avascular Zone, Choroidal Thickness, and ONH Hemoglobin Analysis. *J Pers Med*. Nov 15 2020;10(4)

106. Robbins CB, Grewal DS, Thompson AC, et al. Choroidal Structural Analysis in Alzheimer Disease, Mild Cognitive Impairment, and Cognitively Healthy Controls. *Am J Ophthalmol*. Mar 2021;223:359-367.

107. Zhang S, Kwapong WR, Yang T, et al. Choriocapillaris Changes Are Correlated With Disease Duration and MoCA Score in Early-Onset Dementia. *Front Aging Neurosci*. 2021;13:656750.

108. Li M, Li R, Lyu J-h, et al. Relationship Between Alzheimer’s Disease and Retinal Choroidal Thickness: A Cross-Sectional Study. *Journal of Alzheimer's Disease*. 2021;80:407-419.

109. Ma JP, Robbins CB, Lee JM, et al. Longitudinal Analysis of the Retina and Choroid in Cognitively Normal Individuals at Higher Genetic Risk of Alzheimer Disease. *Ophthalmol Retina*. Jul 2022;6(7):607-619.

110. Corradetti G, Oncel D, Kadomoto S, et al. Choriocapillaris and Retinal Vascular Alterations in Presymptomatic Alzheimer's Disease. *Investigative Ophthalmology & Visual Science*. 2024;65(1):47-47.

111. Bambo MP, Garcia-Martin E, Gutierrez-Ruiz F, et al. Analysis of optic disk color changes in Alzheimer's disease: A potential new biomarker. *Clinical Neurology and Neurosurgery*. 2015/05/01/ 2015;132:68-73.

112. Berisha F, Feke GT, Trempe CL, McMeel JW, Schepens CL. Retinal abnormalities in early Alzheimer's disease. *Invest Ophthalmol Vis Sci*. May 2007;48(5):2285-9.

113. Chi Y, Wang YH, Yang L. [The investigation of retinal nerve fiber loss in Alzheimer's disease]. *Zhonghua Yan Ke Za Zhi*. Feb 2010;46(2):134-9.

114. Cipollini V, Abdolrahimzadeh S, Troili F, et al. Neurocognitive Assessment and Retinal Thickness Alterations in Alzheimer Disease: Is There a Correlation? *J Neuroophthalmol*. Sep 2020;40(3):370-377.

115. Cunha LP, Lopes LC, Costa-Cunha LV, et al. Macular Thickness Measurements with Frequency Domain-OCT for Quantification of Retinal Neural Loss and its Correlation with Cognitive Impairment in Alzheimer's Disease. *PLoS One*. 2016;11(4):e0153830.

116. Cunha JP, Proença R, Dias-Santos A, et al. OCT in Alzheimer’s disease: thinning of the RNFL and superior hemiretina. *Graefe's Archive for Clinical and Experimental Ophthalmology*. 2017/09/01 2017;255(9):1827-1835.

117. Eraslan M, Cerman E, Cekic O, et al. Neurodegeneration in ocular and central nervous systems: optical coherence tomography study in normal-tension glaucoma and Alzheimer disease. *Turkish Journal of Medical Sciences*. 2015;45(5):1106-1114.

118. Garcia‐Martin E, Bambo MP, Marques ML, et al. Ganglion cell layer measurements correlate with disease severity in patients with A lzheimer's disease. *Acta ophthalmologica*. 2016;94(6):e454-e459.

119. Güneş A, Demirci S, Tök L, Tök Ö, Demirci S. Evaluation of retinal nerve fiber layer thickness in Alzheimer disease usingspectral-domain optical coherence tomography. *Turkish journal of medical sciences*. 2015;45(5):1094-1097.

120. Iseri PK, Altinaş O, Tokay T, Yüksel N. Relationship between cognitive impairment and retinal morphological and visual functional abnormalities in Alzheimer disease. *J Neuroophthalmol*. Mar 2006;26(1):18-24.

121. Jindahra P, Hengsiri N, Witoonpanich P, et al. &lt;p&gt;Evaluation of Retinal Nerve Fiber Layer and Ganglion Cell Layer Thickness in Alzheimer’s Disease Using Optical Coherence Tomography&lt;/p&gt. *Clinical Ophthalmology*. 2020;Volume 14:2995-3000.

122. Kirbas S, Turkyilmaz K, Anlar O, Tufekci A, Durmus M. Retinal nerve fiber layer thickness in patients with Alzheimer disease. *J Neuroophthalmol*. Mar 2013;33(1):58-61.

123. Kromer R, Serbecic N, Hausner L, Froelich L, Aboul-Enein F, Beutelspacher SC. Detection of Retinal Nerve Fiber Layer Defects in Alzheimer's Disease Using SD-OCT. *Front Psychiatry*. 2014;5:22.

124. Larrosa JM, Garcia-Martin E, Bambo MP, et al. Potential new diagnostic tool for Alzheimer's disease using a linear discriminant function for Fourier domain optical coherence tomography. *Investigative ophthalmology & visual science*. 2014;55(5):3043-3051.

125. Lu Y, Li Z, Zhang X, et al. Retinal nerve fiber layer structure abnormalities in early Alzheimer's disease: Evidence in optical coherence tomography. *Neuroscience Letters*. 2010/08/09/ 2010;480(1):69-72.

126. Marziani E, Pomati S, Ramolfo P, et al. Evaluation of retinal nerve fiber layer and ganglion cell layer thickness in Alzheimer's disease using spectral-domain optical coherence tomography. *Invest Ophthalmol Vis Sci*. Sep 5 2013;54(9):5953-8.

127. Moschos MM, Markopoulos I, Chatziralli I, et al. Structural and functional impairment of the retina and optic nerve in Alzheimer's disease. *Curr Alzheimer Res*. Sep 2012;9(7):782-8.

128. Mutlu U, Colijn JM, Ikram MA, et al. Association of Retinal Neurodegeneration on Optical Coherence Tomography With Dementia: A Population-Based Study. *JAMA Neurology*. 2018;75(10):1256-1263.

129. Parisi V, Restuccia R, Fattapposta F, Mina C, Bucci MG, Pierelli F. Morphological and functional retinal impairment in Alzheimer's disease patients. *Clin Neurophysiol*. Oct 2001;112(10):1860-7.

130. Polo V, Garcia-Martin E, Bambo MP, et al. Reliability and validity of Cirrus and Spectralis optical coherence tomography for detecting retinal atrophy in Alzheimer’s disease. *Eye*. 2014/06/01 2014;28(6):680-690.

131. Salobrar-García E, de Hoz R, Ramírez AI, et al. Changes in visual function and retinal structure in the progression of Alzheimer's disease. *PLoS One*. 2019;14(8):e0220535.

132. Shi Z, Wu Y, Wang M, et al. Greater Attenuation of Retinal Nerve Fiber Layer Thickness in Alzheimer's Disease Patients. *Journal of Alzheimer's Disease*. 2014;40:277-283.

133. Trebbastoni A, D’Antonio F, Bruscolini A, et al. Retinal nerve fibre layer thickness changes in Alzheimer’s disease: Results from a 12-month prospective case series. *Neuroscience Letters*. 2016/08/26/ 2016;629:165-170.

134. Yan Y, Wu X, Wang X, et al. The Retinal Vessel Density Can Reflect Cognitive Function in Patients with Alzheimer's Disease: Evidence from Optical Coherence Tomography Angiography. *J Alzheimers Dis*. 2021;79(3):1307-1316.

135. Zabel P, Kaluzny JJ, Wilkosc-Debczynska M, et al. Comparison of Retinal Microvasculature in Patients With Alzheimer's Disease and Primary Open-Angle Glaucoma by Optical Coherence Tomography Angiography. *Invest Ophthalmol Vis Sci*. Aug 1 2019;60(10):3447-3455.

136. Criscuolo C, Cennamo G, Montorio D, et al. Assessment of retinal vascular network in amnestic mild cognitive impairment by optical coherence tomography angiography. *PLoS One*. 2020;15(6):e0233975.

137. Ascaso FJ, Cruz N, Modrego PJ, et al. Retinal alterations in mild cognitive impairment and Alzheimer’s disease: an optical coherence tomography study. *Journal of Neurology*. 2014/08/01 2014;261(8):1522-1530.

138. Cheung CY, Ong YT, Hilal S, et al. Retinal ganglion cell analysis using high-definition optical coherence tomography in patients with mild cognitive impairment and Alzheimer's disease. *J Alzheimers Dis*. 2015;45(1):45-56.

139. Choi SH, Park SJ, Kim NR. Macular Ganglion Cell -Inner Plexiform Layer Thickness Is Associated with Clinical Progression in Mild Cognitive Impairment and Alzheimers Disease. *PLoS One*. 2016;11(9):e0162202.

140. Ferrari L, Huang S-C, Magnani G, Ambrosi A, Comi G, Leocani L. Optical coherence tomography reveals retinal neuroaxonal thinning in frontotemporal dementia as in Alzheimer’s disease. *Journal of Alzheimer's Disease*. 2017;56(3):1101-1107.

141. Gao L, Liu Y, Li X, Bai Q, Liu P. Abnormal retinal nerve fiber layer thickness and macula lutea in patients with mild cognitive impairment and Alzheimer's disease. *Archives of Gerontology and Geriatrics*. 2015/01/01/ 2015;60(1):162-167.

142. Kesler A, Vakhapova V, Korczyn AD, Naftaliev E, Neudorfer M. Retinal thickness in patients with mild cognitive impairment and Alzheimer's disease. *Clin Neurol Neurosurg*. Sep 2011;113(7):523-6.

143. Liu D, Zhang L, Li Z, et al. Thinner changes of the retinal nerve fiber layer in patients with mild cognitive impairment and Alzheimer’s disease. *BMC Neurology*. 2015/02/21 2015;15(1):14.

144. Oktem EO, Derle E, Kibaroglu S, Oktem C, Akkoyun I, Can U. The relationship between the degree of cognitive impairment and retinal nerve fiber layer thickness. *Neurological Sciences*. 2015/07/01 2015;36(7):1141-1146.

145. Paquet C, Boissonnot M, Roger F, Dighiero P, Gil R, Hugon J. Abnormal retinal thickness in patients with mild cognitive impairment and Alzheimer's disease. *Neuroscience letters*. 2007;420(2):97-99.

146. Asanad S, Fantini M, Sultan W, et al. Retinal nerve fiber layer thickness predicts CSF amyloid/tau before cognitive decline. *PLoS One*. 2020;15(5):e0232785.

147. Byun MS, Park SW, Lee JH, et al. Association of Retinal Changes With Alzheimer Disease Neuroimaging Biomarkers in Cognitively Normal Individuals. *JAMA Ophthalmol*. May 1 2021;139(5):548-556.

148. Salobrar-Garcia E, Hoyas I, Leal M, et al. Analysis of Retinal Peripapillary Segmentation in Early Alzheimer's Disease Patients. *Biomed Res Int*. 2015;2015:636548.

149. Golzan SM, Goozee K, Georgevsky D, et al. Retinal vascular and structural changes are associated with amyloid burden in the elderly: ophthalmic biomarkers of preclinical Alzheimer’s disease. *Alzheimer's Research & Therapy*. 2017/03/01 2017;9(1):13.

150. Feke GT, Hyman BT, Stern RA, Pasquale LR. Retinal blood flow in mild cognitive impairment and Alzheimer's disease. *Alzheimers Dement (Amst)*. Jun 2015;1(2):144-51.

151. Pillai JA, Bermel R, Bonner-Jackson A, et al. Retinal nerve fiber layer thinning in Alzheimer’s disease: a case–control study in comparison to normal aging, Parkinson’s disease, and non-Alzheimer’s dementia. *American Journal of Alzheimer's Disease & Other Dementias®*. 2016;31(5):430-436.

152. Yoon SP, Grewal DS, Thompson AC, et al. Retinal Microvascular and Neurodegenerative Changes in Alzheimer's Disease and Mild Cognitive Impairment Compared with Control Participants. *Ophthalmol Retina*. Jun 2019;3(6):489-499.

153. Zhang YS, Onishi AC, Zhou N, et al. Characterization of Inner Retinal Hyperreflective Alterations in Early Cognitive Impairment on Adaptive Optics Scanning Laser Ophthalmoscopy. *Invest Ophthalmol Vis Sci*. Aug 1 2019;60(10):3527-3536.

154. Snyder PJ, Johnson LN, Lim YY, et al. Nonvascular retinal imaging markers of preclinical Alzheimer's disease. *Alzheimer's & Dementia: Diagnosis, Assessment & Disease Monitoring*. 2016/01/01/ 2016;4:169-178.

155. van de Kreeke JA, Nguyen HT, Konijnenberg E, et al. Longitudinal retinal layer changes in preclinical Alzheimer's disease. *Acta Ophthalmol*. Aug 2021;99(5):538-544.

156. van de Kreeke JA, Nguyen HT, den Haan J, et al. Retinal layer thickness in preclinical Alzheimer's disease. *Acta Ophthalmol*. Dec 2019;97(8):798-804.

157. Santos CY, Johnson LN, Sinoff SE, Festa EK, Heindel WC, Snyder PJ. Change in retinal structural anatomy during the preclinical stage of Alzheimer's disease. *Alzheimers Dement (Amst)*. 2018;10:196-209.

158. López-Cuenca I, de Hoz Rd, Salobrar-García E, et al. Macular Thickness Decrease in Asymptomatic Subjects at High Genetic Risk of Developing Alzheimer's Disease: An OCT Study. *Journal of clinical medicine*. 2020;9(6):1728.

159. Danesh-Meyer HV, Birch H, Ku JY, Carroll S, Gamble G. Reduction of optic nerve fibers in patients with Alzheimer disease identified by laser imaging. *Neurology*. Nov 28 2006;67(10):1852-4.

160. Kurna SA, Akar G, Altun A, Agirman Y, Gozke E, Sengor T. Confocal scanning laser tomography of the optic nerve head on the patients with Alzheimer's disease compared to glaucoma and control. *Int Ophthalmol*. Dec 2014;34(6):1203-11.

161. Shao Y, Jiang H, Wei Y, et al. Visualization of Focal Thinning of the Ganglion Cell-Inner Plexiform Layer in Patients with Mild Cognitive Impairment and Alzheimer's Disease. *J Alzheimers Dis*. 2018;64(4):1261-1273.

162. Sharma SN, Marsh JW, Tsipursky MS, Boppart SA. Ratiometric analysis of in vivo optical coherence tomography retinal layer thicknesses for detection of changes in Alzheimer's disease. *Translational Biophotonics*. 2023/08/01 2023;5(3-4):e202300003.

163. Shin JY, Choi EY, Kim M, Lee HK, Byeon SH. Changes in retinal microvasculature and retinal layer thickness in association with apolipoprotein E genotype in Alzheimer's disease. *Sci Rep*. Jan 19 2021;11(1):1847.

164. Lian T-h, Jin Z, Qu Y-z, et al. The Relationship Between Retinal Nerve Fiber Layer Thickness and Clinical Symptoms of Alzheimer's Disease. Original Research. *Frontiers in Aging Neuroscience*. 2021-January-29 2021;12

165. Jiang H, Wei Y, Shi Y, et al. Altered Macular Microvasculature in Mild Cognitive Impairment and Alzheimer Disease. *J Neuroophthalmol*. Sep 2018;38(3):292-298.

166. Farzinvash Z, Abutorabi-Zarchi M, Manaviat M, Zare Mehrjerdi H. Retinal Ganglion Cell Complex in Alzheimer Disease: Comparing Ganglion Cell Complex and Central Macular Thickness in Alzheimer Disease and Healthy Subjects Using Spectral Domain-Optical Coherence Tomography. *Basic Clin Neurosci*. Sep-Oct 2022;13(5):675-684.

167. Yang K, Cui L, Chen X, et al. Decreased Vessel Density in Retinal Capillary Plexus and Thinner Ganglion Cell Complex Associated With Cognitive Impairment. Original Research. *Frontiers in Aging Neuroscience*. 2022-April-26 2022;14

168. O'Bryhim BE, Apte RS, Kung N, Coble D, Van Stavern GP. Association of Preclinical Alzheimer Disease With Optical Coherence Tomographic Angiography Findings. *JAMA Ophthalmol*. Nov 1 2018;136(11):1242-1248.

169. Wu J, Zhang X, Azhati G, Li T, Xu G, Liu F. Retinal microvascular attenuation in mental cognitive impairment and Alzheimer's disease by optical coherence tomography angiography. *Acta Ophthalmologica*. 2020;98(6)

170. Lahme L, Esser EL, Mihailovic N, et al. Evaluation of Ocular Perfusion in Alzheimer's Disease Using Optical Coherence Tomography Angiography. *J Alzheimers Dis*. 2018;66(4):1745-1752.

171. Chua J, Hu Q, Ke M, et al. Retinal microvasculature dysfunction is associated with Alzheimer’s disease and mild cognitive impairment. *Alzheimer's Research & Therapy*. 2020/12/04 2020;12(1):161.

172. Wang X, Zhao Q, Tao R, et al. Decreased retinal vascular density in Alzheimer’s disease (AD) and mild cognitive impairment (MCI): an optical coherence tomography angiography (OCTA) study. *Frontiers in Aging Neuroscience*. 2021:295.

173. van de Kreeke JA, Nguyen HT, Konijnenberg E, et al. Optical coherence tomography angiography in preclinical Alzheimer's disease. *Br J Ophthalmol*. Feb 2020;104(2):157-161.

174. Elahi FM, Ashimatey SB, Bennett DJ, et al. Retinal imaging demonstrates reduced capillary density in clinically unimpaired APOE ε4 gene carriers. *Alzheimers Dement (Amst)*. 2021;13(1):e12181.

175. Jianyang X, Quanyong Y, Yufei W, et al. Deep segmentation of OCTA for evaluation and association of changes of retinal microvasculature with Alzheimer’s disease and mild cognitive impairment. *British Journal of Ophthalmology*. 2024;108(3):432.

176. Ma X, Xie Z, Wang H, et al. A cross-sectional study of retinal vessel changes based on optical coherence tomography angiography in Alzheimer’s disease and mild cognitive impairment. Original Research. *Frontiers in Aging Neuroscience*. 2023-April-11 2023;15

177. Pead E, Thompson AC, Grewal DS, et al. Retinal Vascular Changes in Alzheimer's Dementia and Mild Cognitive Impairment: A Pilot Study Using Ultra-Widefield Imaging. *Transl Vis Sci Technol*. Jan 3 2023;12(1):13.

178. Cabrera DeBuc D, Somfai GM, Arthur E, Kostic M, Oropesa S, Mendoza Santiesteban C. Investigating Multimodal Diagnostic Eye Biomarkers of Cognitive Impairment by Measuring Vascular and Neurogenic Changes in the Retina. *Frontiers in physiology*. 2018;9:1721-1721.

179. Cheung CY-l, Ong YT, Ikram MK, et al. Microvascular network alterations in the retina of patients with Alzheimer's disease. *Alzheimer's & Dementia*. 2014;10(2):135-142.

180. Frost S, Kanagasingam Y, Sohrabi H, et al. Retinal vascular biomarkers for early detection and monitoring of Alzheimer’s disease. *Translational Psychiatry*. 2013;3(2):e233-e233.

181. Williams MA, McGowan AJ, Cardwell CR, et al. Retinal microvascular network attenuation in Alzheimer's disease. *Alzheimers Dement (Amst)*. Jun 2015;1(2):229-235.

182. Einarsdottir AB, Hardarson SH, Kristjansdottir JV, Bragason DT, Snaedal J, Stefánsson E. Retinal oximetry imaging in Alzheimer's disease. *J Alzheimers Dis*. 2016;49(1):79-83.

183. Csincsik L, MacGillivray TJ, Flynn E, et al. Peripheral retinal imaging biomarkers for Alzheimer’s disease: a pilot study. *Ophthalmic research*. 2018;59(4):182-192.

184. de Jong FJ, Schrijvers EMC, Ikram MK, et al. Retinal vascular caliber and risk of dementia. *Neurology*. 2011;76(9):816-821.

185. Sharafi SM, Sylvestre J-P, Chevrefils C, et al. Vascular retinal biomarkers improves the detection of the likely cerebral amyloid status from hyperspectral retinal images. *Alzheimer's & dementia (New York, N Y)*. 2019;5:610-617.

186. Tsai CS, Ritch R, Schwartz B, et al. Optic nerve head and nerve fiber layer in Alzheimer's disease. *Arch Ophthalmol*. Feb 1991;109(2):199-204.

187. Chaitanuwong P, Jariyakosol S, Apinyawasisuk S, et al. Changes in Ocular Biomarkers from Normal Cognitive Aging to Alzheimer's Disease: A Pilot Study. *Eye Brain*. 2023;15:15-23.

188. More SS, Beach JM, Vince R. Early Detection of Amyloidopathy in Alzheimer's Mice by Hyperspectral Endoscopy. *Investigative Opthalmology & Visual Science*. 2016;57(7):3231.

189. More SS, Beach JM, McClelland C, Mokhtarzadeh A, Vince R. In Vivo Assessment of Retinal Biomarkers by Hyperspectral Imaging: Early Detection of Alzheimer’s Disease. *ACS Chemical Neuroscience*. 2019;10(11):4492-4501.

190. Hadoux X, Hui F, Lim JKH, et al. Non-invasive in vivo hyperspectral imaging of the retina for potential biomarker use in Alzheimer’s disease. *Nature Communications*. 2019/09/17 2019;10(1):4227.

191. Lemmens S, Van Craenendonck T, Van Eijgen J, et al. Combination of snapshot hyperspectral retinal imaging and optical coherence tomography to identify Alzheimer’s disease patients. *Alzheimer's Research & Therapy*. 2020/11/10 2020;12(1):144.

192. Lim JKH, Li Q-X, Ryan T, et al. Retinal hyperspectral imaging in the 5xFAD mouse model of Alzheimer’s disease. *Scientific Reports*. 2021/03/18 2021;11(1):6387.

193. Poudel P, Frost SM, Eslick S, et al. Hyperspectral Retinal Imaging as a Non-Invasive Marker to Determine Brain Amyloid Status. *J Alzheimers Dis*. 2024;100(s1):S131-s152.

194. Nunes A, Silva G, Duque C, et al. Retinal texture biomarkers may help to discriminate between Alzheimer’s, Parkinson’s, and healthy controls. *PloS one*. 2019;14(6):e0218826.

195. Wisely CE, Dong W, Ricardo H, et al. Convolutional neural network to identify symptomatic Alzheimer’s disease using multimodal retinal imaging. *British Journal of Ophthalmology*. 2022;106(3):388.

196. Zhang Q, Li J, Bian M, et al. Retinal Imaging Techniques Based on Machine Learning Models in Recognition and Prediction of Mild Cognitive Impairment. *Neuropsychiatr Dis Treat*. 2021;17:3267-3281.

197. Tian J, Smith G, Guo H, et al. Modular machine learning for Alzheimer's disease classification from retinal vasculature. *Scientific Reports*. 2021/01/08 2021;11(1):238.

198. Kim DY, Lim YJ, Park JH, Sunwoo MH. Efficient Deep Learning Algorithm for Alzheimer's Disease Diagnosis using Retinal Images. 2022:254-257.

199. Cheung CY, Ran AR, Wang S, et al. A deep learning model for detection of Alzheimer's disease based on retinal photographs: a retrospective, multicentre case-control study. *Lancet Digit Health*. Nov 2022;4(11):e806-e815.

200. Corbin D, Lesage F. Assessment of the predictive potential of cognitive scores from retinal images and retinal fundus metadata via deep learning using the CLSA database. *Sci Rep*. Apr 6 2022;12(1):5767.
